# Symmetric neural progenitor divisions require chromatin-mediated homologous recombination DNA repair by *Ino80*

**DOI:** 10.1038/s41467-020-17551-4

**Published:** 2020-07-31

**Authors:** Jason M. Keil, Daniel Z. Doyle, Adel Qalieh, Mandy M. Lam, Owen H. Funk, Yaman Qalieh, Lei Shi, Nitesh Mohan, Alice Sorel, Kenneth Y. Kwan

**Affiliations:** 1 Michigan Neuroscience Institute (MNI), University of Michigan, Ann Arbor, MI 48109 USA; 2 Department of Human Genetics, University of Michigan, Ann Arbor, MI 48109 USA; 3 Medical Scientist Training Program, University of Michigan, Ann Arbor, MI 48109 USA; 4 Neuroscience Graduate Program, University of Michigan, Ann Arbor, MI 48109 USA

**Keywords:** Cell division, Chromatin, Transcription, Neuronal development

## Abstract

Chromatin regulates spatiotemporal gene expression during neurodevelopment, but it also mediates DNA damage repair essential to proliferating neural progenitor cells (NPCs). Here, we uncover molecularly dissociable roles for nucleosome remodeler *Ino80* in chromatin-mediated transcriptional regulation and genome maintenance in corticogenesis. We find that conditional *Ino80* deletion from cortical NPCs impairs DNA double-strand break (DSB) repair, triggering p53-dependent apoptosis and microcephaly. Using an in vivo DSB repair pathway assay, we find that *Ino80* is selectively required for homologous recombination (HR) DNA repair, which is mechanistically distinct from *Ino80* function in YY1-associated transcription. Unexpectedly, sensitivity to loss of *Ino80*-mediated HR is dependent on NPC division mode: *Ino80* deletion leads to unrepaired DNA breaks and apoptosis in symmetric NPC-NPC divisions, but not in asymmetric neurogenic divisions. This division mode dependence is phenocopied following conditional deletion of HR gene *Brca2*. Thus, distinct modes of NPC division have divergent requirements for *Ino80*-dependent HR DNA repair.

## Abstract (web summary)

Chromatin mediates transcription and DNA repair. Here, the authors show distinct roles of chromatin remodeler INO80 in expression of YY1-regulated genes and repair of DNA breaks by homologous recombination, a DNA repair pathway important for symmetrically-dividing neural progenitors.

## Introduction

Emerging human genetic findings have implicated dysregulation of chromatin as a contributor to developmental brain disorders^1,2^. Altered chromatin function can perturb neural cell fates, maturation, or plasticity via transcriptional dysregulation^3,4^. Chromatin, however, also plays key roles in DNA-damage repair^5,6^. How disruption of chromatin-mediated genome maintenance affects brain development and whether it contributes to neurodevelopmental disorders remain largely unexplored.

Safeguarding genome integrity in proliferating neural progenitor cells (NPCs) is essential for neurodevelopment^7,8^. In fetal cerebral cortex, neurons and glia are generated by NPCs via successive rounds of cell division^9,10^. In this process, genome damage, notably DNA double-strand breaks (DSBs), inevitably arises during DNA synthesis and mitosis^7^. DSBs are mainly repaired by one of two major pathways: homologous recombination (HR) and nonhomologous end joining (NHEJ)^11–13^. HR uses homologous sequence in S and G2 phases of the cell cycle to seamlessly repair DSBs, whereas NHEJ ligates free DNA ends throughout the cell cycle in a homology-independent manner that can introduce indels or structural rearrangements^11^. DSB repair occurs in the context of chromatin. Repair pathway choice and efficiency are regulated by nucleosome remodelers that control the accessibility and mobility of chromatin^5,6^. Disruption of chromatin regulators may therefore contribute to neurodevelopmental disorders by impairing DNA repair in dividing NPCs, which could trigger DNA-damage response or lead to brain somatic mutations. Although emerging evidence suggests that somatic mutations can contribute to disorders of the brain^14^, whether post-zygotic mutations can arise as a result of chromatin dysregulation remains unknown.

Here, we uncover mechanistically distinct roles of *Ino80* in chromatin-mediated transcriptional regulation and genome maintenance in corticogenesis. *Ino80* encodes the catalytic subunit of the INO80 complex that mediates nucleosome remodeling and histone variant exchange in gene regulation and DNA repair^15–19^. *INO80* was recently identified as a candidate gene for microcephaly and intellectual disability^20^. The neurodevelopmental roles of INO80 and how its disruption contributes to disordered brain development had not been explored.

We find that conditional deletion of *Ino80* from embryonic cortical NPCs leads to accumulation of unrepaired DSBs, which trigger p53 target activation, robust apoptotic responses, and microcephaly. These *Ino80* deletion phenotypes are extensively rescued following co-deletion of *Trp53*, confirming their dependence on p53 response to DNA damage. Using an in vivo assay for DSB repair pathway choice, we find that *Ino80* is selectively required for HR DNA repair, which is mechanistically distinct from *Ino80* function in YY1-associated transcriptional regulation. Surprisingly, NPC sensitivity to loss of *Ino80*-mediated HR is not universal. At the onset of neurogenesis, NPCs transition from symmetric NPC–NPC divisions to asymmetric neurogenic divisions^9,10^. By systematic deletion of *Ino80* from NPCs pre-, peri-, and post transition, we find that deletion of *Ino80* during exclusively symmetric divisions leads to unrepaired DNA breaks and widespread apoptosis. In contrast, deletion of *Ino80* after NPC transition to asymmetric divisions does not. Consistent with a requirement for HR DNA repair selectively in symmetrically dividing NPCs, conditional deletion of well-characterized HR gene *Brca2*^21^ phenocopies the division mode dependence of *Ino80* deletion. Thus, *Ino80* plays mechanistically dissociable roles in chromatin-mediated gene regulation and DNA repair in corticogenesis, and distinct modes of NPC division have divergent requirements for HR.

## Results

### Neuroanatomical defects following *Ino80* deletion from NPCs

In developing forebrain, *Ino80* is expressed on embryonic day (E)11.5, throughout the neurogenic period, and at birth^22^ (Supplementary Fig. 1a). By immunoblotting, we found INO80 expression in developing cortex at E12.5, E17.5, postnatal day (P)2, and P7 (Supplementary Fig. 1b). Constitutive *Ino80* deletion causes embryonic lethality between E8.5 and E10.5^23,24^. We therefore leveraged a conditional *Ino80* allele^24^ to study *Ino80* in corticogenesis.

To distinguish potential *Ino80* functions in proliferating NPCs versus postmitotic neurons, we used two complementary Cre lines for *Ino80* deletion. *Emx1*^*Cre*^ mediates recombination in cortical NPCs starting at E10.5^25^, near the onset of excitatory neurogenesis, thus affecting subsequent NPCs, neurons, and astrocytes of *Emx1* lineage. Deletion of *Ino80* was confirmed by immunoblotting, which revealed loss of INO80 from E12.5 *Emx1*^*Cre/+*^;*Ino80*^*fl/fl*^ (cKO-E) cortex (Supplementary Fig. 1c). *Neurod6*^*Cre*^ (*Nex*^*Cre*^) mediates recombination in newly postmitotic excitatory neurons and spares NPCs^26^. To visualize cells that have undergone recombination, we used the Cre-dependent fluorescent reporter allele *ROSA*^*nT-nG*^ ^27^.

**Fig. 1:**
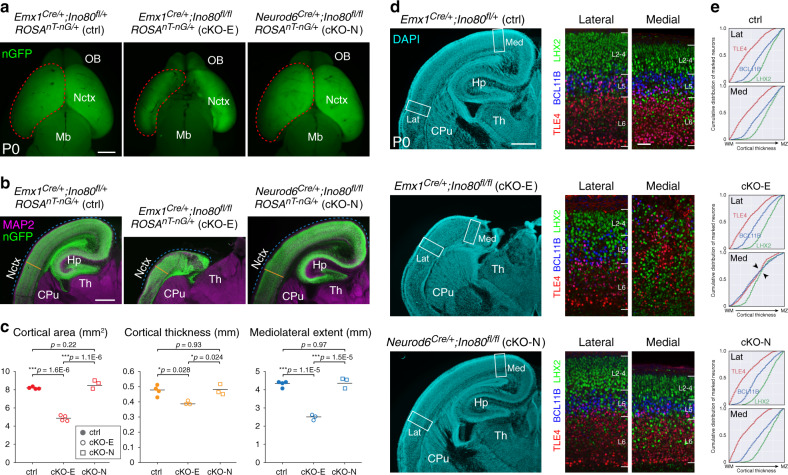
Microcephaly and disrupted medial corticogenesis following *Ino80* deletion from NPCs. **a** Dorsal view of whole-mount P0 control (ctrl) and *Ino80* conditional mutant (cKO) brains. Nuclear (n)GFP (green) was expressed Cre-dependently from *ROSA*^*nT-nG*^. *Emx1*^*Cre*^-mediated *Ino80* deletion from cortical NPCs (cKO-E) led to microcephaly, whereas *Neurod6*^*Cre*^-mediated *Ino80* deletion from postmitotic excitatory neurons (cKO-N) did not. Sample measurements of cortical area (red) quantified in **c** are indicated (ctrl: *n* = 4, cKO-E: *n* = 4, cKO-N: *n* = 3 animals). OB olfactory bulb, Nctx neocortex, Mb midbrain. **b** MAP2 (magenta) and nGFP (green) immunostaining of coronal P0 brain sections. cKO-E, but not cKO-N, was characterized by microcephaly and severe hippocampal hypoplasia. Sample measurements of cortical thickness (yellow) and mediolateral extent (blue) quantified in **c** are indicated (ctrl: *n* = 4, cKO-E: *n* = 3, cKO-N: *n* = 3 animals). CPu caudate putamen, Hp hippocampus, Th thalamus. **c** Cortical area (red), thickness (yellow), and mediolateral extent (blue) were each significantly decreased in cKO-E, but not cKO-N, compared with ctrl (data are mean, one-way ANOVA with Tukey’s post hoc test, Cortical area, ctrl: *n* = 4, cKO-E: *n* = 4, cKO-N: *n* = 3 animals, thickness and mediolateral extent, ctrl: *n* = 4, cKO-E: *n* = 3, cKO-N: *n* = 3 animals). **d** DAPI staining (cyan) of coronal P0 sections revealed altered lamination of medial neocortex and severe hypoplasia of hippocampus in cKO-E (*n* = 3 animals). Analyzed by marker immunostaining (insets), the lamination of LHX2 + (L2-5, green), BCL11B + (L5, blue), and TLE4 + (L6, red) neurons was correctly ordered in lateral (Lat) neocortex of cKO-E, but severely disrupted in medial (Med) neocortex. In cKO-N, normal lamination was present in medial and lateral neocortex. **e**, Analysis of cumulative distribution of layer marker-expressing neurons through thickness of cortex from white matter (WM) to marginal zone (MZ) revealed disrupted lamination in medial cKO-E cortex (*n* = 3 animals). Scale bar: 1 mm in **a**; 500 μm in **b**, **d**; 50 μm in **d** inset.

Following *Ino80* deletion from NPCs (cKO-E), or excitatory neurons (*Neurod6*^*Cre/+*^;*Ino80*^*fl/fl*^, cKO-N), mice were viable at birth, and fertile as adults. On P0, cKO-E, but not cKO-N, was microcephalic, with significant decreases in cortical area, thickness, and mediolateral extent (Fig. 1a–c). In addition, cKO-E cortex showed agenesis of corpus callosum and hypoplasia of hippocampus (Fig. 1b; Supplementary Fig. 1d–f). In contrast to cKO-E, *Ino80* deletion from neurons (cKO-N) did not lead to microcephaly, callosal defects, or hippocampal hypoplasia (Fig. 1a–c; Supplementary Fig. 1e, f). Thus, *Ino80* functioned in NPCs during corticogenesis.

### *Ino80* deletion from NPCs disrupted medial corticogenesis

To assess neocortical lamination, we analyzed layer markers by immunostaining. This revealed a striking mediolateral regional difference in layer formation in *Ino80* cKO-E. In lateral neocortex, TLE4 + layer (L)6, BCL11B(CTIP2) + L5, and LHX2 + L2–5 neurons were properly ordered in cKO-E (Fig. 1d). Analysis of cumulative distribution of neurons labeled by each marker through the thickness of the cortex revealed correct lamination in both ctrl and cKO-E lateral cortex (Lat, Fig. 1e). cKO-E medial cortex, however, was characterized by disrupted layer organization (Med, Fig. 1d, e). The consequences of *Ino80* deletion from NPCs were therefore regionalized and graded on the mediolateral axis in cKO-E; lateral neocortex was grossly normal in lamination, medial neocortex was significantly disorganized, and hippocampus, a cortical structure medial to neocortex, was severely hypoplastic. In contrast, postmitotic deletion of *Ino80* in cKO-N did not alter medial or lateral neocortical lamination (Fig. 1d, e). Together, these data suggested that *Ino80* deletion from NPCs, but not neurons, preferentially disrupted medial corticogenesis.

### Loss of medial NPCs to apoptosis following *Ino80* deletion

**Fig. 2:**
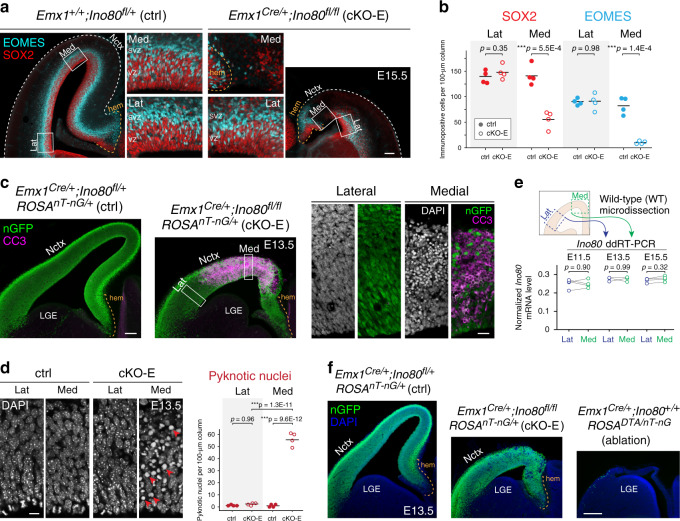
Selective loss of medial NPCs to apoptosis following *Ino80* deletion. **a**, **b** NPC marker immunostaining of coronal E15.5 sections revealed significant loss of SOX2 + apical progenitors (red) and EOMES + intermediate progenitors (cyan) in medial, but not lateral, cKO-E neocortex compared with ctrl (data are mean, two-tailed unpaired *t* test, *n* = 4 animals). NPC marker expression was unaffected in cortical hem in cKO-E. VZ ventricular zone, SVZ subventricular zone. **c** Cleaved Caspase 3 (CC3) immunostaining (magenta) of coronal E13.5 sections showed extensive apoptosis in medial, but not lateral, cKO-E neocortex. Cre-dependent expression of nGFP (green) from *ROSA*^*nT-nG*^ was present in both medial and lateral cKO-E neocortex (*n* = 3 animals). DAPI staining (inset, white) revealed pyknotic nuclei, another marker of apoptosis, in medial, but not lateral, cKO-E neocortex. Neither CC3 immunostaining nor pyknotic nuclei were increased in cKO-E cortical hem. LGE lateral ganglionic eminence. **d** At E13.5, pyknotic nuclei (red arrowheads) were significantly increased in medial, but not lateral, cKO-E neocortex compared with ctrl (data are mean, ANOVA with Tukey’s post hoc test, *n* = 4 animals). **e** Quantitative analysis of *Ino80* mRNA expression in wild-type (WT) embryonic neocortex by droplet digital (dd)RT-PCR revealed consistent *Ino80* expression in lateral and medial neocortex at E11.5, E13.5, and E15.5 (data are mean, two-tailed paired *t* test, E11.5: *n* = 4, E13.5: *n* = 3, E15.5: *n* = 4 animals). **f**, Comparison of ctrl, cKO-E, and *Emx1*^*Cre*^*;ROSA*^*DTA/nT-nG*^ E13.5 cortex (*n* = 3 animals). In ctrl, Cre-dependent nGFP reporter (green) expression was present in both medial and lateral neocortex, and cortical hem. Similarly, in cKO-E, nGFP was present in both medial neocortex affected by apoptosis, and lateral neocortex unaffected by apoptosis. In *Emx1*^*Cre*^*;ROSA*^*DTA/nT-nG*^, both medial and lateral neocortex, as well as cortical hem, were extensively ablated by Cre-dependent expression of DTA, consistent with uniform *Emx1*^*Cre*^ activity throughout the cortex. Scale bar: 100 μm in **a**, **c**; 20 μm in **c** inset; 10 μm in **d**; 200 μm in **f**.

The cKO-E phenotypes implicated *Ino80* function in NPCs. In E15.5 cKO-E cortex, SOX2 + apical progenitors (APs) and EOMES(TBR2) + intermediate progenitors (IPs) were each significantly reduced in number in medial, but not lateral, cortex (Fig. 2a, b). Analysis of S-phase NPCs by a 1-h pulse of thymidine analog ethynyl deoxyuridine (EdU) confirmed selective loss of cycling NPCs in medial cortex in cKO-E by E13.5 (Supplementary Fig. 2a). This regional NPC loss did not affect the cortical hem, a signaling center (Fig. 2a). Next, we assessed whether NPC loss was associated with apoptosis. At E13.5, cleaved-caspase 3 (CC3), a marker of apoptosis, and pyknosis, condensation of chromatin during apoptosis, were widespread in medial, but not lateral, cortex (Fig. 2c, d). Together, these results showed that the perinatal anatomical defects in medial cortex correlated with medial apoptosis in embryonic cKO-E cortex.

The mediolateral difference in apoptosis in cKO-E embryonic cortex was remarkably consistent, showing a similar pattern in all animals we analyzed (Supplementary Fig. 2b, c). To determine whether this was a reflection of spatial difference in normal *Ino80* expression in embryonic cortex, we microdissected medial and lateral cortex from E11.5, E13.5, and E15.5 wild-type embryos and used droplet digital (dd)RT-PCR to analyze *Ino80* mRNA levels (Fig. 2e). This revealed no regional difference in *Ino80* expression between medial and lateral cortex.

To ascertain that the spatial sensitivity to *Ino80* deletion was independent of potential nonuniformity in *Emx1*^*Cre*^ activity, we used the Cre-dependent reporter gene *ROSA*^*nT-nG*^ to analyze Cre recombination, which showed uniform mediolateral expression of nuclear (n)GFP (Fig. 2c, f; Supplementary Fig. 2b, c). This is consistent with other studies using *Emx1*^*Cre*^, a widely used Cre driver line with over 700 citations^25^. Furthermore, we analyzed the extent of cell ablation by *Emx1*^*Cre*^ and Cre-dependent suicide gene *ROSA*^*DTA*^^28^, which led to cell ablation throughout the mediolateral extent of *Emx1*^*Cre*^*;ROSA*^*DTA/nT-nG*^ cortex (Fig. 2f). Together, these data indicated that cKO-E phenotypes were not the result of nonuniform Cre activity or regionalized *Ino80* expression, and implicated an alternative explanation for the preferential sensitivity of medial cortex to *Ino80* deletion.

### Transcriptomic signature of p53 activation in *Ino80* cKO-E

To gain mechanistic insights into the sensitivity of medial NPCs to *Ino80* deletion, we explored molecular functions of *Ino80*. As a chromatin remodeler, INO80 plays a role in transcriptional regulation^17,18^. We performed transcriptome analysis of E13.5 cortex by unique molecular identifier (UMI) RNA-seq using Click-seq^29^. In UMI RNA-seq, Click addition of UMI tag to each cDNA molecule enabled post-sequencing deduplication^30^. Analysis of spike-in ERCC standards revealed excellent quantification, and deletion of *Ino80* exons 2–4 by *Emx1*^*Cre*^ was confirmed (Supplementary Fig. 3a–c).

**Fig. 3:**
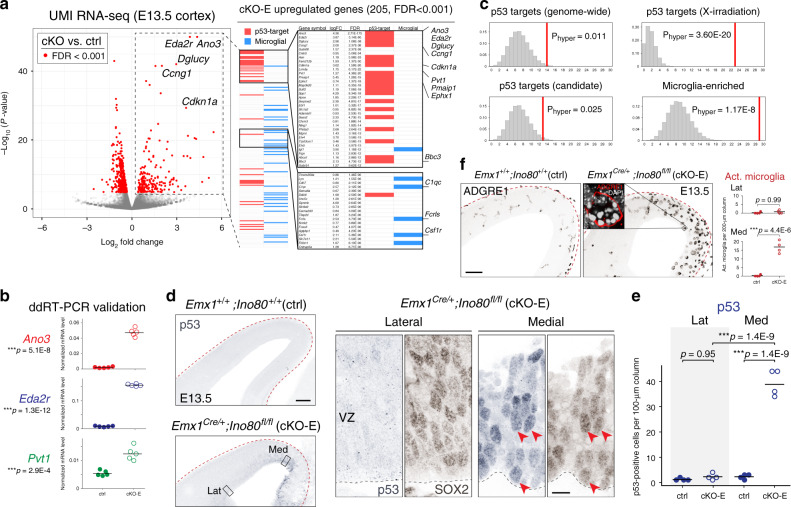
p53 and microglial activation in medial cortex following *Ino80* deletion. **a** Volcano plot of unique molecular identifier (UMI) RNA-seq comparing E13.5 cortex of cKO-E (*n* = 5 animals) with ctrl (*n* = 7 animals). For each gene, *P* value was calculated with likelihood ratio tests and false discovery rate (FDR) was calculated using the Benjamini–Hochberg procedure. Differentially expressed genes (FDR < 0.001) are indicated by red dots. Of the 205 significantly upregulated genes in cKO-E, 36 are known p53 targets (red bars) and 31 are expressed selectively in microglia (blue bars). **b** ddRT-PCR validated significant upregulation of p53-target genes *Ano3*, *Eda2r*, and *Pvt1* in cKO-E compared with ctrl E13.5 cortex (data are mean, two-tailed unpaired *t* test, *n* = 5 animals). **c** Intersectional analysis of the 205 upregulated genes in cKO-E revealed significant enrichment of p53 targets and microglia genes. The distribution of random overlap is shown in histogram, and the observed overlap is indicated by vertical red line (hypergeometric test, Bonferroni correction, α = 0.0125). The subset of p53-target genes upregulated by X-irradiation-induced DSBs were especially overrepresented in cKO-E-upregulated genes. **d**, **e** p53 immunostaining (blue) revealed significant increase in the number of p53-positive cells in medial, but not lateral, cKO-E E13.5 neocortex compared with ctrl (data are mean, one-way ANOVA with Tukey’s post hoc test, *n* = 4 animals). p53 activation was present in SOX2 + (brown) NPCs (solid arrowheads) in cKO-E. **f** ADGRE1 (F4/80) immunostaining revealed an increase in morphologically activated (Act.) microglia in medial, but not lateral, cKO-E neocortex at E13.5 (data are mean, two-tailed unpaired *t* test, *n* = 4 animals). ADGRE1 + microglia (red, inset) phagocytosed numerous pyknotic nuclei labeled by DAPI (white). Scale bar: 100 μm in **d**, **f**; 10 μm in **d** inset.

Analysis of differential gene expression using edgeR^31^ revealed 205 significantly upregulated and 418 significantly downregulated genes in cKO-E with a stringent false discovery rate (FDR) of <0.001 (Fig. 3a; Supplementary Tables 1 and 2). Strikingly, of the 205 upregulated genes, 36 are known to be transcriptionally activated by tumor suppressor protein p53 (TRP53), bound at their genomic locus by p53, or both. p53 is activated in response to DNA damage and turns on target genes that mediate cell-cycle arrest, DNA repair, or apoptosis^32^. We used ddRT-PCR and validated upregulation of three p53-target genes (Fig. 3b). To determine whether p53 targets were overrepresented, we intersected cKO-E-upregulated genes with known p53 targets. Analysis of hypergeometric distribution revealed significant enrichment of p53-target genes identified by genome-wide studies^32^ (Fig. 3c). p53 can upregulate diverse target genes depending on the trigger for activation. Intersection of cKO-E-upregulated genes with p53 targets activated by X-radiation-induced DSBs^33^ revealed a highly significant overrepresentation (*P*_hyper_ = 3.60E-20). Therefore, our transcriptomic analysis uncovered a signature of p53 activation that was consistent with DSBs as a trigger for p53. In an unactivated state, p53 is monoubiquitinated by MDM2 and degraded. DSBs trigger abrogation of MDM2–p53 interaction, thus blocking degradation and stabilizing p53. Consistent with p53 stabilization, nuclear p53 immunostaining was significantly increased 17-fold in E13.5 medial, but not lateral, cortex (Fig. 3d, e), a gradient highly similar to that of apoptosis in cKO-E. We also found a signature consistent with microglia in cKO-E; 31 of the 205 upregulated genes are selectively expressed in microglia in wild-type brain (Fig. 3a, c)^34^. Microglia are resident phagocytes of the brain and can increase in number and undergo activation in response to apoptosis^30,35^. Immunostaining for microglia marker ADGRE1 (F4/80) showed a significant increase in number of activated microglia with phagocytic morphology in medial, but not lateral, cortex (Fig. 3f), consistent with the regional gradient of apoptosis in cKO-E. Together, the p53 transcriptome signature and spatial specificity of p53 stabilization suggested that p53 activation underpinned apoptosis, NPC loss, and anatomical defects in medial cKO-E cortex. Furthermore, the significant overrepresentation of X-radiation p53-target genes was consistent with DSBs as trigger for p53 activation.

### Unrepaired DNA double-strand breaks in *Ino80* cKO-E

**Fig. 4:**
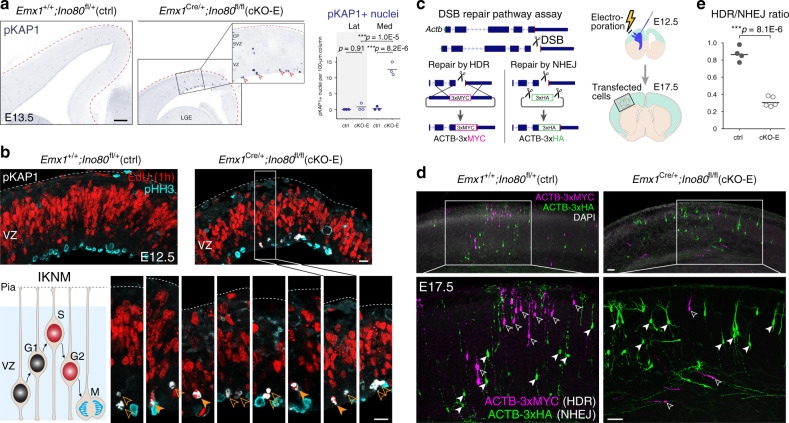
Impaired HR DNA repair and unrepaired DSBs following *Ino80* deletion. **a** Immunostaining for DSB marker pKAP1 (blue) revealed a significant increase in unrepaired DSBs in medial, but not lateral, E13.5 cKO-E neocortex (data are mean, one-way ANOVA with Tukey’s post hoc test, *n* = 3 animals). A majority of pKAP1 + nuclei in cKO-E were positioned near the ventricular surface (open arrowheads). CP cortical plate. **b** Analysis of S-phase NPCs by 1-h pulse of thymidine analog EdU (red), M-phase NPCs by phospho-H3 (pHH3) immunostaining (cyan), and DSBs by pKAP1 immunostaining (white) in E12.5 cortex. NPCs undergo interkinetic nuclear migration (IKNM) and their nuclear position in distinct phases of the cell cycle is depicted in the schematic. pKAP1 staining revealed unrepaired DSBs in 1-h EdU-positive (solid arrowheads, S-phase) and EdU-negative (open arrowheads, post-S-phase) cells near the ventricular surface. Ventricular pKAP1 staining did not colocalize with pHH3, suggesting that NPCs with unrepaired DSBs did not progress successfully into mitosis. **c** Schematic illustration of in vivo DSB repair assay. A genomic DSB is induced by CRISPR–Cas9 within the coding region of *Actb* near the C terminus. Two reporter repair templates were designed such that repair of the DSB by HDR would lead to in-frame expression of ACTB-3xMYC, whereas repair by NHEJ would lead to expression of ACTB-3xHA. CRISPR–Cas9 and reporter repair constructs were co-transfected into cortical NPCs at E12.5 by in utero electroporation (IUE). Electroporated brains were analyzed at E17.5. **d**, **e** In electroporated E17.5 brains, MYC tag-labeled cells (magenta) have undergone HDR of the *Actb* DSB (open arrowheads), whereas HA tag-labeled cells (green) have undergone NHEJ repair (solid arrowheads). Quantification of HDR/NHEJ ratio demonstrated a significant decrease in HDR relative to NHEJ in cKO-E cortex compared with ctrl (data are mean, two-tailed unpaired *t* test, ctrl: *n* = 4, cKO-E: *n* = 5 animals, > 100 cells per animal). Scale bar: 100 μm in **a**; 10 μm in **b**; 50 μm in **d**.

To assess DSBs, we immunostained for phospho-(p)KAP1 (TRIM28), a marker of unrepaired heterochromatic DSBs^36^. At E13.5, cKO-E was characterized by a marked 38-fold increase in pKAP1 + cells in medial cortex (arrowheads, Fig. 4a), but not lateral cortex. Notably, pKAP1 immunostaining was largely localized near the ventricular surface. During corticogenesis, apical NPCs (radial glia) undergo interkinetic nuclear migration (IKNM), where NPC nuclei migrate to upper VZ for S phase and ventricular surface for mitosis. pKAP1 localization in cKO-E suggested that NPCs in G2 or M phase carried unrepaired DSBs. We next co-immunostained pKAP1 (white, Fig. 4b) with a 1-h pulse of EdU (red), a marker of S phase, and phospho-histone H3 (pHH3, cyan), a marker of mitosis. pKAP1 was present in 1-h EdU-positive (S-phase, solid arrowheads) and EdU-negative (post-S-phase, open arrowheads) cells near the ventricular surface, but did not colocalize with pHH3. Together, these data suggested that *Ino80* cKO-E NPCs accumulated, in late S or G2 phase, DSBs that were not properly repaired, impairing or delaying progression through the cell cycle into mitosis.

Increased accumulation of DSBs in cKO-E could result from increased DNA damage or compromised DNA repair. We immunostained for histone variant γH2AX, which is recruited to DSBs, with 1-h EdU (magenta, Supplementary Fig. 4a). This revealed that S-phase NPCs were normally characterized by an abundance of γH2AX foci (green), consistent with a previous study that ~50 DSBs are sustained by each normal cell division^37^. To quantify S-phase DSBs, we co-immunostained for γH2AX and the DNA repair protein 53BP1, which co-localize at DSBs (Supplementary Fig. 4b). This revealed no increase in γH2AX + /53BP1 + foci in EdU + NPCs in E12.5 cKO-E, suggesting that *Ino80* deletion did not induce additional DNA breaks in S phase. In contrast, many post-S-phase (EdU-negative) ventricular NPCs were characterized by pan-nuclear γH2AX staining, a marker of apoptosis following substantial DNA damage during replication^38^ (Supplementary Fig. 4a, c). Together, these data suggested that in the absence of *Ino80*, no additional DNA damage occurred during replication, but repair of synthesis-associated DNA damage was impaired in dividing NPCs, leading to accumulation of unrepaired DSBs and p53-dependent apoptosis in cKO-E medial cortex.

### Impaired homologous recombination DNA repair in *Ino80* cKO-E

INO80 has been shown to mediate removal of histone subunit H2A.Z and exchange of RPA for RAD51, important steps in DSB repair by HR^39,40^. To assess HR in cKO-E, we designed an assay to interrogate DSB repair pathway choice in vivo. We used CRISPR–Cas9 to generate a DSB at the C terminus of *Actb* coding region^41^ and provided two reporter repair templates. The homology-dependent repair (HDR) template contained 800-bp homology arms flanking a 3xMYC epitope tag. HDR, a reliable proxy for endogenous HR^42^, would lead to in-frame knock-in, resulting in ACTB-3xMYC expression (Fig. 4c). The NHEJ template contained sgRNA target sites flanking a 3xHA epitope tag. CRISPR–Cas9 cleavage would generate a short repair template without homology, and NHEJ repair in forward orientation would lead to ACTB-3xHA expression. By providing both repair templates, relative pathway choice can be assessed using an HDR/NHEJ ratio^42^.

To perform the assay in vivo, we used in utero electroporation (IUE) to transfect CRISPR–Cas9 and repair template constructs into E12.5 cortical NPCs (Fig. 4c). Analyzed at E17.5, control brains showed an HDR/NHEJ ratio of 0.87 (Fig. 4d, e). In cKO-E, the HDR/NHEJ ratio was 0.31, a significant 64% decrease. These results support a selective impairment of HR in *Ino80* cKO-E and are consistent with previous studies demonstrating INO80 function in H2A.Z removal and RPA/RAD51 exchange during HR^39,40,43^. Our analysis of E13.5 cKO-E transcriptome revealed no significant change in expression of HR genes (Supplementary Fig. 4d), suggesting that *Ino80* function in HR was not mediated via transcriptional regulation. Together, our data indicated that following *Ino80* deletion, disruption of HR DNA repair led to an accumulation of DSBs in NPCs.

### *Trp53* co-deletion extensively rescued *Ino80* cKO-E phenotypes

**Fig. 5:**
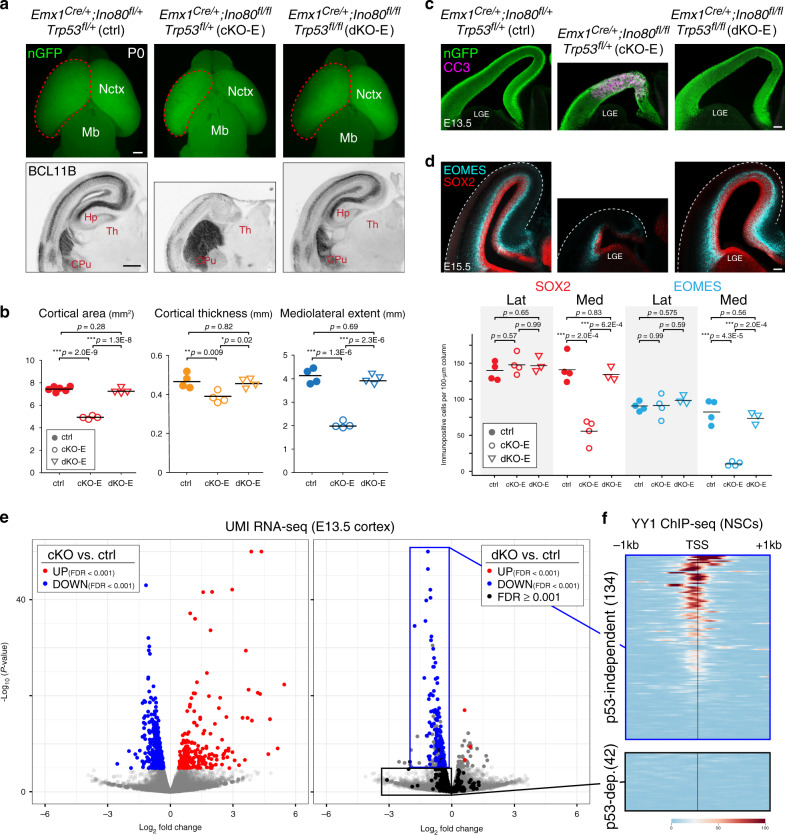
*Trp53* co-deletion rescued *Ino80* phenotypes and revealed mechanistically distinct *Ino80* roles. **a** Dorsal view of P0 whole-mount brains and BCL11B immunostaining (black) of P0 coronal sections. Co-deletion of *Trp53* with *Ino80* (dKO-E) rescued major *Ino80* deletion (cKO-E) phenotypes, including microcephaly, severe hippocampal hypoplasia, and disrupted neocortical lamination. Sample measurements quantified in **b** are indicated (ctrl cortical area: *n* = 6, all other measurements: *n* = 4 animals). **b** Cortical area (red), thickness (yellow), and mediolateral extent (blue) were restored in dKO-E and not significantly different compared with ctrl (data are mean, one-way ANOVA with Tukey’s post hoc test, ctrl cortical area: *n* = 6, all other measurements: *n* = 4 animals). **c** CC3 immunostaining (magenta) revealed no increase in apoptosis in dKO-E E13.5 neocortex (*n* = 3 animals). **d** The number of SOX2 + (red) and EOMES + (cyan) NPCs in E15.5 dKO-E cortex were restored to levels not significantly different compared with ctrl (data are mean, one-way ANOVA with Tukey’s post hoc test, ctrl: *n* = 4, cKO-E: *n* = 4, dKO-E: *n* = 3 animals). **e** UMI RNA-seq volcano plots comparing cKO-E with ctrl, and dKO-E (*n* = 7 animals) with ctrl E13.5 cortex. In the cKO-E versus ctrl comparison (left panel), differentially regulated genes are indicated (upregulated, red dots; downregulated, blue dots). The same genes are labeled in the dKO-E versus ctrl comparison (right panel). Genes that remained differentially regulated (FDR < 0.001) following *Trp53* co-deletion are indicated by dots of the same color. Genes that lost differential expression (FDR ≥ 0.001) are indicated by black dots. The vast majority of cKO-E-upregulated genes (202/205) were rescued by *Trp53* co-deletion, indicating that their upregulation was p53-dependent. About one-third of cKO-E-downregulated genes (134/418), however, remained significantly downregulated in dKO-E, consistent with p53-independent gene regulation by *Ino80*. **f** Intersectional analysis of the 134 p53-independent downregulated genes with published ChIP-seq data from mouse neural stem cells (NSCs) revealed an enrichment of genes bound at their transcriptional start site (TSS) by transcription factor YY1, a known binding partner of INO80. This enrichment was absent from the 42 cKO-E downregulated genes that were reversed by *Trp53* co-deletion. Scale bar: 500 μm in **a**; 100 μm in **c**, **d**.

As a nucleosome remodeler, *Ino80* is positioned to regulate gene expression and DNA repair. To distinguish these distinct aspects of *Ino80* function in contributing to cKO-E phenotypes, we simultaneously deleted *Trp53* (the p53 gene) with *Ino80* (*Emx1*^*Cre/+*^*;Ino80*^*fl/fl*^*;Trp53*^*fl/fl*^; dKO-E) to block p53-dependent response to DNA damage. Co-deletion of *Trp53* led to a strikingly complete rescue of cKO-E phenotypes. At P0, microcephaly (Fig. 5a, b), hippocampal hypoplasia, callosal agenesis, and medial cortex laminar defects (Supplementary Fig. 5a–c) were each markedly rescued by *Trp53* co-deletion in dKO-E. At E13.5, dKO-E cortex showed no increase in apoptosis or activated microglia compared with control (Fig. 5c; Supplementary Fig. 5d, e), and at E15.5, no loss of NPCs (Fig. 5d). This extensive rescue following *Trp53* co-deletion indicated that these *Ino80* cKO-E phenotypes were underpinned by *Ino80* function in DNA repair and p53-dependent response to the resulting DNA damage.

### Dissociable roles of *Ino80* in transcription and DNA repair

Our transcriptome analysis of *Ino80* cKO-E revealed p53-target activation and microglial gene expression. Using *Ino80* cKO-E, potential direct transcriptional effects of *Ino80* could not be isolated from those related to p53 activation. *Ino80*/*Trp53* dKO-E, however, could be leveraged to identify primary effects of *Ino80* on expression. We performed UMI RNA-seq of E13.5 *Ino80*/*Trp53* dKO-E cortex (*n* = 7 animals) for direct comparison with cKO-E (Fig. 5e; Supplementary Tables 3 and 4). In single *Ino80* cKO-E, a majority of upregulated genes were attributed to p53 activation or microglia. In double *Ino80*/*Trp53* dKO-E, these transcriptomic signatures were nearly completely reversed; only 3/205 genes remained significantly upregulated (Fig. 5e). The increased expression of these genes in cKO-E was therefore p53-dependent. In contrast to upregulated genes, a substantial subset of downregulated genes from cKO-E (134/418) remained significantly downregulated in dKO-E (Fig. 5e). The expression of these 134 genes was therefore p53-independent and potentially under direct *Ino80* regulation. Previous studies have shown that INO80 binds YY1 and mediates YY1-associated gene expression^17,44^. Intersectional analysis with available YY1 ChIP-seq data from mouse NPCs^45^ revealed that, of the 134 downregulated genes unaffected by *Trp53* co-deletion (down in both cKO-E and dKO-E, p53-independent, Supplementary Table 5) 67% were characterized by a YY1 peak within 500 bp of the transcriptional start site (TSS, Fig. 5f; Supplementary Fig. 5f, g), typical of YY1-regulated genes. In contrast, for five sets of randomly selected genes, only 10–18% of genes were characterized by a YY1 peak (Supplementary Fig. 5f, g). Thus, *Ino80* regulated YY1-associated transcription independently of p53, thereby playing mechanistically dissociable roles in gene expression and DNA repair during corticogenesis (Supplementary Fig. 6). Importantly, despite persistence of YY1-associated transcriptomic dysregulation in *Ino80*/*Trp53* dKO-E, we found no overt cellular or anatomical phenotypes (Fig. 5; Supplementary Fig. 5). The robust apoptosis and microcephaly in *Ino80* cKO-E were therefore consequences of p53-dependent responses to impaired DNA repair.

INO80 is a chromatin remodeler. To assess whether genome-wide chromatin accessibility was altered, we carried out ATAC-seq in E13.5 cortex. Analysis of genome-wide ATAC-seq peaks^46^ (arrows in IGV^47^, Supplementary Fig. 5h) revealed a 91.3% overlap between *Ino80* cKO-E and control. Chromatin accessibility at TSSs was similar and peak-to-peak correlation of reads per peak was high (*R*^2^ = 0.88, Supplementary Fig. 5i, j), with a modest reduction in accessibility in cKO-E. To assess correlation between accessibility and transcriptomic changes in cKO-E, we identified differentially accessible regions (DARs) using GFOLD^48^. Consistent with modest changes, 0.75% of ATAC-seq peaks met GFOLD cutoff of 1.0 for increased accessibility, and 1.02% of peaks for decreased accessibility (Supplementary Fig. 5k). We identified ATAC-seq peaks within predicted E14.5 brain enhancers (EnhancerAtlas 2.0^49^) or at TSSs of genes that showed differential gene expression (DEX) in cKO-E compared with ctrl. Intersectional analysis revealed that 0.78% (13/1658) of brain enhancers and 0.24% (2/832) of TSSs of DEX genes contained DARs. Thus, DARs did not correlate with DEX in *Ino80* cKO-E. Together, these data are consistent with previous work that *Ino80* can regulate HR independently of chromatin accessibility^39,40,43^.

### Phenotypic severity correlates with symmetric NPC divisions

**Fig. 6:**
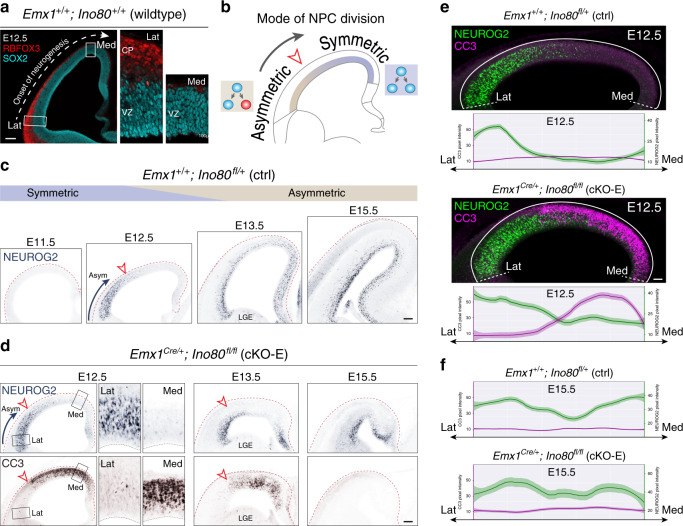
Spatiotemporal correlation of *Ino80* cKO-E apoptosis with mode of NPC division. **a** At the onset of cortical neurogenesis, the mode of NPC division transitions from symmetric NPC–NPC to asymmetric neurogenic in a lateral–rostral first, medial–caudal last manner along the transverse neurogenetic gradient (TNG). In wild-type E12.5 coronal section, lateral neocortex was characterized by a cortical plate (CP) formed by several rows of RBFOX3 + (red) postmitotic neurons, indicating that the SOX2 + (cyan) NPCs in lateral VZ had transitioned to asymmetric neurogenic divisions. In medial neocortex, the CP was not developed, indicating that medial NPCs had not initiated asymmetric neurogenic divisions (*n* = 3 animals). **b** The progression of the TNG is illustrated in schematic. **c** Neurogenic NPC marker NEUROG2 immunostaining (blue) of ctrl coronal sections. At E11.5, NPCs were largely NEUROG2-negative and divided symmetrically. At E12.5, NEUROG2 staining was present in lateral, but not medial, NPCs, consistent with progression of the TNG. At E13.5, neurogenesis had progressed more medially, and by E15.5, neurogenic NPCs were present throughout the mediolateral extent of the neocortex (*n* = 3 animals). **d** NEUROG2 (blue) and CC3 (brown) immunostaining of cKO-E coronal sections. In E12.5 and E13.5 cKO-E cortex, lateral NPCs underwent asymmetric (Asym) neurogenic divisions (NEUROG2+) and were not apoptotic (CC3–), whereas medial NPCs underwent symmetric divisions (NEUROG2–) and were robustly apoptotic (CC3+). At E15.5, when NPCs had largely transitioned to asymmetric neurogenic divisions (NEUROG2+), they no longer underwent apoptosis (*n* = 3 animals). **e** Analysis of immunofluorescent pixel intensity from lateral (Lat) to medial (Med) E12.5 cortex revealed complementary gradients of asymmetric neurogenic divisions (NEUROG2, green) and apoptosis (CC3, magenta) in cKO-E (data are LOESS curve ± 99% confidence interval, *n* = 3 animals). **f** Analysis of immunofluorescent pixel intensity revealed asymmetric neurogenic divisions throughout the mediolateral extent of the E15.5 cortex, and no additional apoptosis in cKO-E (data are LOESS curve ± 99% confidence interval, *n* = 3 animals). Scale bar: 100 μm in **a**, **c**, **d**; 50 μm in **e**.

Our analyses revealed a markedly higher sensitivity of medial cortex to *Ino80* deletion that was not a result of nonuniform Cre activity or *Ino80* expression (Fig. 2e, f), implicating an alternative explanation. During normal corticogenesis, NPCs transition from symmetric NPC–NPC divisions to asymmetric neurogenic divisions^10^ from approximately E11.5 to E13.5. This transition does not occur simultaneously throughout the cortex^50^. Lateral NPCs transition to asymmetric divisions earlier than medial cortex following a transverse neurogenetic gradient (TNG, lateral–rostral → medial–caudal)^50,51^. Consistent with the TNG, our analysis of wild-type E12.5 revealed in lateral cortex a more developmentally advanced cortical plate (CP) comprising rows of RBFOX3 + neurons (red, Fig. 6a), suggesting that lateral SOX2 + NPCs (cyan) have initiated asymmetric division and neurogenesis. In contrast, the medial CP was not developed and did not contain RBFOX3 + neurons, suggesting that medial NPCs were largely dividing symmetrically (schematic, Fig. 6b). We also visualized the TNG by immunostaining for NEUROG2 (NGN2), a pro-neural gene expressed by NPCs undergoing neurogenic divisions^52^. In E11.5 wild-type cortex, a large majority of NPCs were NEUROG2-negative (Fig. 6c), consistent with largely symmetric divisions at this age. From E12.5 to E15.5, wild-type cortex was characterized by an increase in the number of NEUROG2 + NPCs following a lateral to medial gradient. At E12.5, a transition zone delineated NEUROG2-positive and NEUROG2-negative NPCs along the mediolateral axis (arrowhead, Fig. 6c). By E15.5, the entire mediolateral extent showed high NEUROG2 expression, consistent with asymmetric divisions at this age.

We examined the possibility that this spatiotemporal gradient of neurogenesis contributed to the mediolateral difference in NPC sensitivity to *Ino80* deletion. Strikingly, at E12.5 and E13.5, the border of medial apoptotic and lateral nonapoptotic cells in cKO-E aligned with the NEUROG2+/NEUROG2– transition zone (red arrowheads, Fig. 6d). Laterally, where NPCs have initiated asymmetric division, CC3 immunostaining was largely absent (inset, Fig. 6d). In contrast, medially, where NPCs were largely dividing symmetrically, CC3 immunostaining was abundant, suggesting that the symmetric mode of division was associated with NPC sensitivity to *Ino80* deletion. Analysis of immunofluorescent pixel intensity from lateral to medial neocortex revealed strikingly complementary, opposing gradients of NEUROG2 (green) versus CC3 (magenta) in cKO-E (Fig. 6e). To determine whether apoptosis persisted through transition into asymmetric divisions, we analyzed cKO-E cortex at E15.5, when both medial and lateral NPCs have largely transitioned to asymmetric divisions. Remarkably, cKO-E showed no persistent apoptosis at E15.5 (Fig. 6d, f), suggesting that the remaining NPCs, which continued to cycle (Supplementary Fig. 2a), were no longer sensitive to *Ino80* deletion. Together, these data supported a spatiotemporal correlation between NPC sensitivity to *Ino80* deletion and mode of NPC division. Near the onset of *Ino80* deletion in cKO-E, the severely affected medial NPCs were largely undergoing symmetric NPC–NPC divisions, whereas the unaffected lateral NPCs have initiated transition to asymmetric neurogenic divisions. By mid-neurogenesis, NPCs have largely transitioned to asymmetric divisions and no longer underwent apoptosis in cKO-E.

### *Ino80* function in symmetric versus asymmetric NPC divisions

**Fig. 7:**
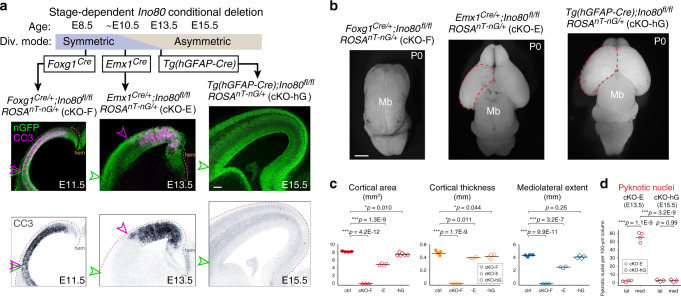
Systematic deletion of *Ino80* from NPCs pre-, peri-, and post transition to asymmetric division. **a** *Ino80* was systematically deleted from NPCs pre-, peri-, and post transition from symmetric to asymmetric division (Div.). Cre-dependent nGFP reporter (green) was expressed from *ROSA*^*nT-nG*^. *Foxg1*^*Cre*^ deletion of *Ino80* (cKO-F) during exclusively symmetric NPC–NPC divisions led to widespread CC3 staining (magenta in overlay, blue in monochrome) throughout the mediolateral axis of E11.5 cortex. The lateral extent of apoptosis (magenta arrowhead) reached the lateral extent of the neocortex (green arrowhead). *Emx1*^*Cre*^ deletion of *Ino80* (cKO-E) near the onset of transition between symmetric and asymmetric divisions led to robust apoptosis in medial, but not lateral, neocortex. Tg*(hGFAP-Cre)* deletion of *Ino80* (cKO-hG) after most NPCs transitioned to asymmetric neurogenic division did not lead to an increase in apoptosis (*n* = 3 animals). **b** Dorsal view of P0 whole-mount brains. cKO-F was characterized by forebrain agenesis. cKO-E exhibited microcephaly and medial defects in corticogenesis. cKO-hG brains were similar to ctrl in size and morphology (cKO-F: *n* = 3, cKO-E: *n* = 4, cKO-hG: *n* = 6). **c** Cortical area (red), thickness (yellow), and mediolateral extent (blue) of P0 cKO-F, cKO-E, and cKO-hG compared with ctrl (data are mean, one-way ANOVA with Tukey’s post hoc test, ctrl: *n* = 4, cKO-F: *n* = 3, cKO-E cortical area: *n* = 4, cKO-E thickness and mediolateral extent: *n* = 3, cKO-hG cortical area: *n* = 6, cKO-hG thickness and mediolateral extent: *n* = 4 animals). **d** Pyknosis was significantly increased in medial E13.5 cKO-E cortex, but not in E15.5 cKO-hG cortex (data are mean, one-way ANOVA with Tukey’s post hoc test, cKO-E: *n* = 4, cKO-hG: *n* = 3 animals). Scale bar: 100 μm in **a**; 1 mm in **b**.

To directly test the possibility that division symmetry contributed to medial NPC sensitivity following *Ino80* loss, we systematically compared *Ino80* deletion from NPCs pre-, peri-, and post transition from symmetric to asymmetric division. To delete *Ino80* pre-transition, we used *Foxg1*^*Cre*^ (*Foxg1*^*Cre/+*^;*Ino80*^*fl/fl*^, cKO-F), which mediates deletion from forebrain NPCs starting at E8.5^53^, an early stage when NPC divisions were exclusively symmetric NPC–NPC. Remarkably, *Foxg1*^*Cre*^ deletion of *Ino80* led to widespread apoptosis throughout the entire mediolateral extent of cKO-F cortex at E11.5 (Fig. 7a). Therefore, at a stage when all NPCs were undergoing symmetric divisions, medial and lateral NPCs were equally sensitive to *Ino80* deletion. This manifested postnatally as forebrain agenesis (Fig. 7b, c). Immunostaining revealed p53 activation throughout the mediolateral axis of E11.5 cKO-F cortex (Supplementary Fig. 7a), indicating DNA damage in both medial and lateral NPCs. We further generated *Foxg1*^*Cre/+*^;*Ino80*^*fl/fl*^*;Trp53*^*fl/fl*^ (dKO-F), which was characterized by a near complete rescue of the forebrain agenesis phenotype of cKO-F (Supplementary Fig. 7c). Thus, the phenotypes of cKO-F, similar to those of cKO-E, were also underpinned by DNA damage and p53 activation. Furthermore, lateral NPCs, during exclusively symmetric divisions, were equally sensitive to loss of *Ino80* as medial NPCs. Together, these data suggested that the spatiotemporal difference in phenotypic severity in the *Emx1*^*Cre*^ cKO-E (peri-transition) was underpinned by a mediolateral difference in mode of NPC division.

To assess the consequence of *Ino80* deletion post transition to asymmetric division, we generated *Ino80* cKO using Tg(*hGFAP-Cre*)^54^ (Tg(*hGFAP-Cre*);*Ino80*^*fl/fl*^, cKO-hG). Tg(*hGFAP-Cre*) mediates deletion in cortical NPCs beginning at E12.5 and extending throughout the cortex at E13.5, a stage when NPCs transition to asymmetric neurogenic divisions. Remarkably, the E15.5 cKO-hG cortex showed no increase in apoptosis compared with ctrl (Fig. 7a, d) and did not activate p53 (Supplementary Fig. 7b). At P0, cKO-hG cortex was similar to ctrl in size and morphology (Fig. 7b, c). Thus, after transition to asymmetric divisions, NPCs were no longer sensitive to *Ino80* deletion. Together, our systematic analysis of *Ino80* deletion from NPCs pre-, peri-, and post transition from symmetric to asymmetric division convergently supported that distinct modes of NPC division have divergent requirements for *Ino80*-mediated HR.

Despite impaired HR DNA repair, *Ino80* deletion did not significantly disrupt asymmetrically dividing NPCs, suggesting usage of alternative DSB repair pathways. To test this, we used the in vivo DSB repair pathway choice assay to compare, in control animals, symmetric (E12.5) versus asymmetric (E15.5) NPC divisions. We found that under wild-type conditions, HR could occur after NPC transition to asymmetric divisions, but at a significantly reduced frequency (Supplementary Fig. 7d). Thus, the balance of DSB repair pathways used by NPCs was not constant throughout development; HR became less frequent relative to NHEJ as NPCs transitioned to asymmetric divisions.

### Deletion of HR gene *Brca2* phenocopies *Ino80* deletion

To determine the extent to which *Ino80* cKO phenotypes were based on impaired HR, we orthogonally disrupted HR by conditional deletion of *Brca2*^55^. BRCA2 is required for HR DNA repair, where it mediates the switch on ssDNA from RPA to recombinase RAD51^56^. It is not required for NHEJ^57^. We reasoned that if *Ino80* deletion phenotypes were consequences of disrupted HR, they should be extensively phenocopied following deletion of *Brca2*, a bona fide HR gene.

**Fig. 8:**
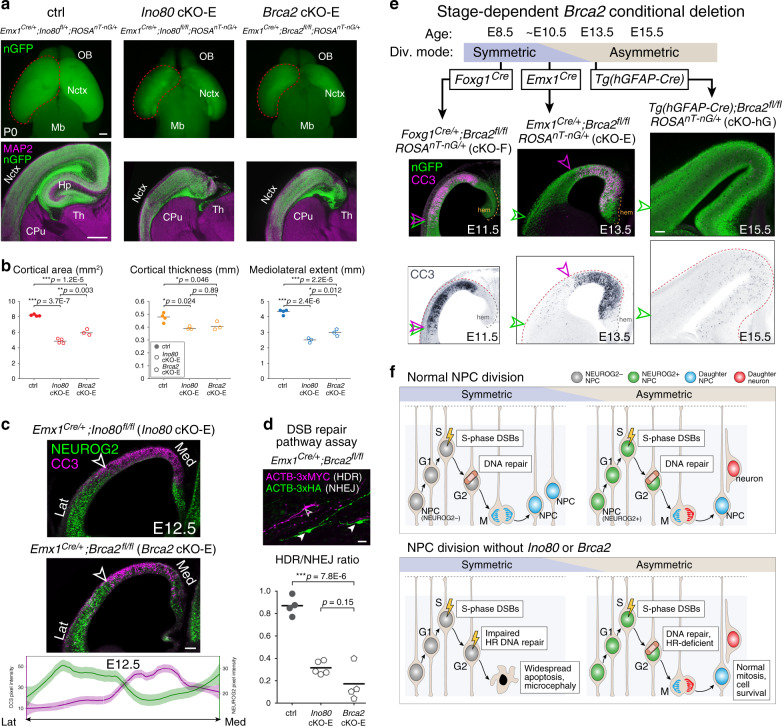
A selective requirement for *Ino80*-mediated HR in symmetric NPC–NPC divisions. **a** P0 whole-mount brains and immunostaining for MAP2 (magenta) and nGFP (green). *Emx1*^*Cre*^-mediated deletion of HR gene *Brca2* (*Brca2* cKO-E) led to microcephaly and hippocampal hypoplasia reminiscent of *Ino80* cKO-E (*n* = 3 animals). **b** The significant reductions in *Ino80* cKO-E cortical area (red), thickness (yellow), and mediolateral extent (blue) were each phenocopied in *Brca2* cKO-E (data are mean, one-way ANOVA with Tukey’s post hoc test, ctrl: *n* = 4, *Ino80* cKO-E cortical area: *n* = 4, thickness, mediolateral extent: *n* = 3, *Brca2* cKO-E: *n* = 3 animals). **c** Complementary gradients of asymmetric neurogenic divisions (NEUROG2, green) and apoptosis (CC3, magenta) in *Brca2* cKO-E E12.5 cortex similar to those found in *Ino80* cKO-E (data are LOESS curve ± 99% confidence interval, *n* = 3 animals). **d** In vivo DSB repair pathway assay revealed significant decrease in HDR (ACTB-3xMYC, magenta) relative to NHEJ (ACTB-3xHA, green) in *Brca2* cKO-E compared with ctrl (data are mean, one-way ANOVA with Tukey’s post hoc test, ctrl: *n* = 4, *Ino80* cKO-E: *n* = 5, *Brca2* cKO-E: *n* = 4 animals). **e** *Foxg1*^*Cre*^ deletion of *Brca2* (cKO-F) during exclusively symmetric NPC–NPC divisions led to widespread CC3 (magenta in overlay, blue in monochrome) throughout the mediolateral axis of E11.5 cortex. The lateral extent of apoptosis (magenta arrowhead) reached the lateral extent of neocortex (green arrowhead). *Emx1*^*Cre*^ deletion of *Brca2* (cKO-E) near the onset of transition between symmetric and asymmetric divisions led to robust apoptosis in medial, but not lateral, neocortex. Tg*(hGFAP-Cre)* deletion of *Brca2* (cKO-hG) after most NPCs had transitioned to asymmetric neurogenic division did not lead to widespread increase in apoptosis. These Cre-by-Cre phenotypes were reminiscent of those found in *Ino80* cKOs (*n* = 3 animals). **f** During corticogenesis, synthesis-associated DSBs in dividing NPCs are repaired in S, G2, or M phase to safeguard genome integrity. Following *Ino80* or *Brca2* deletion from NPCs, HR was selectively disrupted. In symmetrically dividing NPCs, loss of HR led to unrepaired DSBs and apoptosis. In asymmetrically dividing NPCs, *Ino80* or *Brca2* deletion did not give rise to unrepaired DSBs or apoptosis, suggesting that homology-independent DNA repair pathways were sufficient. Thus, distinct modes of NPC division have divergent requirements for HR DNA repair. Scale bar: 500 μm in **a**; 100 μm in **c**, **e**; 20 μm in **d**.

Analyzed at P0, *Brca2* deletion by *Emx1*^*Cre*^ (*Emx1*^*Cre/+*^;*Brca2*^*fl/fl*^, *Brca2* cKO-E) led to significant microcephaly (Fig. 8a, b), callosal agenesis, and hippocampal hypoplasia (Supplementary Fig. 8a, b) remarkably similar to *Ino80* cKO-E. Quantification of layer markers revealed significant changes in medial, but not lateral, *Brca2* cKO-E cortex (Supplementary Fig. 8c). Cortical lamination was also selectively disrupted in medial *Brca2* cKO-E cortex, although less severely compared with *Ino80* cKO-E. Thus, the neonatal phenotypes of *Ino80* cKO-E were largely recapitulated in *Brca2* cKO-E.

At E12.5, the *Brca2* cKO-E cortex was characterized by apoptosis in medial cortex, where NPCs were largely NEUROG2-negative, but not lateral cortex, where NPCs were largely NEUROG2-positive (Fig. 8c). These mediolateral gradients are strikingly reminiscent of *Ino80* cKO-E (Fig. 6). Furthermore, at E13.5, we found preferential pyknosis and accumulation of pKAP1-labeled DSBs in medial *Brca2* cKO-E cortex (Supplementary Fig. 8d, e). Compared with *Ino80* cKO-E, *Brca2* lateral cortex showed a modest increase in apoptosis (Supplementary Fig. 8d, e). *Brca2* has been shown to also play a role in chromosome segregation^58^, defects in which it may contribute to the mild cell death in lateral NPCs. Overall, the mediolateral patterns in phenotypic severity of *Ino80* cKO-E (Figs. 1 and 2) were extensively phenocopied in *Brca2* cKO-E.

Analysis of DSB repair pathway choice showed in *Ino80* cKO-E a preferential loss of HR (Fig. 4). To assess whether the phenocopy between *Ino80* deletion and *Brca2* deletion was mechanistically centered on disrupted HR, we used the DSB repair pathway assay. This showed a relative decrease in HDR compared with NHEJ in *Brca2* cKO-E highly reminiscent of *Ino80* cKO-E (Fig. 8d). These data thus validated that the DSB repair pathway assay could measure impaired HR DNA repair, and implicated disrupted HR as a shared molecular mechanism underlying the mediolateral phenocopy between *Ino80* cKO-E and *Brca2* cKO-E. Together, these data strongly support that the mediolateral differences in phenotypic severity in *Ino80* cKO-E were mechanistically based on disrupted HR DSB repair.

*Ino80* deletion pre-, peri-, and post NPC transition from symmetric to asymmetric division revealed divergent requirements for *Ino80*-mediated HR during distinct modes of NPC division (Fig. 7). To systematically dissect the consequences of *Brca2* deletion, we similarly used *Foxg1*^*Cre*^, *Emx1*^*Cre*^, and Tg(*hGFAP-Cre*). We found a remarkable Cre-by-Cre phenocopy between *Ino80* and *Brca2* cKOs. *Brca2* deletion by *Foxg1*^*Cre*^ during exclusively symmetric divisions led to widespread apoptosis throughout the mediolateral extent of E11.5 cortex (Fig. 8e), whereas *Brca2* deletion by Tg(*hGFAP-Cre*), after NPC transition to asymmetric divisions, did not. This striking Cre-by-Cre phenocopy between *Ino80* and *Brca2*, a bona fide HR gene, provided strong support that *Ino80* mediates DNA repair by HR during corticogenesis, and that NPC requirement for HR DNA repair is dependent on division mode—symmetric NPC–NPC divisions, but not asymmetric NPC-neuron divisions, are highly sensitive to loss of HR.

## Discussion

Biological processes on nuclear DNA occur in the context of chromatin. Replication, repair, and transcription are thus mediated by remodelers that control chromatin mobility and dynamics. Here, we find mechanistically distinct roles for chromatin remodeler *Ino80* in HR DNA repair and YY1-associated transcription in corticogenesis. Following *Ino80* deletion from NPCs, DNA repair by HR is selectively impaired, leading to an accumulation of DSBs, p53 activation, apoptosis, and microcephaly. Co-deletion of *Trp53* with *Ino80* led to extensive phenotypic rescue, similar to other mutants with DNA damage^30,59–61^. Our data thus strongly support *Ino80* cKO apoptosis and microcephaly as consequences of impaired DNA repair.

In addition to DNA repair, we find a gene regulatory role for *Ino80* in YY1-associated transcription. Previous studies have shown that INO80 and YY1 interact^44^, bind open chromatin^15^, and mediate transcriptional activation^17^. This role is compromised in *Ino80* cKO-E, which is characterized by downregulation of YY1-associated genes. These transcriptomic changes persisted in *Ino80*/*Trp53* dKO-E, in which we found no overt neuroanatomical abnormalities (Fig. 5). Thus, YY1-associated expression changes did not contribute to structural phenotypes in *Ino80* cKO-E. Together, these findings support the conclusion that *Ino80* plays mechanistically distinct roles in YY1-associated gene regulation and HR DNA repair in corticogenesis (Supplementary Fig. 6).

Recent genetic findings have converged on altered chromatin regulation in disorders of brain development, including autism spectrum disorder (ASD) and intellectual disability. Unraveling the functional consequences of these mutations is an active area of research. Chromatin dysregulation perturbs transcriptional control^3,4^, but can also impair DNA repair^5,6^. Our findings support compromised HR DSB repair as a likely mechanism of *INO80* patient microcephaly^20^, and highlight the possibility that disruption of chromatin-mediated DNA repair can contribute to neurodevelopmental disorders. Interestingly, other chromatin remodelers associated with brain disorders have been shown to play a role in DNA repair outside of brain development. *CHD2* (childhood-onset epileptic encephalopathy) is recruited by PARP1 to sites of DNA damage in HEK293 and U2OS cells^62^. *ARID1B* (Coffin–Siris syndrome and ASD) mediates NHEJ in cancer cells^63^. Whether potential DNA repair functions of these or other chromatin regulators contribute to neurodevelopment or disorders thereof remains to be fully explored. A predicted consequence of compromised DNA repair is somatic mutations. Emerging studies support a contribution of brain somatic mutations to disorders including epilepsy and cortical dysplasia^14^. It is possible that chromatin dysregulation can increase post-zygotic mutations via compromised DNA repair. Thus, germline mutations in chromatin genes may contribute to brain disorders by disrupting brain genomes.

DNA damage inevitably arises during genome replication. In human cells, the genome sustains ~50 DSBs per doubling^37^. During S phase, DSBs can arise from conflicts between replication and transcription^64–66^. DNA repair is therefore essential to dividing cells, including NPCs^7,8^. Here, using an in utero DNA repair assay, we assess DSB repair pathways in NPCs in vivo (Fig. 4c). The ability of our assay to detect loss of HR is validated by *Brca2* deletion, which selectively impairs HR without affecting NHEJ^57^. We find a reduction in HDR following *Ino80* deletion comparable to that which followed *Brca2* deletion (Fig. 8d), supporting an *Ino80* role in HR in NPCs. We note as a potential caveat that a theoretical reduction in NHEJ may not be detectable by our assay. Loss of HR in *Ino80* cKO-E, however, is consistent with INO80 function in mobilizing H2A.Z and exchanging RPA for RAD51 on resected DNA, key steps in HR^39,40^. In S or G2 phase, with the presence of a sister chromatid as template, HR is well positioned to repair replication-associated DSBs^16^. Consistent with this, cycling NPCs undergo apoptosis following deletion of HR genes in a p53-dependent manner^13,61^ similar to *Ino80* cKO.

Surprisingly, sensitivity to loss of *Ino80*-mediated HR is not universal. Our analysis of *Ino80* cKO-E revealed unrepaired DSBs, p53 activation, and apoptosis in medial, but not lateral, cortical NPCs. During normal corticogenesis, NPCs first undergo symmetric divisions, then transition to asymmetric divisions^10^. This transition is characterized by the TNG (Fig. 6a, b), in which lateral NPCs transition to asymmetric divisions before medial NPCs^50,51^. In *Ino80* cKO-E, phenotypic severity shows a remarkable complementarity to the TNG (Fig. 6e). At the onset of *Ino80* deletion by *Emx1*^*Cre*^, lateral NPCs have initiated transition to asymmetric divisions; they are spared from DNA damage and cell death. Medial NPCs largely remain in symmetric divisions at this age; they are affected by unrepaired DSBs and apoptosis. These data support a sensitivity to *Ino80* deletion in symmetrically dividing NPCs that underpins the mediolateral phenotypes in *Ino80* cKO-E, in which NPC division mode is coupled to NPC spatial positioning at *Emx1*^*Cre*^ onset. To test the selective requirement for *Ino80* by symmetrically dividing NPCs, we leverage additional Cre lines in which NPC division mode is uncoupled from mediolateral position at Cre onset. To study NPCs dividing exclusively in symmetric mode regardless of location, we use *Foxg1*^*Cre*^. Remarkably, *Foxg1*^*Cre*^ deletion of *Ino80* leads to widespread apoptosis throughout the entire mediolateral extent of the cortex (Fig. 7). Thus, during exclusively symmetric divisions, lateral and medial NPCs are equally sensitive to loss of *Ino80*. To study NPCs after both medial and lateral NPCs have initiated transition to asymmetric divisions, we use Tg(*hGFAP-Cre*). Strikingly, Tg(*hGFAP-Cre*) deletion of *Ino80* leads to neither apoptosis, p53 activation, nor microcephaly (Fig. 7). Thus, following NPC transition to asymmetric divisions, neither lateral nor medial NPCs are sensitive to loss of *Ino80*. Together, our systematic analysis of *Ino80* deletion pre-, peri-, and post transition from symmetric divisions to asymmetric divisions revealed a previously unappreciated relationship between division symmetry and DNA repair pathway; symmetric, but not asymmetric, divisions are selectively sensitive to loss of *Ino80*-mediated HR (Fig. 8f). This remarkable division mode-dependent requirement for HR is orthogonally validated in conditional mutants of *Brca2*, a well-characterized HR gene^57^. Notably, early NPCs have been previously shown to be especially sensitive to DNA damage following conditional deletion of *Brca1*^67^ or *Topbp1*^7,60^. Our results suggest that the symmetry of NPC division may contribute to this temporal difference. More broadly, to support organogenesis, stem cells undergo symmetric and asymmetric divisions. Our work thus suggests that stem cell division mode can bias DNA repair pathway choice in other developmental systems.

Symmetric and asymmetric NPC divisions are characterized by remarkable differences in cell cycle dynamics. In symmetric divisions, S-phase length is ~8 h^68^. In contrast, S-phase length is merely 2 h in asymmetric divisions^68^. This difference has key implications for repair of replication-associated DSBs. NHEJ can be completed within 30 min^69^. HR requires extensive DNA processing, recruitment of proteins to resected DNA, and search for a homology template, and thus can take 7 h or longer^69^. Given the accelerated S phase, homology-independent DNA repair may be preferred in asymmetric divisions. Our finding of reduced HR relative to NHEJ as NPCs transition to asymmetric divisions (Supplementary Fig. 7d) is consistent with this possibility. NHEJ is essential to neurodevelopment. *Lig4* and *Xrcc4* mutant mice are characterized by embryonic lethality and neuronal apoptosis^13,59,70,71^. The shortening of S phase in asymmetric division may be necessary to support rapid cortical neurogenesis in mammals. If accelerated replication necessitates homology-independent DNA repair, which is potentially mutagenic, rapid asymmetric NPC division may reflect a compromise between brain somatic genome integrity and rapid production of neurons within a short neurogenic period. A potential consequence of homology-independent DNA repair is structural rearrangements. Recent studies revealed somatic copy number variants in adult human cortex^14^. Thus, rapid replication and homology-independent DNA repair in asymmetric NPC divisions may contribute to genomic diversity in neurons.

## Methods

### Mice

All experiments were carried out in compliance with ethical regulations for animal research. Mouse strains are listed in Supplementary Table 6. Our study protocol was reviewed and approved by the University of Michigan Institutional Animal Care & Use Committee. Mice were maintained on a standard 12-h day:night cycle with ad libitum access to food and water. Timed pregnancies were obtained by using copulation plugs to determine the timing of mating. Genomic DNA was isolated from toe or tail clips, and PCR genotyping was performed using DreamTaq Green 2x Master Mix (Thermo Fisher) with primers listed in Supplementary Table 7.

### Immunostaining

Brains were dissected and fixed in 4% PFA in PBS at 4 °C with agitation overnight. Brains were embedded in 4% low-melting agarose and sectioned using a Leica Vibratome (VT1000S or VT1200S). The primary and secondary antibodies used are listed in Supplementary Tables 8 and 9. As noted in the tables, some antigens required antigen retrieval in pH 6.1 citrate buffer (Dako, Agilent) at 70 °C for 30 min. Sections were then incubated for 1 h at RT in blocking solution consisting of 5% donkey serum, 1% BSA, 0.1% glycine, 0.1% lysine, and 0.3% Triton X-100. Primary antibodies were added to blocking solution and incubated with free-floating sections overnight at 4 °C with agitation. Sections were washed 3 × 5 min in PBS and incubated with fluorescently linked secondary antibodies and DAPI, diluted in blocking solution, for 1 h at RT. Sections were mounted with VECTASHIELD mounting media (Vector Laboratories). For some sections with endogenous fluorescent reporters (nT-nG), 100 mM HCl was added to VECTASHIELD to decrease the pH to ~6 to reduce fluorescence of the reporter fluorescent proteins.

### Western blotting

Mouse cortex was lysed in 1×SDS buffer, boiled at 95 °C for 5–10 min, and run on 4–12% Bis-Tris gel (Invitrogen NP0321). Super sensitive ECL (Thermo, SuperSignal West Pico 34079) was used for signal detection.

### Imaging

Whole-mount and early postnatal brain images were taken on an Olympus SZX16 dissecting scope. Q-Capture Pro 7 software was used to operate a Q-Imaging Retiga 6000 camera. Confocal images were obtained on an Olympus Fluoview FV1000 confocal microscope utilizing Olympus FV10-ASW software.

### EdU fate mapping

EdU was given by intraperitoneal injection at a concentration of 5 µg g^−1^. For EdU staining, sections were permeabilized by incubation for 30 min in 0.5% Triton X-100 in PBS followed by 3 × 5-min washes in PBS. EdU staining solution was made fresh each time, containing 100 mM Tris, 4 mM CuSO_4_, and 100 mM ascorbic acid diluted in 1× PBS. The fluorescently labeled azide molecule was added last, and the staining cocktail was immediately added to sections for a 30-min incubation at room temperature (final concentrations of each fluorescent molecule: 4 mM AlexaFluor488-Azide [Click Chemistry Tools, 1275-1], 10 mM Cy3-Azide [Lumiprobe, A1330], 16 mM AlexaFluor647-Azide [Click Chemistry Tools, 1299-1]). After incubation, sections were washed 3 × 5 min in PBS. After EdU labeling, standard protocols were followed for immunostaining.

### DSB repair assay

A gRNA sequence targeting the c terminus of the *Actb* coding sequence (5′-AGTCCGCCTAGAAGCACTTG) was cloned into eSpCas9Opt1.1, a vector expressing enhanced-specificity Cas9 molecule and an optimized gRNA scaffold. To construct Actb_NHEJ_3xHA, tandem HA tag sequences were cloned between two *Actb* gRNA recognition sequences in the same orientation. This orientation is reversed from that in the genome to prevent recutting of 3xHA inserted in the forward orientation following NHEJ repair. Inversed insertion of 3xHA would reconstitute the gRNA recognition sequences, enabling recutting. Actb_HDR_3xMyc was constructed by cloning tandem Myc tags between 800-bp homology arms^41^. The gRNA target sequence in the HDR-repair template was altered at 7 basepairs to prevent cleavage of the repair template or subsequent recutting after HDR-mediated repair. For IUE, timed-pregnant dams were anesthetized, and a midline incision was made to expose the uterine horns. Ring forceps were used to gently maneuver uterine horns from the body cavity. A pulled micropipette was loaded with plasmid DNA solution: a mix of eSpCas9opt1.1-Actb_gRNA, Actb_NHEJ_3xHA, Actb_HDR_3xMyc, and CAG-Lifeact3xBFP plasmids at 1 µg µl^−1^, with 0.1% Fast Green FCF. The micropipette was inserted into the lateral ventricle, and injection was controlled using a World Precision Instruments Micro4 MicroSyringe Pump Controller. Following injection of the plasmid solution, electroporation paddles were used to deliver 4–5 pulses of 27 volts (45 milliseconds each), with 950 milliseconds between pulses, using a BTX Harvard Apparatus ECM 830 power supply. Brains were analyzed 5 days after electroporation. Following serial sectioning, BFP-positive sections were isolated and immunostained using anti-HA and anti-Myc antibodies. For each electroporated animal, at least 100 positively labeled cells were counted to determine the HDR/NHEJ ratio.

### RNA isolation

Embryos were isolated and immediately submerged in ice-cold PBS. Neocortical tissue was dissected and flash-frozen in a dry ice–ethanol bath and stored at −80° until further processing. Tissue was resuspended in 0.5 mL of Trizol and homogenized using metal beads in a bullet blender. Chloroform was added to the sample, and the aqueous phase was isolated following centrifugation for 15 min at >20,000 *g* at 4 °C. The sample was transferred to a Zymo Research Zymo-Spin IC column and was processed following the manufacturer’s protocol, including on-column DNA digestion. Pure RNA was eluted in DNase/RNase-free water and quantified using a Qubit fluorometer.

### Click-seq

Libraries for RNA-seq were generated from 600 ng of RNA by Click-Seq^29^. Ribosomal RNA was removed from total RNA using NEBNext rRNA Depletion Kit (NEB). ERCC RNA spike-in was included for library quality assessment (Thermo Fisher). Superscript II (Thermo Fisher) was used for reverse transcription with 1:30 5 mM AzdNTP:dNTP and 3′ Genomic Adapter-6N RT primer (GTGACTGGAGTTCAGACGTGTGCTCTTCCGATCTNNNNNN). RNaseH treatment was used to remove RNA template and DNA was purified with DNA Clean and Concentrator Kit (Zymo Research). Azido-terminated cDNA was combined with the click adaptor oligo (/5Hexynyl/NNNNNNNNAGATCGGAAGAGCGTCGTGTAGGGAAAGAGTGTAGATCTCGGTGGTCGCCGTATCATT) and click reaction was catalyzed by addition of ascorbic acid and Cu2 + , with subsequent purification with DNA Clean and Concentrator Kit. Amplification of the library was accomplished using the Illumina universal primer (AATGATACGGCGACCACCGAG) and Illumina indexing primer (CAAGCAGAAGACGGCATACGAGATNNNNNNGTGACTGGAGTTCAGACGTGT) and the manufacturer’s protocols from the 2× One Taq Hot Start Mastermix (NEB). To enrich for amplification products larger than 200 bp, PCR products were purified using Ampure XP (Beckman) magnetic beads at 1.25× ratio. Libraries were analyzed on TapeStation (Agilent) for appropriate quality and distribution and were sequenced at the University of Michigan sequencing core on the Illumina NextSeq 550 platform (75 cycle, high output).

### RNA-seq analysis

RNA-seq data were subject to quality-control check using FastQC v0.11.5 (https://www.bioinformatics.babraham.ac.uk/projects/download.html#fastqc). Adapters were trimmed using cutadapt version 1.13 (http://cutadapt.readthedocs.io/en/stable/guide.html). Processed reads were aligned to the GENCODE GRCm38/mm10 reference genome (https://www.gencodegenes.org/mouse_releases/current.html) with STAR^72^ (v2.5.2a) and deduplicated according to UMI using UMI-tools^73^ (v0.5.3). Read counts were obtained with htseq-count (v0.6.1p1) with intersection–nonempty mode^74^. Differential expression was determined with edgeR^31^. The *P* value was calculated with likelihood ratio tests and the adjusted *P* value for multiple testing was calculated using the Benjamini–Hochberg procedure, which controls false discovery rate (FDR).

### Droplet digital PCR (ddPCR)

In all, 500 ng of RNA was reverse-transcribed to cDNA using Superscript II (Thermo Fisher). Diluted cDNA was used as template for ddPCR analysis. Target gene primer/probe sets (IDT) were labeled with FAM. A HEX-labeled probe for housekeeping gene *Srp72* was used for normalization. Primer and probe sequences are listed in Supplementary Table 10. Reaction mixtures were partitioned using the QX200 droplet generator (Bio-Rad) and subjected to thermocycling. Droplets were analyzed in the QX200 droplet reader (Bio-Rad).

### ATAC-seq

Cortical hemispheres were dissected from embryonic mice at E13.5 and lysed using a dounce homogenizer with 20 strokes of pestle A followed by 20 strokes from pestle B in lysis buffer (10 mM Tris·Cl, pH 7.4, NaCl 10 mM, MgCl_2_ 3 mM, 0.1% v/v NP-40). Nuclei were centrifuged at 500 *g* for 10 min at 4 °C. In total, 50,000 nuclei were isolated, and ATAC-seq was performed using an established protocol. After PCR amplification, libraries were purified with the 1.2 × AMPure Beads. Purified ATACseq libraries were analyzed for quality and nucleosome periodicity using a BioAnalyzer High Sensitivity DNA chip (Agilent Technologies) and quantified using the NEBNext Library Quant Kit for Illumina. Libraries were sequenced on an Illumina NovaSeq S4 flow cell to obtain paired-end 150-bp reads. After trimming adapter using cutadapt^75^, reads were aligned to the mouse reference genome (GRCh38/mm10) using BWA with default settings^76^. Low-quality, mitochondrial, and duplicate reads were removed using a combination of SAMTools and Picard’s MarckDuplicates program. ATAC-seq peaks were called using Macs2 with the parameters: –nomodel –extsize 200 –shift -100, and blacklisted regions were excluded^46^. A consensus set of peaks (137,816) was generated using bedtools merge, and reads from each sample that fell within this consensus peak set were counted using bedtools coverage, and normalized by library size, and to RPM (reads per million). Differentially accessible regions (DARs) were determined using GFOLD^48^ with a significance cutoff score of 1.0. This GFOLD score reflects a confidence interval of >99% that the observed fold change is at least log2(1.0). ATAC-seq peaks were annotated by overlap with TSSs from the UCSC browser (GRCm38). Peaks associated with enhancer regions were determined based on predicted enhancer–gene interaction data from the E14.5 brain dataset from EnhancerAtlas 2.0^49^, which integrates ChIP-seq, DNAse hypersensitivity, ChIA-Pet, Hi-C, and gene expression data. These intersections were established using PyRanges^77^ to find ATAC-seq peaks as called by Macs2 that overlapped with predicted enhancer regions.

### Statistical analysis

Statistical calculations were performed in GraphPad Prism. Values were compared using a two-tailed, unpaired Student’s *t* test or ANOVA with Tukey’s post hoc test. A *P* value of < 0.05 was considered statistically significant.

### Image processing and analysis

Images were exported as TIFF files and processed in Adobe Photoshop. Counts and measurements were performed in Photoshop or using ImageJ software.

### Reporting summary

Further information on research design is available in the Nature Research Reporting Summary linked to this article.

## Supplementary information

Supplementary Information

Reporting Summary

## Data Availability

The RNA-seq and ATAC-seq data that support the findings of this study have been deposited to NCBI GEO with the accession number GSE153062. Data from publicly available sources: GRCm38/mm10 reference genome [https://www.ncbi.nlm.nih.gov/assembly/GCF_000001635.26], ENCODE [ENCSR160IIN, ENCSR647QBV, ENCSR970EWM, ENCSR185LWM, ENCSR752RGN, ENCSR080EVZ, ENCSR362AIZ], and EnhancerAtlas 2.0. Source Data are provided with this paper.

## Source data

Source Data
