# Supplementary material for: Symmetric neural progenitor divisions require chromatin-mediated homologous recombination DNA repair by Ino80
Source: Nat Commun. 2020 Jul 31;11:3839. doi: 10.1038/s41467-020-17551-4 (PMC7395731; doi:10.1038/s41467-020-17551-4)
Supplement: Supplementary file 1 — Supplementary Information [file 41467_2020_17551_MOESM1_ESM.pdf]

Supplementary Information

Symmetric neural progenitor divisions require chromatin-mediated homologous recombination DNA repair by *Ino80*

Jason M. Keil[1,2,3], Daniel Z. Doyle[1,2,4], Adel Qalieh[1,2], Mandy M. Lam[1,2], Owen H. Funk[1,2], Yaman Qalieh[1,2], Lei Shi[1,2], Nitesh Mohan[1,2], Alice Sorel[1,2], Kenneth Y. Kwan[1,2,4,*]

1. Michigan Neuroscience Institute (MNI), University of Michigan, Ann Arbor, MI 48109, USA.
2. Department of Human Genetics, University of Michigan, Ann Arbor, MI 48109, USA.
3. Medical Scientist Training Program, University of Michigan, Ann Arbor, MI 48109, USA.
4. Neuroscience Graduate Program, University of Michigan, Ann Arbor, MI 48109, USA.

*Correspondence should be addressed to K.Y.K. (kykwan@umich.edu)

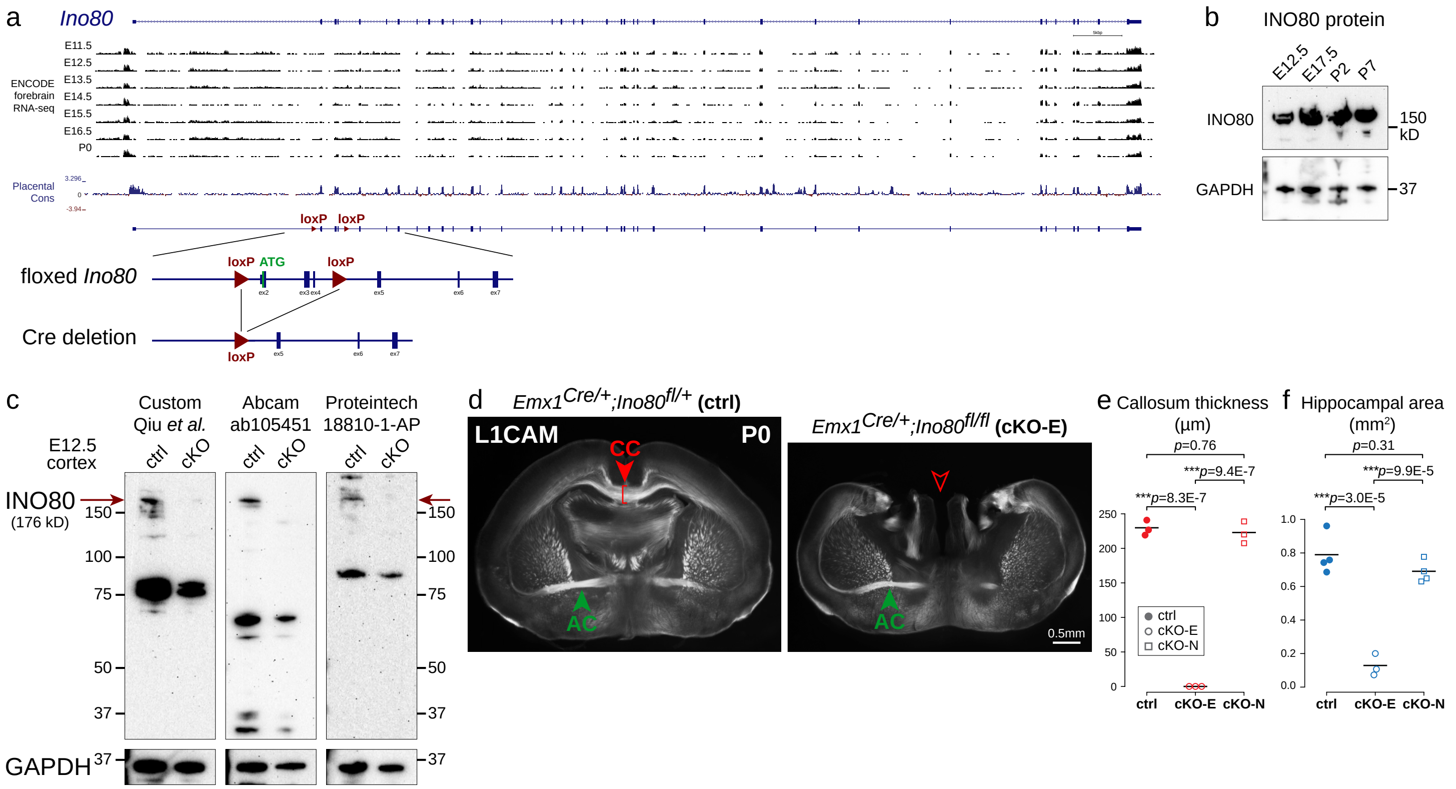

Supplementary Figure 1

a, The mouse *Ino80* locus and the conditional *Ino80* allele. ENCODE RNA-seq data show *Ino80* expression in forebrain from E11.5 to P0. Additional ENCODE RNA-seq data for *Ino80* mRNA expression can be found at https://www.ncbi.nlm.nih.gov/gene/68142/?report=expression. For the conditional *Ino80* allele, the loxP sites (red triangles) flanking exons 2-4 and deletion upon Cre-mediated excision are illustrated. Cons., conservation

b, INO80 immunoblotting of wildtype E12.5, E17.5, P2, and P7 cortical lysate revealed INO80 protein expression throughout cortical developmental ages.

c, INO80 immunoblotting with three anti-INO80 antibodies revealed loss of INO80 protein (~176kD) expression from E12.5 *Ino80* cKO-E cortex.

d, L1CAM immunostaining (white) of coronal P0 sections. In cKO-E, the corpus callosum (CC, red arrowhead) was absent. The anterior commissure (AC, green arrowhead) was formed.

e and **f,** Quantitative analyses of coronal P0 sections revealed significant reductions in corpus callosum thickness (n=3 animals) and hippocampal area (ctrl: n=4, cKO-E: n=3, cKO-N: n=4 animals), in cKO-E, but not cKO-N, compared to ctrl (Data are mean, one-way ANOVA with Tukey's post-hoc test).

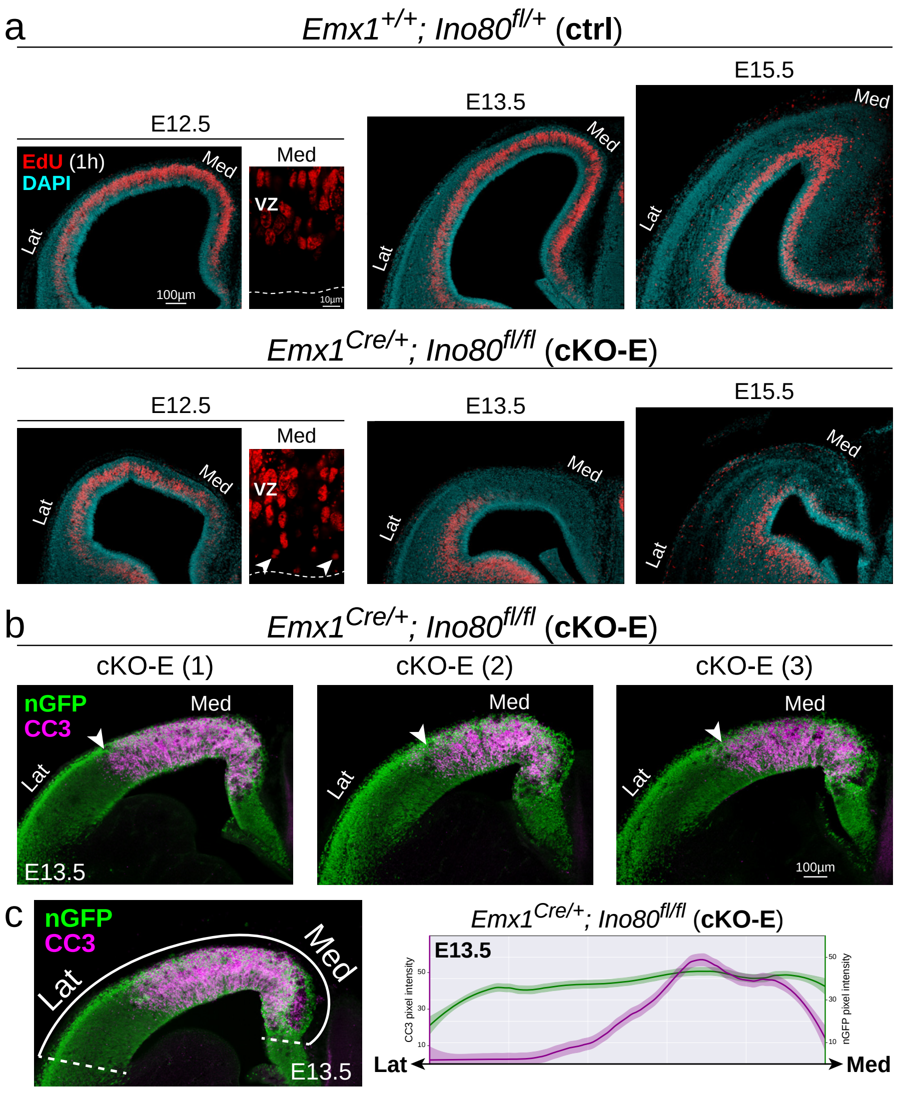

Supplementary Figure 2

a, S-phase NPCs in E12.5, E13.5, and E15.5 brains were labeled by a 1-hour pulse of EdU (red) and coronal sections were stained with DAPI (cyan). From E12.5 to E15.5, the medial (Med) cKO-E cortex was characterized by a progressive loss of EdU+ cycling NPCs. Some EdU+ nuclei in medial cKO-E cortex were pyknotic (arrowheads), suggesting that apoptosis occurred during or within 1 hour of S-phase. In lateral (Lat) cKO-E cortex, EdU+ NPCs were present at E12.5, E13.5, and E15.5, indicating that NPCs continued to proliferate in lateral cortex.

b, Cleaved Caspase 3 (CC3) immunostaining (magenta) of coronal E13.5 sections from three cKO-E brains showed consistent apoptosis in medial, but not lateral, neocortex. The lateral extent of apoptosis (arrowhead) was consistent in all analyzed cKO-E brains. Cre-dependent expression of nGFP (green) from *ROSA*$^{nT\text{-}nG}$ was consistently present in both medial and lateral cKO-E neocortex.

c, Analysis of immunofluorescent pixel intensity from lateral (Lat) to medial (Med) E12.5 cortex revealed Cre-dependent expression of nGFP (green) throughout the mediolateral extent of E13.5 cKO-E neocortex. Apoptosis (CC3, magenta), however, was present only in medial cKO-E cortex (Data are LOESS curve ± 99% confidence interval, n=3 animals).

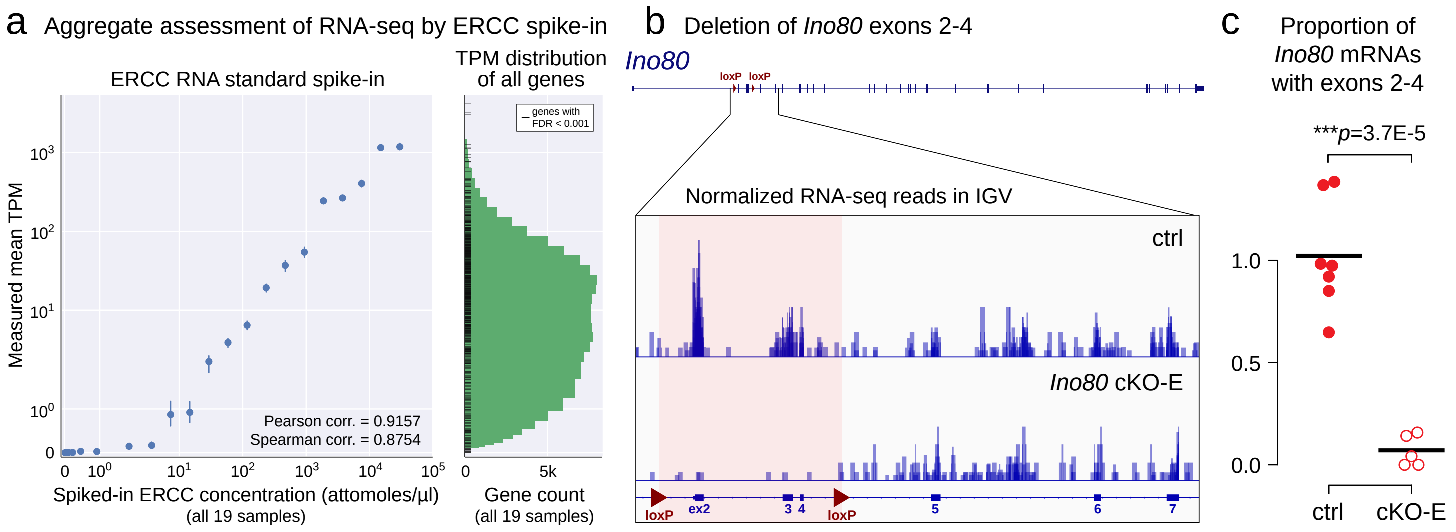

Supplementary Figure 3

a, Aggregate assessment of ERCC spike-in standards in UMI RNA-seq revealed excellent quantification over a broad range of expression values. The vast majority of genes from all 19 experimental samples were within the dynamic range of UMI RNA-seq. TPM, transcripts per million

b, Normalized UMI RNA-seq reads that mapped to *Ino80* exons 2-7 were visualized using Integrative Genomics Viewer (IGV). Loss of RNA-seq reads from exons 2-4 in cKO-E confirmed Cre-mediated deletion of these exons at the level of the transcriptome.

c, The proportion of *Ino80* mRNAs with intact (unrecombined) exons 2-4 based on RNA-seq RPKM in *Ino80* cKO-E and ctrl E13.5 cortex (Data are mean, two-tailed unpaired *t*-test, ctrl: n=7, cKO-E: n=5 animals).

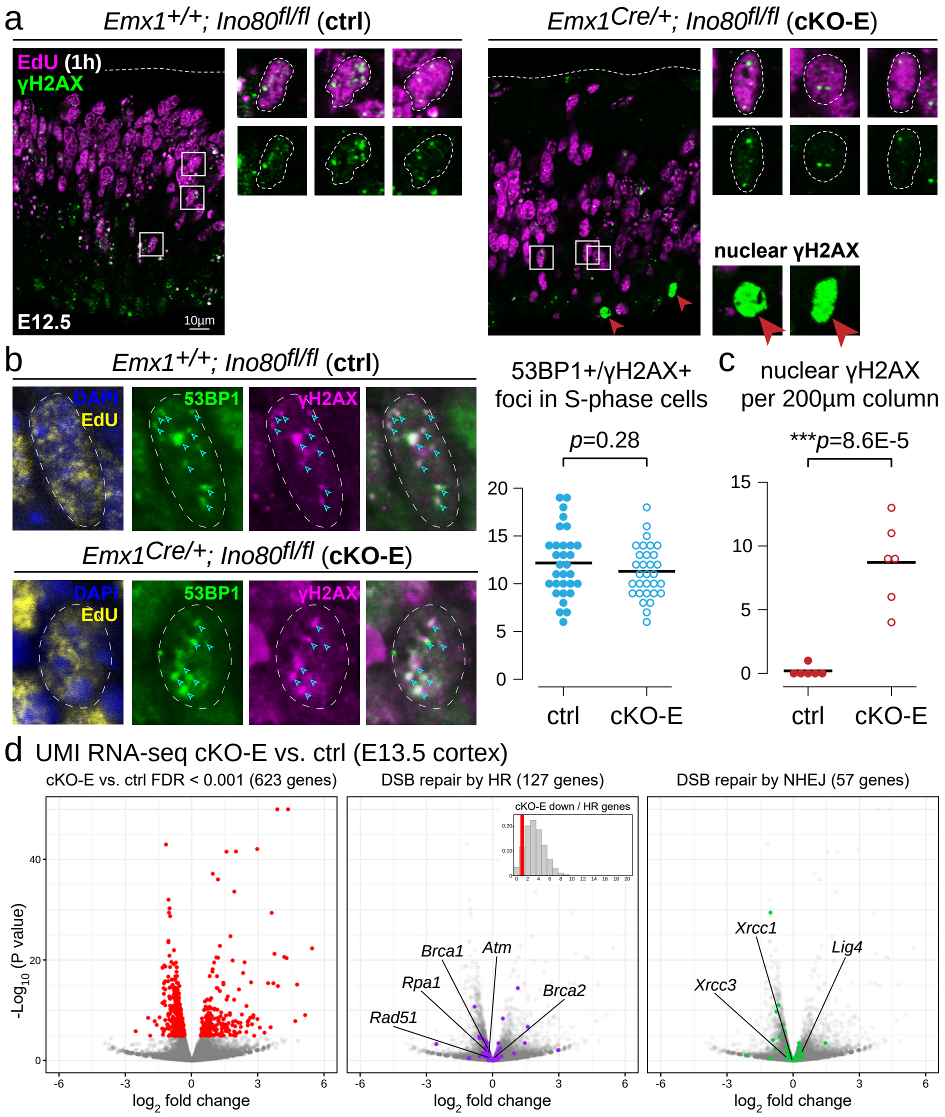

Supplementary Figure 4

a, S-phase NPCs in E12.5 brains were labeled by a 1-hour pulse of EdU (magenta) and coronal sections were immunostained for γH2AX (green). In ctrl, numerous γH2AX foci were present in S-phase NPCs (insets), consistent with physiological DSBs during normal DNA replication. In cKO-E, γH2AX foci were also present in S-phase NPCs. However, in addition to replication-associated DSBs, some cKO-E NPCs near the ventricular surface were characterized by pan-nuclear γH2AX staining (arrowheads), a marker of apoptosis following substantial DNA damage during replication.

b, S-phase DSBs were quantified by co-immunostaining for γH2AX (magenta) and the DNA repair protein 53BP1 (green), which colocalize at sites of DSBs. The number of γH2AX+/53BP1+ foci (arrowheads) in S-phase NPCs (1h EdU+, yellow) was not significantly different in E12.5 *Ino80* cKO-E cortex compared to ctrl (Data are mean, two-tailed unpaired *t*-test, n=31 EdU+ cells from 3 animals).

c, Quantification of pan-nuclear γH2AX staining, a marker of apoptosis following substantial DNA damage during replication, in EdU- ventricular NPCs (arrowheads in **a**) revealed a significant increase in E12.5 cKO-E cortex compared to ctrl (Data are mean, two-tailed unpaired *t*-test, n=6 hemispheres from 3 animals).

d, Expression of DNA damage repair genes in *Ino80* cKO-E. As a group, DNA damage repair genes were not significantly downregulated in cKO-E. HR genes *Brca1*, *Brca2*, *Rpa*, *Atm*, and *Rad51* were not downregulated. Only 1 of 127 genes involved in homology-dependent DNA repair was downregulated in cKO-E.

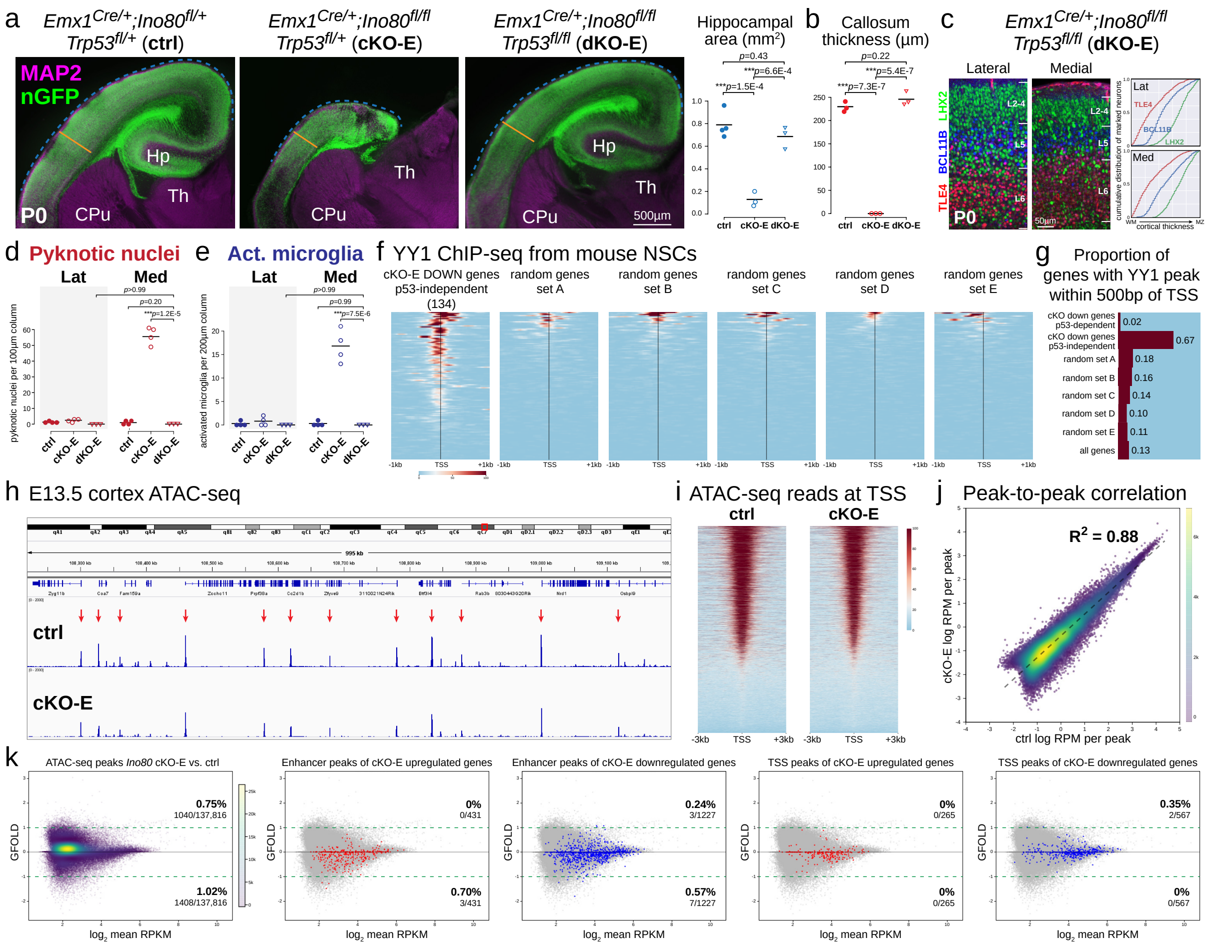

Supplementary Figure 5

a, MAP2 (magenta) and nGFP (green) immunostaining of coronal P0 brain sections. *Ino80* cKO-E, but not *Ino80/Trp53* dKO-E, was characterized by microcephaly and severe hippocampal hypoplasia. Analysis of hippocampal area revealed a significant decrease in cKO-E, but not dKO-E, compared to ctrl (Data are mean, one-way ANOVA with Tukey's post-hoc test, ctrl: n=4, cKO-E: n=3, dKO-E: n=3 animals)

b, Analysis of corpus callosum thickness revealed a significant decrease in P0 cKO-E, but not dKO-E, cortex compared to ctrl (Data are mean, one-way ANOVA with Tukey's post-hoc test, n=3 animals)

c, Analysis of cumulative distribution of layer marker-expressing neurons through the thickness of cortex from WM to MZ revealed normal lamination in medial and lateral P0 dKO-E cortex (n=3 animals).

d and **e**, Quantitative analysis revealed significant increases in pyknosis and morphologically activated (Act.) microglia in medial cKO-E, but not dKO-E, cortex (Data are mean, one-way ANOVA with Tukey's post-hoc test, ctrl: n=4, cKO-E: n=4, dKO-E: n=3 animals).

f and **g,** Intersectional analysis of the 134 p53-independent downregulated genes and five sets of randomly selected genes (sets A-E) with published YY1 ChIP-seq data from mouse neural stem cells (NSCs). The 134 p53-independent, cKO-E downregulated genes showed an enrichment of genes with a YY1 peak within 500bp of transcriptional start site (TSS).

h, Representative IGV tracts of E13.5 cortex ATAC-seq. Analysis of genome-wide ATAC-seq peaks (arrows) revealed a 91.33% overlap between *Ino80* cKO-E and ctrl.

i, Normalized ATAC-seq reads within 3kb of annotated TSSs in *Ino80* cKO-E and ctrl.

j, Genome-wide ATAC-seq peak-to-peak correlation of normalized reads per peak between *Ino80* cKO-E and ctrl.

k, MA plots of chromatin accessibility changes in cKO-E compared to ctrl. Each dot represents one ATAC-seq peak. GFOLD cutoff of 1.0 is indicated by dashed green line.

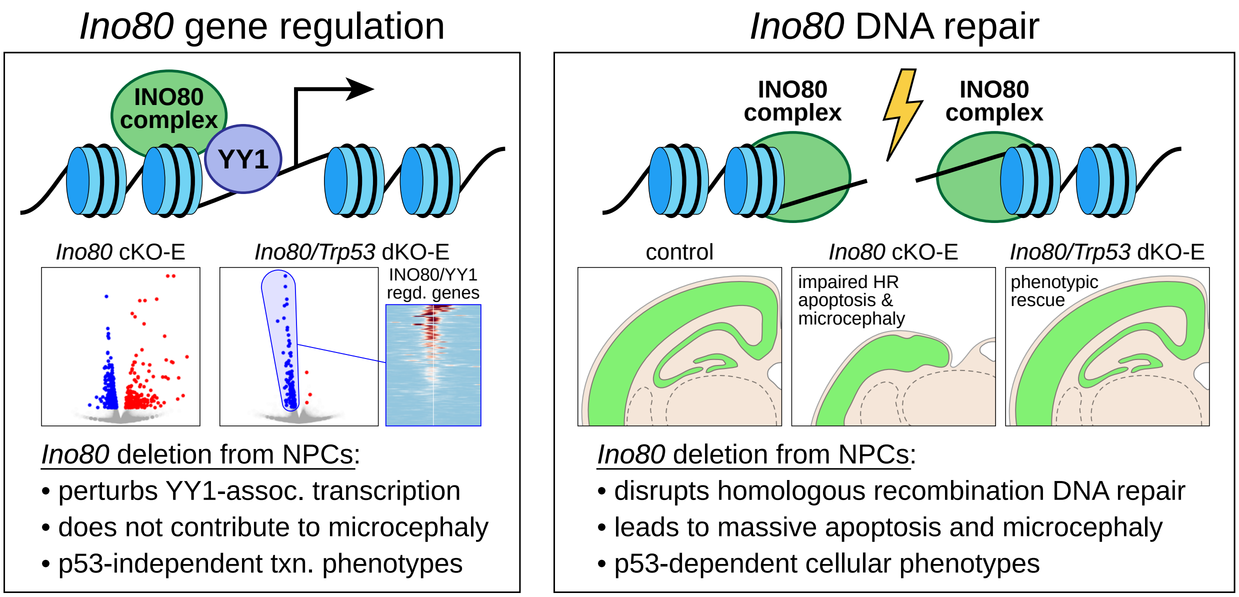

Supplementary Figure 6

The mechanistically distinct roles of *Ino80* in transcriptional regulation and DNA repair during corticogenesis are illustrated in schematic.

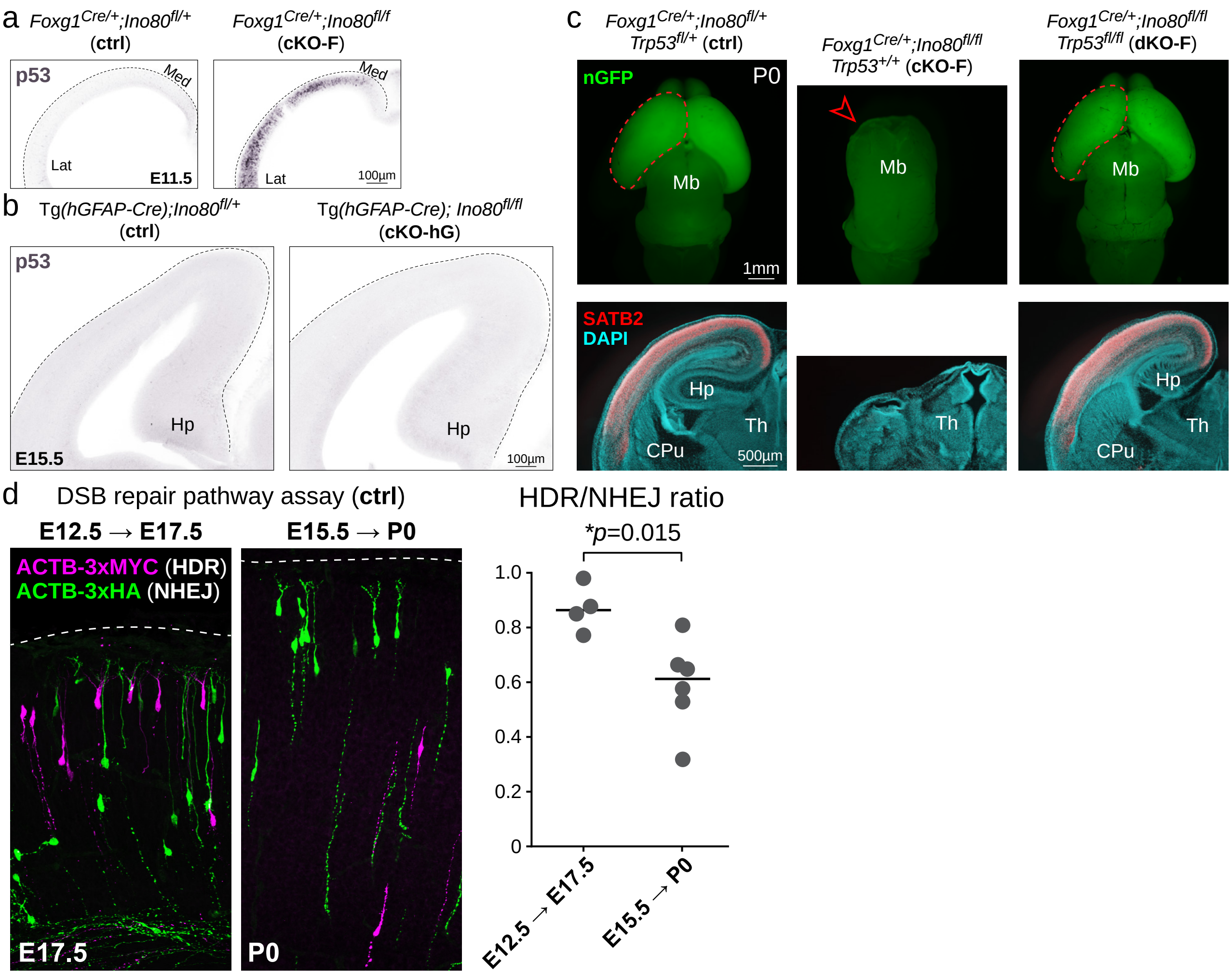

Supplementary Figure 7

a, p53 immunostaining of E11.5 ctrl and *Ino80* cKO-F coronal brain sections. In *Ino80* cKO-F, p53 activation was present in the entire mediolateral extent of the cortex, including lateral neocortex.

b, p53 immunostaining of E15.5 ctrl and *Ino80* cKO-hG coronal brain sections. p53 activation was absent from *Ino80* cKO-hG cortex.

c, Dorsal view of P0 whole mount brains and SATB2 (red) and DAPI (cyan) staining of P0 brain coronal sections. Single deletion of *Ino80* with *Foxg1*^{Cre} (cKO-F) led to forebrain agenesis at P0 (arrowhead). Co-deletion of *Ino80* and *Trp53* with *Foxg1*^{Cre} (dKO-F) led to remarkable rescue of cKO-F forebrain agenesis.

d, *In vivo* DSB repair pathway assay in control brains. IUE was carried out at E12.5, during largely symmetric NPC divisions, or at E15.5, after most NPCs have transitioned to asymmetric divisions. Brains were analyzed 5 days after IUE. After transition to asymmetric divisions, HDR (ACTB-3xMYC, magenta) was significantly decreased relative to NHEJ (ACTB-3xHA, green; data are mean, two-tailed unpaired t-test, E12.5 → E17.5: n=4, E15.5 → P0: n=6 animals).

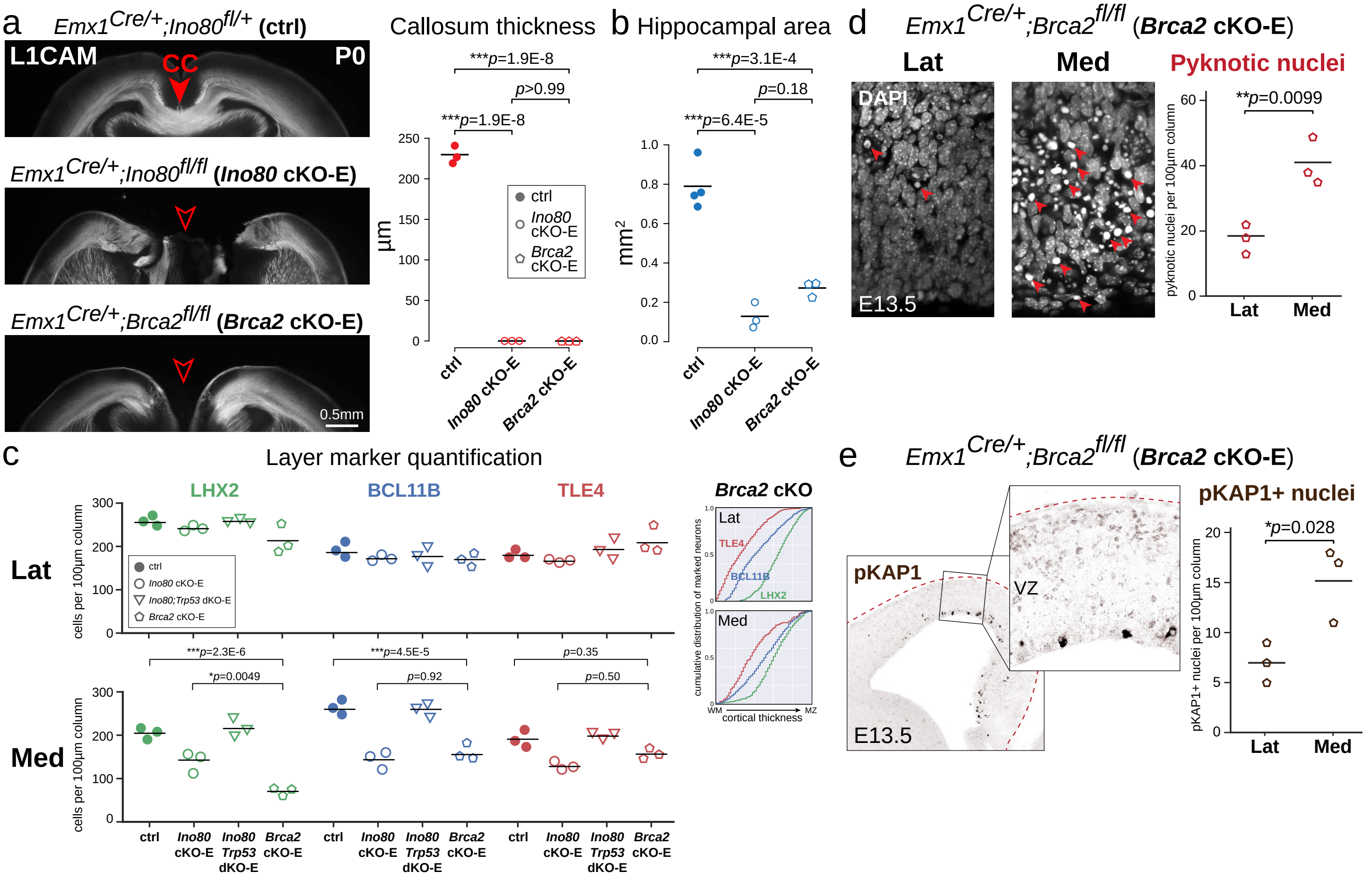

Supplementary Figure 8

a, L1CAM immunostaining (white) of coronal P0 sections. Agenesis of corpus callosum (CC, red arrowhead) was consistently found in *Ino80* cKO-E and *Brca2* cKO-E. Analysis of coronal P0 sections revealed a significant reduction in corpus callosum thickness in *Ino80* cKO-E and *Brca2* cKO-E compared to ctrl (Data are mean, one-way ANOVA with Tukey's post-hoc test, n=3 animals).

b, Analysis of coronal P0 sections revealed a significant reduction in hippocampal area in *Ino80* cKO-E and *Brca2* cKO-E compared to ctrl (Data are mean, one-way ANOVA with Tukey's post-hoc test, ctrl: n=4, *Ino80* cKO-E: n=3, *Brca2* cKO-E: n=3 animals).

c, Analysis of layer marker-expressing neurons in medial (Med) and lateral (Lat) P0 ctrl, *Ino80* cKO-E, *Ino80;Trp53* dKO-E, and *Brca2* cKO-E cortex (Data are mean, one-way ANOVA with Tukey's post-hoc test, n=3 animals).

d, Analysis of pyknotic nuclei (arrowheads) in medial and lateral E13.5 *Brca2* cKO-E cortex (Data are mean, two-tailed unpaired t-test, n=3 animals).

e, Analysis of pKAP1+ nuclei (arrowheads) in medial and lateral E13.5 *Brca2* cKO-E cortex (Data are mean, two-tailed unpaired t-test, n=3 animals).

Supplementary Table 1. Significantly upregulated genes by UMI RNA-seq (cKO-E vs. ctrl, FDR < 0.001)

Ensembl Mouse Gene ID	Gene symbol	logFC	logCPM	LR	PValue	FDR	p53 target	microglia
ENSMUSG00000074968.11	*Ano3*	4.36	4.68	818.46	5.23E-180	2.71E-175		
ENSMUSG00000034457.10	*Eda2r*	3.87	3.20	287.25	1.98E-64	5.14E-60		
ENSMUSG00000021185.16	*Dglucy*	2.96	3.25	188.13	8.13E-43	1.06E-38		
ENSMUSG00000020326.7	*Ccng1*	2.00	4.12	185.89	2.51E-42	2.37E-38		
ENSMUSG00000096914.3	*Galntl6*	1.57	6.28	185.72	2.74E-42	2.37E-38		
ENSMUSG00000039488.15	*Cntn5*	0.95	8.22	165.57	6.86E-38	5.08E-34		
ENSMUSG00000030609.18	*Aen*	1.18	5.32	160.41	9.18E-37	5.96E-33		
ENSMUSG00000048458.8	*Fam212b*	1.93	3.56	149.37	2.38E-34	1.37E-30		
ENSMUSG00000023067.13	*Cdkn1a*	3.62	1.90	130.07	3.95E-30	1.58E-26		
ENSMUSG00000063458.12	*Lrmda*	1.75	3.25	108.81	1.78E-25	6.17E-22		
ENSMUSG00000097039.8	*Pvt1*	1.27	5.97	100.01	1.51E-23	4.36E-20		
ENSMUSG00000024521.7	*Pmaip1*	5.45	1.20	97.81	4.60E-23	1.26E-19		
ENSMUSG00000038776.13	*Ephx1*	3.74	1.46	92.89	5.54E-22	1.37E-18		
ENSMUSG00000004085.14	*Map3k20*	1.11	5.19	89.59	2.93E-21	6.35E-18		
ENSMUSG00000006800.13	*Sulf2*	1.19	4.29	89.30	3.40E-21	7.06E-18		
ENSMUSG00000029304.14	*Spp1*	4.29	1.14	88.89	4.18E-21	8.34E-18		
ENSMUSG00000002985.16	*Apoe*	1.85	3.36	86.81	1.19E-20	2.29E-17		
ENSMUSG00000026249.10	*Serpine2*	2.39	2.34	85.21	2.69E-20	4.81E-17		
ENSMUSG00000057098.14	*Ebf1*	1.01	5.99	84.92	3.10E-20	5.32E-17		
ENSMUSG00000005089.15	*Slc1a2*	0.95	6.17	78.87	6.63E-19	8.82E-16		
ENSMUSG00000066113.16	*Adamtsl1*	0.93	5.55	76.74	1.95E-18	2.53E-15		
ENSMUSG00000028893.8	*Sesn2*	2.33	2.20	75.41	3.83E-18	4.73E-15		
ENSMUSG00000046159.16	*Chrm3*	0.81	5.68	72.59	1.60E-17	1.88E-14		
ENSMUSG00000059857.15	*Ntng1*	1.14	4.29	72.51	1.66E-17	1.92E-14		
ENSMUSG00000041801.5	*Phlda3*	2.68	1.47	67.02	2.69E-16	2.64E-13		
ENSMUSG00000054612.4	*Mgmt*	1.43	3.62	66.62	3.29E-16	3.16E-13		
ENSMUSG00000017724.14	*Etv4*	3.70	0.89	66.24	4.00E-16	3.56E-13		
ENSMUSG00000085912.1	*Trp53cor1*	3.46	1.14	66.28	3.91E-16	3.56E-13		
ENSMUSG00000013089.15	*Etv5*	1.43	4.67	65.16	6.90E-16	5.97E-13		
ENSMUSG00000020053.18	*Igf1*	3.90	0.88	63.72	1.44E-15	1.18E-12		
ENSMUSG00000075324.13	*Fign*	1.13	5.51	61.82	3.76E-15	2.83E-12		
ENSMUSG00000015243.4	*Abca1*	1.16	5.05	61.75	3.91E-15	2.86E-12		
ENSMUSG00000002083.13	*Bbc3*	2.13	2.01	60.67	6.74E-15	4.73E-12		
ENSMUSG00000029212.11	*Gabrb1*	1.27	4.33	59.12	1.48E-14	9.62E-12		
ENSMUSG00000028364.15	*Tnc*	1.63	3.83	54.25	1.77E-13	9.36E-11		
ENSMUSG00000010476.14	*Ebf3*	0.88	5.26	52.79	3.71E-13	1.78E-10		
ENSMUSG00000064293.14	*Cntn4*	1.58	4.37	51.48	7.25E-13	3.30E-10		
ENSMUSG00000039419.17	*Cntnap2*	0.70	7.42	51.49	7.18E-13	3.30E-10		
ENSMUSG00000042286.13	*Stab1*	1.92	2.16	51.35	7.72E-13	3.48E-10		
ENSMUSG00000051855.15	*Mest*	0.82	5.16	51.06	8.96E-13	3.97E-10		
ENSMUSG00000042453.14	*Reln*	0.60	8.79	49.85	1.66E-12	7.10E-10		
ENSMUSG00000046805.9	*Mpeg1*	2.48	1.10	45.73	1.36E-11	5.15E-09		
ENSMUSG00000024376.6	*Epb41l4a*	0.63	5.30	45.09	1.88E-11	6.94E-09		
ENSMUSG00000029093.14	*Sorcs2*	0.79	4.71	44.95	2.03E-11	7.35E-09		
ENSMUSG00000027674.16	*Pex5l*	0.86	4.47	44.02	3.24E-11	1.13E-08		
ENSMUSG00000029088.16	*Kcnip4*	0.87	5.94	43.61	4.01E-11	1.38E-08		
ENSMUSG00000027663.12	*Zmat3*	0.63	5.35	43.36	4.56E-11	1.55E-08		
ENSMUSG00000037605.16	*Adgrl3*	1.04	8.86	43.30	4.71E-11	1.58E-08		
ENSMUSG00000031478.16	*Nek3*	0.72	4.64	42.90	5.75E-11	1.91E-08		
ENSMUSG00000045083.14	*Lingo2*	1.02	6.38	42.62	6.66E-11	2.15E-08		
ENSMUSG00000056427.10	*Slit3*	1.16	7.62	42.37	7.56E-11	2.39E-08		
ENSMUSG00000022762.17	*Ncam2*	0.56	8.35	42.17	8.35E-11	2.61E-08		
ENSMUSG00000021730.8	*Hcn1*	1.33	3.87	39.96	2.59E-10	7.59E-08		
ENSMUSG00000038718.15	*Pbx3*	0.82	7.11	39.52	3.25E-10	9.43E-08		
ENSMUSG00000022074.5	*Tnfrsf10b*	1.73	1.71	39.29	3.65E-10	1.04E-07		
ENSMUSG00000021668.15	*Polk*	0.74	4.47	39.05	4.13E-10	1.15E-07		
ENSMUSG00000043531.16	*Sorcs1*	0.74	6.48	38.54	5.35E-10	1.44E-07		
ENSMUSG00000022148.15	*Fyb*	2.27	1.04	38.10	6.73E-10	1.77E-07		
ENSMUSG00000021448.7	*Shc3*	0.76	6.32	38.08	6.79E-10	1.78E-07		
ENSMUSG00000034825.14	*Nrip3*	1.72	2.09	37.78	7.93E-10	2.05E-07		
ENSMUSG00000015766.14	*Eps8*	0.77	4.14	37.76	8.00E-10	2.06E-07		
ENSMUSG00000033777.3	*Tlr13*	5.13	-0.09	37.59	8.74E-10	2.23E-07		
ENSMUSG00000004609.11	*Cd33*	2.79	0.72	37.49	9.19E-10	2.34E-07		
ENSMUSG00000032625.14	*Thsd7a*	1.55	4.78	37.31	1.01E-09	2.55E-07		
ENSMUSG00000037509.19	*Arhgef4*	0.90	4.55	37.03	1.17E-09	2.91E-07		
ENSMUSG00000026970.16	*Rbms1*	0.67	4.89	36.97	1.20E-09	2.98E-07		
ENSMUSG00000036452.17	*Arhgap26*	0.69	5.44	36.76	1.34E-09	3.27E-07		
ENSMUSG00000036192.15	*Rorb*	0.73	6.06	36.67	1.40E-09	3.41E-07		

ENSMUSG00000063600.14	*Egfem1*	1.30	5.36	36.48	1.54E-09	3.73E-07
ENSMUSG00000026786.14	*Apbb1ip*	2.76	0.79	35.68	2.33E-09	5.41E-07
ENSMUSG00000027419.9	*Pcsk2*	0.63	5.77	35.65	2.36E-09	5.46E-07
ENSMUSG00000028581.17	*Laptm5*	2.78	0.93	35.43	2.65E-09	6.08E-07
ENSMUSG00000070780.11	*Rbm47*	2.65	0.72	35.29	2.84E-09	6.47E-07
ENSMUSG00000063434.5	*Sorcs3*	1.17	3.95	34.96	3.36E-09	7.55E-07
ENSMUSG00000034154.15	*Ino80*	0.46	6.66	34.51	4.24E-09	9.36E-07
ENSMUSG00000003279.16	*Dlgap1*	1.02	7.39	34.50	4.26E-09	9.38E-07
ENSMUSG00000031618.13	*Nr3c2*	1.45	3.06	34.38	4.54E-09	9.85E-07
ENSMUSG00000019852.7	*Arfgef3*	0.82	4.52	34.15	5.10E-09	1.10E-06
ENSMUSG00000026527.13	*Rgs7*	0.69	5.27	34.13	5.15E-09	1.10E-06
ENSMUSG00000051506.16	*Wdfy4*	2.97	0.45	34.03	5.44E-09	1.16E-06
ENSMUSG00000057182.14	*Scn3a*	0.80	5.85	33.97	5.58E-09	1.19E-06
ENSMUSG00000056158.14	*Car10*	1.42	2.44	33.60	6.78E-09	1.41E-06
ENSMUSG00000049420.9	*Tmem200a*	0.86	4.50	33.52	7.05E-09	1.46E-06
ENSMUSG00000042228.14	*Lyn*	1.21	2.58	33.37	7.64E-09	1.55E-06
ENSMUSG00000026312.17	*Cdh7*	0.80	4.34	32.73	1.06E-08	2.11E-06
ENSMUSG00000036896.5	*C1qc*	2.57	0.77	32.72	1.07E-08	2.12E-06
ENSMUSG00000019647.16	*Sema6a*	0.67	6.97	32.47	1.21E-08	2.39E-06
ENSMUSG00000046818.7	*Ddit4l*	1.99	1.64	32.28	1.33E-08	2.59E-06
ENSMUSG00000059921.15	*Unc5c*	1.20	4.36	32.26	1.35E-08	2.61E-06
ENSMUSG00000029816.10	*Gpnmb*	4.69	0.21	32.22	1.38E-08	2.64E-06
ENSMUSG00000031129.9	*Slc9a9*	0.73	3.76	32.05	1.50E-08	2.85E-06
ENSMUSG00000021991.8	*Cacna2d3*	0.84	6.36	31.88	1.64E-08	3.09E-06
ENSMUSG00000042596.7	*Tfap2d*	1.87	3.64	31.78	1.73E-08	3.24E-06
ENSMUSG00000015852.13	*Fcrls*	2.53	0.74	31.47	2.03E-08	3.73E-06
ENSMUSG00000037624.15	*Kcnk2*	0.77	4.92	31.37	2.13E-08	3.89E-06
ENSMUSG00000048756.11	*Foxo3*	0.47	6.20	31.27	2.24E-08	4.07E-06
ENSMUSG00000021557.14	*Agtpbp1*	0.49	6.77	31.18	2.35E-08	4.23E-06
ENSMUSG00000024621.15	*Csf1r*	2.11	1.38	30.70	3.02E-08	5.36E-06
ENSMUSG00000027737.10	*Slc7a11*	2.21	1.54	30.60	3.18E-08	5.59E-06
ENSMUSG00000006219.12	*Fblim1*	1.67	1.55	30.42	3.48E-08	6.10E-06
ENSMUSG00000070695.4	*Cntnap5a*	1.28	3.16	30.22	3.86E-08	6.71E-06
ENSMUSG00000019828.13	*Grm1*	0.84	4.67	30.04	4.24E-08	7.34E-06
ENSMUSG00000007891.16	*Ctsd*	1.30	2.18	29.98	4.37E-08	7.51E-06
ENSMUSG00000106379.1	*Lhfpl3*	1.41	6.02	29.73	4.97E-08	8.48E-06
ENSMUSG00000036402.13	*Gng12*	0.61	4.59	29.58	5.36E-08	9.10E-06
ENSMUSG00000040552.8	*C3ar1*	2.73	0.45	29.37	5.98E-08	1.00E-05
ENSMUSG00000029026.16	*Trp73*	0.93	3.82	29.27	6.30E-08	1.04E-05
ENSMUSG00000039057.11	*Myo16*	0.73	5.89	29.26	6.32E-08	1.04E-05
ENSMUSG00000020143.15	*Dock2*	1.51	1.68	29.15	6.71E-08	1.09E-05
ENSMUSG00000019874.11	*Fabp7*	0.53	5.78	29.09	6.90E-08	1.11E-05
ENSMUSG00000020389.19	*Cdkl3*	0.57	4.92	29.07	6.98E-08	1.12E-05
ENSMUSG00000055540.15	*Epha6*	0.83	4.99	29.04	7.10E-08	1.13E-05
ENSMUSG00000000560.9	*Gabra2*	0.85	6.56	29.02	7.18E-08	1.14E-05
ENSMUSG00000040918.12	*Slc19a2*	1.12	2.65	28.83	7.89E-08	1.24E-05
ENSMUSG00000020431.5	*Adcy1*	0.76	4.77	28.67	8.57E-08	1.33E-05
ENSMUSG00000044912.10	*Syt16*	0.87	5.62	28.65	8.68E-08	1.34E-05
ENSMUSG00000053626.5	*Tll1*	1.84	1.79	28.63	8.74E-08	1.34E-05
ENSMUSG00000038147.13	*Cd84*	2.54	0.79	28.53	9.22E-08	1.41E-05
ENSMUSG00000044071.8	*Fam19a2*	0.95	5.97	28.23	1.08E-07	1.61E-05
ENSMUSG00000035131.14	*Brinp3*	1.37	3.16	28.12	1.14E-07	1.70E-05
ENSMUSG00000025969.15	*Nrp2*	0.53	5.99	28.07	1.17E-07	1.73E-05
ENSMUSG00000027971.16	*Ndst4*	0.70	4.28	27.83	1.32E-07	1.92E-05
ENSMUSG00000028172.5	*Tacr3*	1.56	1.79	27.82	1.33E-07	1.93E-05
ENSMUSG00000036264.9	*Fstl4*	1.97	3.51	27.64	1.46E-07	2.09E-05
ENSMUSG00000037855.15	*Zfp365*	1.58	1.74	27.20	1.84E-07	2.56E-05
ENSMUSG00000029211.11	*Gabra4*	1.04	3.12	27.07	1.96E-07	2.71E-05
ENSMUSG00000026430.16	*Rassf5*	2.22	0.89	27.06	1.98E-07	2.73E-05
ENSMUSG00000031075.18	*Ano1*	1.60	3.38	26.82	2.24E-07	3.07E-05
ENSMUSG00000051920.6	*Rspo2*	3.57	2.65	26.78	2.27E-07	3.08E-05
ENSMUSG00000042256.4	*Ptchd4*	0.69	5.47	26.55	2.57E-07	3.46E-05
ENSMUSG00000002944.15	*Cd36*	3.08	0.20	26.45	2.71E-07	3.61E-05
ENSMUSG00000035270.15	*Impg2*	0.95	3.60	26.26	2.98E-07	3.97E-05
ENSMUSG00000025790.14	*Slco3a1*	0.54	7.21	26.18	3.10E-07	4.11E-05
ENSMUSG00000050663.7	*Trhde*	1.42	2.11	26.17	3.12E-07	4.13E-05
ENSMUSG00000004113.18	*Cacna1b*	0.72	6.31	26.01	3.40E-07	4.48E-05
ENSMUSG00000026048.16	*Ercc5*	0.44	5.94	25.49	4.45E-07	5.77E-05
ENSMUSG00000021466.12	*Ptch1*	0.84	4.12	25.36	4.75E-07	6.09E-05
ENSMUSG00000059895.12	*Ptp4a3*	0.86	3.08	25.21	5.15E-07	6.54E-05
ENSMUSG00000062762.16	*Ei24*	0.53	5.09	24.92	5.97E-07	7.42E-05
ENSMUSG00000068748.7	*Ptprz1*	0.48	8.62	24.92	5.97E-07	7.42E-05

ENSMUSG00000026604.17	*Ptpn14*	1.56	2.45	24.74	6.55E-07	8.05E-05
ENSMUSG00000021939.7	*Ctsb*	0.65	5.12	24.72	6.64E-07	8.14E-05
ENSMUSG00000044667.12	*Plppr4*	1.02	2.50	24.31	8.20E-07	9.88E-05
ENSMUSG00000025938.16	*Slco5a1*	0.46	6.10	24.24	8.52E-07	0.000102
ENSMUSG00000029287.14	*Tgfbr3*	1.61	2.08	24.10	9.13E-07	0.000108
ENSMUSG00000030518.17	*Fam189a1*	0.71	3.96	24.03	9.49E-07	0.000112
ENSMUSG00000044042.18	*Fmn1*	1.69	1.07	23.97	9.77E-07	0.000115
ENSMUSG00000055022.14	*Cntn1*	0.70	5.15	23.90	1.02E-06	0.000119
ENSMUSG00000021190.14	*Lgmn*	0.63	4.04	23.81	1.07E-06	0.000124
ENSMUSG00000033676.13	*Gabrb3*	0.72	7.30	23.75	1.10E-06	0.000127
ENSMUSG00000032577.16	*Mapkapk3*	1.54	1.46	23.31	1.38E-06	0.000156
ENSMUSG00000028937.14	*Acot7*	0.50	5.23	23.23	1.44E-06	0.000162
ENSMUSG00000024565.8	*Sall3*	0.76	3.53	23.10	1.54E-06	0.000171
ENSMUSG00000004730.14	*Adgre1*	1.92	0.90	22.97	1.65E-06	0.000181
ENSMUSG00000036466.17	*Megf11*	0.82	4.05	22.89	1.71E-06	0.000187
ENSMUSG00000015950.13	*Ncf1*	1.66	1.02	22.85	1.75E-06	0.000191
ENSMUSG00000032338.9	*Hcn4*	0.78	3.46	22.79	1.80E-06	0.000195
ENSMUSG00000030020.13	*Prickle2*	0.61	6.18	22.74	1.85E-06	0.000200
ENSMUSG00000044176.11	*Spink10*	0.49	5.45	22.73	1.86E-06	0.000201
ENSMUSG00000024300.16	*Myo1f*	2.47	0.73	22.54	2.06E-06	0.000219
ENSMUSG00000058975.7	*Kcnc1*	0.74	4.60	22.53	2.07E-06	0.000220
ENSMUSG00000044252.17	*Osbpl1a*	0.46	5.61	22.53	2.07E-06	0.000220
ENSMUSG00000036905.8	*C1qb*	2.18	0.56	22.48	2.12E-06	0.000224
ENSMUSG00000027016.17	*Zfp385b*	1.35	2.56	22.40	2.21E-06	0.000233
ENSMUSG00000052387.15	*Trpm3*	0.79	6.27	22.32	2.30E-06	0.000242
ENSMUSG00000079022.9	*Col22a1*	1.37	4.00	22.14	2.53E-06	0.000262
ENSMUSG00000051951.5	*Xkr4*	0.71	7.20	21.97	2.77E-06	0.000283
ENSMUSG00000036887.5	*C1qa*	3.29	0.12	21.94	2.82E-06	0.000286
ENSMUSG00000042851.17	*Zc3h6*	0.72	5.05	21.74	3.12E-06	0.000313
ENSMUSG00000005873.5	*Reep5*	0.54	4.50	21.68	3.23E-06	0.000323
ENSMUSG00000010066.15	*Cacna2d2*	0.69	4.50	21.61	3.34E-06	0.000332
ENSMUSG00000033308.16	*Dpyd*	0.68	4.21	21.54	3.47E-06	0.000342
ENSMUSG00000009876.13	*Cox4i2*	2.02	0.56	21.39	3.75E-06	0.000368
ENSMUSG00000022523.9	*Fgf12*	0.80	4.91	21.05	4.48E-06	0.000431
ENSMUSG00000056755.13	*Grm7*	0.83	5.12	20.89	4.85E-06	0.000463
ENSMUSG00000053025.13	*Sv2b*	0.56	5.41	20.90	4.85E-06	0.000463
ENSMUSG00000022935.15	*Grik1*	0.56	6.32	20.81	5.07E-06	0.000480
ENSMUSG00000032452.12	*Clstn2*	0.62	6.93	20.75	5.24E-06	0.000491
ENSMUSG00000020902.12	*Ntn1*	0.89	2.86	20.56	5.79E-06	0.000533
ENSMUSG00000026288.14	*Inpp5d*	1.22	2.20	20.46	6.09E-06	0.000553
ENSMUSG00000041710.4	*Trpc5*	1.17	3.10	20.46	6.10E-06	0.000553
ENSMUSG00000036006.19	*Ripor2*	0.79	3.99	20.18	7.03E-06	0.000625
ENSMUSG00000040855.15	*Reps2*	0.87	3.50	20.15	7.15E-06	0.000634
ENSMUSG00000037725.8	*Ckap2*	0.41	5.28	20.10	7.36E-06	0.000651
ENSMUSG00000011256.16	*Adam19*	0.58	4.88	20.09	7.38E-06	0.000651
ENSMUSG00000019880.10	*Rspo3*	0.76	5.22	20.08	7.44E-06	0.000655
ENSMUSG00000040420.14	*Cdh18*	1.05	2.44	20.03	7.64E-06	0.000669
ENSMUSG00000062151.13	*Unc13c*	1.74	2.64	19.99	7.77E-06	0.000678
ENSMUSG00000041272.11	*Tox*	0.60	7.84	19.90	8.18E-06	0.000707
ENSMUSG00000040265.16	*Dnm3*	0.82	5.88	19.79	8.63E-06	0.000744
ENSMUSG00000051354.14	*Samd3*	1.90	1.32	19.77	8.72E-06	0.000751
ENSMUSG00000057897.14	*Camk2b*	0.76	5.03	19.74	8.86E-06	0.000760
ENSMUSG00000022817.14	*Itgb5*	1.38	2.02	19.72	8.95E-06	0.000767
ENSMUSG00000061576.15	*Dpp6*	0.58	7.32	19.66	9.23E-06	0.000790
ENSMUSG00000005413.8	*Hmox1*	1.44	1.46	19.52	9.94E-06	0.000845
ENSMUSG00000049583.15	*Grm5*	0.95	5.74	19.51	1.00E-05	0.000850
ENSMUSG00000058672.7	*Tubb2a*	0.62	4.21	19.39	1.06E-05	0.000897
ENSMUSG00000045438.12	*Cox19*	0.64	3.59	19.37	1.08E-05	0.000906
ENSMUSG00000033910.13	*Gucy1a3*	0.80	3.21	19.35	1.09E-05	0.000913
ENSMUSG00000022901.13	*Cd86*	2.83	0.09	19.29	1.12E-05	0.000940
ENSMUSG00000075316.11	*Scn9a*	1.32	2.14	19.29	1.12E-05	0.000940
ENSMUSG00000045658.16	*Pid1*	0.66	6.41	19.28	1.13E-05	0.000943
ENSMUSG00000020096.20	*Tbata*	1.97	0.54	19.24	1.15E-05	0.000958
ENSMUSG00000047495.15	*Dlgap2*	0.81	6.89	19.23	1.16E-05	0.000959
ENSMUSG00000004631.15	*Sgce*	0.42	5.89	19.19	1.18E-05	0.000972

P-value calculated with likelihood ratio tests. Adjusted P-value for multiple testing calculated using the Benjamini-Hochberg method (FDR).

Supplementary Table 2. Significantly downregulated genes by UMI RNA-seq (cKO-E vs. ctrl, FDR < 0.001)

Ensembl Mouse Gene ID	Gene symbol	logFC	logCPM	LR	PValue	FDR
ENSMUSG00000017692.8	*Rhbdl3*	-1.17	6.76	192.27	1.02E-43	1.76E-39
ENSMUSG00000034462.9	*Pkd2*	-1.05	5.96	142.05	9.50E-33	4.93E-29
ENSMUSG00000032238.17	*Rora*	-1.01	7.78	134.06	5.29E-31	2.50E-27
ENSMUSG00000022672.8	*Prkdc*	-1.03	6.14	130.24	3.63E-30	1.57E-26
ENSMUSG00000040943.12	*Tet2*	-0.98	6.83	127.03	1.83E-29	6.79E-26
ENSMUSG00000030061.16	*Uba3*	-1.05	5.66	104.61	1.49E-24	4.84E-21
ENSMUSG00000058729.13	*Lin9*	-1.06	5.33	103.34	2.83E-24	8.64E-21
ENSMUSG00000036371.6	*Serbp1*	-0.81	7.33	96.14	1.07E-22	2.77E-19
ENSMUSG00000027204.13	*Fbn1*	-1.01	5.23	91.17	1.32E-21	3.12E-18
ENSMUSG00000021846.8	*Peli2*	-0.93	6.83	85.41	2.42E-20	4.50E-17
ENSMUSG00000034687.8	*Fras1*	-0.72	7.42	84.88	3.18E-20	5.32E-17
ENSMUSG00000037386.15	*Rims2*	-0.82	8.08	83.36	6.82E-20	1.11E-16
ENSMUSG00000084799.7	*Ino80dos*	-1.20	4.31	81.28	1.96E-19	3.00E-16
ENSMUSG00000057914.15	*Cacnb2*	-0.98	7.48	80.87	2.41E-19	3.57E-16
ENSMUSG00000034295.9	*Fhod3*	-0.68	7.87	80.71	2.62E-19	3.77E-16
ENSMUSG00000020564.17	*Atxn7l1*	-0.68	7.45	80.60	2.77E-19	3.89E-16
ENSMUSG00000025154.14	*Arhgap19*	-1.33	4.50	80.29	3.24E-19	4.42E-16
ENSMUSG00000039967.14	*Zfp292*	-0.69	8.27	76.55	2.15E-18	2.72E-15
ENSMUSG00000031284.16	*Pak3*	-0.67	8.11	72.72	1.50E-17	1.81E-14
ENSMUSG00000056476.13	*Med12l*	-0.74	6.94	72.46	1.71E-17	1.93E-14
ENSMUSG00000024940.10	*Ltbp3*	-1.06	4.25	72.14	2.01E-17	2.22E-14
ENSMUSG00000036617.16	*Etl4*	-0.74	9.27	69.60	7.27E-17	7.86E-14
ENSMUSG00000002459.17	*Rgs20*	-0.79	7.26	69.45	7.85E-17	8.15E-14
ENSMUSG00000051339.10	*2900026A02Rik*	-0.91	5.10	69.49	7.70E-17	8.15E-14
ENSMUSG00000021379.1	*Id4*	-1.07	6.88	68.01	1.63E-16	1.66E-13
ENSMUSG00000036023.5	*Parp2*	-1.21	3.86	67.92	1.70E-16	1.70E-13
ENSMUSG00000018501.17	*Ncor1*	-0.68	8.37	66.59	3.35E-16	3.16E-13
ENSMUSG00000014426.8	*Map3k4*	-0.63	6.09	66.44	3.60E-16	3.34E-13
ENSMUSG00000040488.17	*Ltbp4*	-1.26	3.82	66.22	4.04E-16	3.56E-13
ENSMUSG00000042686.5	*Jph1*	-0.87	6.79	64.77	8.41E-16	7.04E-13
ENSMUSG00000038473.14	*Nos1ap*	-0.56	7.66	63.69	1.45E-15	1.18E-12
ENSMUSG00000004328.15	*Hif3a*	-0.93	4.42	62.86	2.22E-15	1.77E-12
ENSMUSG00000091722.1	*Siah3*	-0.77	7.29	62.41	2.79E-15	2.20E-12
ENSMUSG00000055799.13	*Tcf7l1*	-0.82	8.08	62.29	2.96E-15	2.30E-12
ENSMUSG00000027560.4	*Dok5*	-1.01	6.06	62.13	3.21E-15	2.45E-12
ENSMUSG00000038855.10	*Itpkb*	-1.27	4.16	61.75	3.90E-15	2.86E-12
ENSMUSG00000028437.14	*Ubap1*	-0.68	5.82	61.65	4.11E-15	2.96E-12
ENSMUSG00000029769.16	*Ccdc136*	-0.81	6.41	61.14	5.32E-15	3.78E-12
ENSMUSG00000004562.16	*Arhgef40*	-0.79	5.23	60.40	7.76E-15	5.30E-12
ENSMUSG00000031109.16	*Enox2*	-0.63	6.56	60.08	9.13E-15	6.15E-12
ENSMUSG00000035967.15	*Ints6l*	-0.80	5.23	59.96	9.69E-15	6.45E-12
ENSMUSG00000002688.8	*Prkd1*	-0.71	7.67	59.41	1.28E-14	8.41E-12
ENSMUSG00000028456.18	*Unc13b*	-0.65	7.09	58.87	1.68E-14	1.08E-11
ENSMUSG00000021027.16	*Ralgapa1*	-0.68	7.37	57.48	3.41E-14	2.16E-11
ENSMUSG00000063888.6	*Rpl7l1*	-0.79	4.79	57.25	3.84E-14	2.40E-11
ENSMUSG00000031333.7	*Abcb7*	-0.58	6.13	57.02	4.31E-14	2.64E-11
ENSMUSG00000029705.17	*Cux1*	-0.60	8.76	57.00	4.36E-14	2.64E-11
ENSMUSG00000063446.4	*Plppr1*	-0.73	8.48	56.99	4.38E-14	2.64E-11
ENSMUSG00000022483.16	*Col2a1*	-1.26	4.76	56.66	5.17E-14	3.09E-11
ENSMUSG00000021338.17	*Carmil1*	-0.73	6.14	56.36	6.02E-14	3.55E-11
ENSMUSG00000023991.16	*Foxp4*	-0.81	7.15	56.14	6.74E-14	3.93E-11
ENSMUSG00000039396.11	*Neil3*	-0.64	6.19	55.99	7.30E-14	4.21E-11
ENSMUSG00000021577.14	*Sdha*	-0.67	5.51	55.85	7.83E-14	4.47E-11
ENSMUSG00000038070.15	*Cntln*	-0.66	6.40	55.67	8.58E-14	4.84E-11
ENSMUSG00000023092.16	*Fhl1*	-0.78	6.13	55.54	9.16E-14	5.11E-11
ENSMUSG00000059493.13	*Nhs*	-0.75	7.85	55.12	1.14E-13	6.28E-11
ENSMUSG00000001127.12	*Araf*	-0.65	6.21	54.87	1.29E-13	7.03E-11
ENSMUSG00000033502.14	*Cdc14a*	-0.60	6.97	54.70	1.40E-13	7.59E-11
ENSMUSG00000039765.15	*Cc2d2a*	-1.02	4.54	54.35	1.68E-13	8.98E-11
ENSMUSG00000041220.10	*Elovl6*	-0.64	7.27	54.21	1.80E-13	9.43E-11
ENSMUSG00000032220.10	*Myo1e*	-0.98	4.52	53.82	2.20E-13	1.14E-10
ENSMUSG00000041921.16	*Metap1d*	-0.74	4.85	53.78	2.24E-13	1.15E-10
ENSMUSG00000003360.14	*Ddx23*	-0.85	4.82	53.61	2.45E-13	1.24E-10
ENSMUSG00000035934.16	*Pknox2*	-0.66	6.47	53.21	2.99E-13	1.51E-10
ENSMUSG00000029104.15	*Htt*	-0.58	6.92	53.11	3.15E-13	1.57E-10
ENSMUSG00000036867.7	*Smad6*	-1.01	4.48	53.10	3.17E-13	1.57E-10
ENSMUSG00000035798.14	*Zdhhc17*	-0.62	6.24	52.90	3.52E-13	1.72E-10
ENSMUSG00000032263.14	*Bckdhb*	-0.77	5.52	52.85	3.59E-13	1.74E-10

ENSMUSG00000020463.15	*Ppp4r3b*	-0.57	6.61	52.73	3.82E-13	1.82E-10
ENSMUSG00000035704.17	*Alg8*	-0.98	4.13	52.17	5.09E-13	2.40E-10
ENSMUSG00000071064.13	*Zfp827*	-0.71	7.47	51.53	7.07E-13	3.30E-10
ENSMUSG00000032397.7	*Tipin*	-0.76	5.22	51.51	7.13E-13	3.30E-10
ENSMUSG00000000247.11	*Lhx2*	-0.68	7.49	51.26	8.08E-13	3.62E-10
ENSMUSG00000025812.17	*Pard3*	-0.70	8.98	51.03	9.09E-13	4.00E-10
ENSMUSG00000063760.9	*Rnf217*	-0.60	6.35	50.24	1.36E-12	5.92E-10
ENSMUSG00000026275.13	*Ppp1r7*	-0.67	5.17	50.01	1.53E-12	6.60E-10
ENSMUSG00000030350.8	*Prmt8*	-1.24	5.42	49.84	1.67E-12	7.10E-10
ENSMUSG00000090626.9	*Tex9*	-0.73	5.62	49.66	1.83E-12	7.73E-10
ENSMUSG00000030428.16	*Ttyh1*	-0.73	6.79	48.69	3.00E-12	1.26E-09
ENSMUSG00000029334.14	*Prkg2*	-1.33	4.10	48.44	3.41E-12	1.41E-09
ENSMUSG00000033149.16	*Phldb2*	-0.73	5.56	47.72	4.91E-12	2.02E-09
ENSMUSG00000019951.10	*Uhrf1bp1l*	-0.57	6.22	47.67	5.03E-12	2.06E-09
ENSMUSG00000032050.17	*Rdx*	-0.53	7.38	47.09	6.78E-12	2.75E-09
ENSMUSG00000001911.16	*Nfix*	-0.65	8.67	46.67	8.39E-12	3.38E-09
ENSMUSG00000020961.15	*Ston2*	-0.66	6.57	46.49	9.23E-12	3.63E-09
ENSMUSG00000006678.6	*Pola1*	-0.66	7.50	46.49	9.20E-12	3.63E-09
ENSMUSG00000021669.15	*Col4a3bp*	-0.69	5.83	46.49	9.22E-12	3.63E-09
ENSMUSG00000042225.3	*Ammecr1*	-0.67	5.38	46.33	1.00E-11	3.87E-09
ENSMUSG00000023411.11	*Nfatc4*	-1.20	3.55	46.34	9.96E-12	3.87E-09
ENSMUSG00000026826.13	*Nr4a2*	-0.99	4.61	45.91	1.24E-11	4.77E-09
ENSMUSG00000056014.15	*A430033K04Rik*	-0.86	4.46	45.83	1.29E-11	4.92E-09
ENSMUSG00000028080.16	*Lrba*	-0.58	6.08	45.49	1.53E-11	5.76E-09
ENSMUSG00000035021.13	*Baz1a*	-0.62	6.26	45.27	1.71E-11	6.40E-09
ENSMUSG00000002870.8	*Mcm2*	-0.82	6.13	45.09	1.88E-11	6.94E-09
ENSMUSG00000065954.11	*Tacc1*	-0.58	6.62	45.01	1.96E-11	7.17E-09
ENSMUSG00000031684.11	*Slc10a7*	-0.54	6.73	44.85	2.13E-11	7.64E-09
ENSMUSG00000097023.8	*Mir9-3hg*	-0.55	8.76	44.85	2.13E-11	7.64E-09
ENSMUSG00000053519.15	*Kcnip1*	-0.88	6.49	44.72	2.27E-11	8.08E-09
ENSMUSG00000021938.11	*Pspc1*	-0.55	7.30	44.31	2.81E-11	9.92E-09
ENSMUSG00000064105.12	*Cnnm2*	-0.89	4.04	44.02	3.25E-11	1.13E-08
ENSMUSG00000070509.15	*Rgma*	-0.71	6.67	44.00	3.29E-11	1.14E-08
ENSMUSG00000026235.14	*Epha4*	-0.53	9.12	43.40	4.47E-11	1.53E-08
ENSMUSG00000029086.15	*Prom1*	-0.67	6.65	43.29	4.71E-11	1.58E-08
ENSMUSG00000033610.15	*Pank1*	-0.72	5.12	42.85	5.90E-11	1.95E-08
ENSMUSG00000055471.6	*Alk*	-0.97	5.60	42.81	6.02E-11	1.98E-08
ENSMUSG00000059208.14	*Hnrnpm*	-0.49	7.96	42.80	6.07E-11	1.98E-08
ENSMUSG00000048661.14	*Lemd3*	-0.50	6.28	42.76	6.18E-11	2.00E-08
ENSMUSG00000022641.15	*Bbx*	-0.55	8.18	42.52	6.99E-11	2.24E-08
ENSMUSG00000036377.18	*C530008M17Rik*	-0.53	8.76	42.49	7.10E-11	2.26E-08
ENSMUSG00000038119.15	*Cdon*	-0.75	9.54	42.23	8.09E-11	2.55E-08
ENSMUSG00000039470.15	*Zdhhc2*	-0.94	4.47	41.89	9.66E-11	3.00E-08
ENSMUSG00000039968.9	*Rsbn1l*	-0.56	5.89	41.58	1.13E-10	3.50E-08
ENSMUSG00000028514.15	*Usp24*	-0.50	7.07	41.37	1.26E-10	3.87E-08
ENSMUSG00000042507.15	*Elmsan1*	-0.71	5.47	41.08	1.46E-10	4.46E-08
ENSMUSG00000025810.9	*Nrp1*	-0.72	7.38	40.82	1.67E-10	5.07E-08
ENSMUSG00000032498.9	*Mlh1*	-0.76	4.74	40.72	1.76E-10	5.30E-08
ENSMUSG00000035227.7	*Spcs2*	-0.72	4.84	40.58	1.89E-10	5.67E-08
ENSMUSG00000017390.15	*Aldoc*	-0.79	4.55	40.52	1.95E-10	5.81E-08
ENSMUSG00000031665.7	*Sall1*	-0.76	6.67	40.37	2.10E-10	6.23E-08
ENSMUSG00000024975.12	*Pdcd4*	-0.52	6.94	40.11	2.40E-10	7.07E-08
ENSMUSG00000039089.15	*L3mbtl3*	-0.59	6.00	39.95	2.60E-10	7.59E-08
ENSMUSG00000022636.13	*Alcam*	-0.69	8.68	39.46	3.35E-10	9.66E-08
ENSMUSG00000032009.8	*Sesn3*	-0.65	5.90	39.34	3.56E-10	1.02E-07
ENSMUSG00000052812.5	*Atad2b*	-0.53	7.37	39.26	3.71E-10	1.05E-07
ENSMUSG00000036298.10	*Slc2a13*	-0.73	6.81	39.16	3.90E-10	1.10E-07
ENSMUSG00000025103.8	*Btbd1*	-0.52	5.92	39.10	4.02E-10	1.12E-07
ENSMUSG00000054920.12	*Klhl5*	-0.58	6.03	39.11	4.01E-10	1.12E-07
ENSMUSG00000046961.7	*Gpr156*	-0.97	4.47	38.99	4.27E-10	1.18E-07
ENSMUSG00000035234.18	*Abraxas1*	-1.05	3.50	38.92	4.41E-10	1.21E-07
ENSMUSG00000037957.14	*Wdr20*	-0.48	6.24	38.72	4.90E-10	1.34E-07
ENSMUSG00000053007.9	*Creb5*	-0.61	9.82	38.56	5.32E-10	1.44E-07
ENSMUSG00000037995.15	*Igsf9*	-0.62	5.40	38.56	5.32E-10	1.44E-07
ENSMUSG00000026220.6	*Slc16a14*	-1.07	3.87	38.43	5.69E-10	1.52E-07
ENSMUSG00000017548.15	*Suz12*	-0.56	7.08	38.37	5.84E-10	1.56E-07
ENSMUSG00000058325.6	*Dock1*	-0.61	7.35	38.23	6.29E-10	1.67E-07
ENSMUSG00000028517.8	*Plpp3*	-0.58	7.60	38.02	7.02E-10	1.83E-07
ENSMUSG00000033720.12	*Sfxn5*	-0.67	6.57	37.86	7.62E-10	1.98E-07
ENSMUSG00000087259.7	*2610035D17Rik*	-0.62	8.54	37.05	1.15E-09	2.89E-07
ENSMUSG00000070643.11	*Sox13*	-1.06	4.46	37.05	1.15E-09	2.89E-07
ENSMUSG00000033732.10	*Sf3b3*	-0.49	6.82	36.83	1.29E-09	3.19E-07

ENSMUSG00000025925.14	*Terf1*	-0.61	5.50	36.81	1.31E-09	3.21E-07
ENSMUSG00000021375.9	*Kif13a*	-0.60	6.03	36.48	1.54E-09	3.73E-07
ENSMUSG00000017978.18	*Cadps2*	-0.60	5.31	36.44	1.57E-09	3.77E-07
ENSMUSG00000033420.12	*Antxr1*	-0.62	5.44	36.44	1.57E-09	3.77E-07
ENSMUSG00000021957.6	*Tkt*	-0.48	6.14	36.40	1.60E-09	3.82E-07
ENSMUSG00000047454.12	*Gphn*	-0.53	7.52	36.12	1.85E-09	4.39E-07
ENSMUSG00000016386.15	*Mpped2*	-0.85	9.96	36.08	1.89E-09	4.46E-07
ENSMUSG00000038280.12	*Ostm1*	-0.48	7.05	35.75	2.24E-09	5.27E-07
ENSMUSG00000027203.15	*Dut*	-0.63	4.94	35.74	2.26E-09	5.28E-07
ENSMUSG00000030022.14	*Adamts9*	-1.46	4.80	35.51	2.54E-09	5.85E-07
ENSMUSG00000019889.10	*Ptprk*	-0.55	7.95	35.41	2.66E-09	6.09E-07
ENSMUSG00000034109.15	*Golim4*	-0.48	6.66	35.21	2.95E-09	6.70E-07
ENSMUSG00000038526.14	*Car14*	-1.98	2.51	35.08	3.16E-09	7.14E-07
ENSMUSG00000052613.16	*Pcdh15*	-0.86	6.16	34.84	3.58E-09	8.01E-07
ENSMUSG00000028444.17	*Cntfr*	-0.79	6.15	34.60	4.04E-09	9.00E-07
ENSMUSG00000041997.16	*Tlk1*	-0.42	7.27	34.55	4.15E-09	9.20E-07
ENSMUSG00000029563.16	*Foxp2*	-0.61	8.44	34.47	4.33E-09	9.49E-07
ENSMUSG00000061315.14	*Naca*	-0.52	5.77	34.44	4.39E-09	9.58E-07
ENSMUSG00000026614.6	*Slc30a10*	-0.69	5.91	34.21	4.96E-09	1.07E-06
ENSMUSG00000031290.14	*Lrch2*	-0.60	6.51	33.79	6.14E-09	1.30E-06
ENSMUSG00000036768.6	*Kif15*	-0.57	6.88	33.64	6.63E-09	1.40E-06
ENSMUSG00000006930.15	*Hap1*	-0.89	4.25	33.63	6.67E-09	1.40E-06
ENSMUSG00000031273.16	*Col4a6*	-1.02	3.61	33.61	6.73E-09	1.41E-06
ENSMUSG00000039384.8	*Dusp10*	-0.82	4.29	33.57	6.87E-09	1.43E-06
ENSMUSG00000031990.15	*Jam3*	-0.53	6.32	33.51	7.09E-09	1.46E-06
ENSMUSG00000051022.7	*Hs3st1*	-0.66	5.24	33.48	7.21E-09	1.48E-06
ENSMUSG00000029782.19	*Tmem209*	-0.75	4.51	33.40	7.52E-09	1.54E-06
ENSMUSG00000045179.9	*Sox3*	-0.80	4.96	33.29	7.93E-09	1.61E-06
ENSMUSG00000026987.16	*Baz2b*	-0.46	9.14	33.20	8.33E-09	1.68E-06
ENSMUSG00000052848.7	*C130026L21Rik*	-1.20	2.88	33.11	8.72E-09	1.75E-06
ENSMUSG00000025026.14	*Add3*	-0.45	6.15	32.85	9.96E-09	2.00E-06
ENSMUSG00000055296.14	*Tmem245*	-0.49	6.59	32.57	1.15E-08	2.28E-06
ENSMUSG00000037846.18	*Rtkn2*	-0.68	5.18	32.46	1.21E-08	2.39E-06
ENSMUSG00000032040.15	*Dcps*	-0.96	3.88	32.46	1.22E-08	2.39E-06
ENSMUSG00000020963.15	*Tshr*	-0.85	4.68	32.36	1.28E-08	2.50E-06
ENSMUSG00000034573.14	*Ptpn13*	-0.57	5.56	32.27	1.34E-08	2.60E-06
ENSMUSG00000020176.17	*Grb10*	-0.51	8.59	32.22	1.37E-08	2.64E-06
ENSMUSG00000022129.3	*Dct*	-1.68	3.66	32.17	1.41E-08	2.70E-06
ENSMUSG00000016028.9	*Celsr1*	-0.43	8.00	32.05	1.50E-08	2.85E-06
ENSMUSG00000025609.15	*Mkln1*	-0.44	7.11	31.98	1.56E-08	2.95E-06
ENSMUSG00000055024.12	*Ep300*	-0.43	7.16	31.74	1.76E-08	3.28E-06
ENSMUSG00000054453.11	*Sytl5*	-0.89	4.17	31.56	1.93E-08	3.59E-06
ENSMUSG00000032249.14	*Anp32a*	-0.43	7.37	31.53	1.96E-08	3.63E-06
ENSMUSG00000026556.15	*Vangl2*	-0.45	7.44	31.48	2.01E-08	3.72E-06
ENSMUSG00000029179.14	*Zcchc4*	-0.64	4.76	31.45	2.04E-08	3.75E-06
ENSMUSG00000073557.11	*Ppp1r12b*	-0.49	6.89	31.37	2.13E-08	3.89E-06
ENSMUSG00000034402.3	*Kcnh5*	-0.72	6.07	31.25	2.27E-08	4.11E-06
ENSMUSG00000027650.12	*Tti1*	-0.80	4.51	31.17	2.37E-08	4.25E-06
ENSMUSG00000051910.13	*Sox6*	-0.54	10.09	31.11	2.43E-08	4.35E-06
ENSMUSG00000028664.14	*Ephb2*	-0.42	8.16	31.06	2.50E-08	4.46E-06
ENSMUSG00000050271.12	*Prag1*	-0.85	5.26	30.67	3.05E-08	5.41E-06
ENSMUSG00000022463.7	*Srebf2*	-0.42	7.06	30.62	3.13E-08	5.53E-06
ENSMUSG00000016984.7	*Etaa1*	-0.69	4.61	30.32	3.65E-08	6.39E-06
ENSMUSG00000020955.9	*Ap4s1*	-0.60	4.60	30.30	3.71E-08	6.46E-06
ENSMUSG00000022203.6	*Efs*	-0.71	4.18	29.99	4.34E-08	7.48E-06
ENSMUSG00000027253.15	*Lrp4*	-0.51	6.49	29.68	5.10E-08	8.67E-06
ENSMUSG00000057604.9	*Lmcd1*	-1.11	3.14	29.47	5.67E-08	9.58E-06
ENSMUSG00000000085.16	*Scmh1*	-0.42	6.98	29.45	5.74E-08	9.68E-06
ENSMUSG00000034285.15	*Nipsnap1*	-0.68	4.80	29.38	5.95E-08	1.00E-05
ENSMUSG00000098318.7	*Lockd*	-0.51	5.91	29.30	6.19E-08	1.03E-05
ENSMUSG00000059401.13	*Mamld1*	-0.66	6.59	29.29	6.22E-08	1.03E-05
ENSMUSG00000079056.12	*Kcnip3*	-0.80	5.09	29.28	6.26E-08	1.04E-05
ENSMUSG00000037286.15	*Stag1*	-0.39	8.43	29.20	6.52E-08	1.07E-05
ENSMUSG00000060126.14	*Tpt1*	-0.39	8.17	29.14	6.72E-08	1.09E-05
ENSMUSG00000021130.8	*Galnt16*	-0.55	5.97	29.15	6.70E-08	1.09E-05
ENSMUSG00000022489.5	*Pde1b*	-0.53	5.81	29.14	6.75E-08	1.09E-05
ENSMUSG00000026187.8	*Xrcc5*	-0.46	5.56	29.10	6.86E-08	1.11E-05
ENSMUSG00000021767.16	*Kat6b*	-0.47	9.33	29.10	6.87E-08	1.11E-05
ENSMUSG00000021981.9	*Cab39l*	-0.67	4.46	28.99	7.29E-08	1.16E-05
ENSMUSG00000005882.18	*Uqcc1*	-0.51	5.45	28.94	7.45E-08	1.18E-05
ENSMUSG00000015224.10	*Cyp2j9*	-1.59	1.93	28.88	7.70E-08	1.21E-05
ENSMUSG00000097063.3	*Pantr2*	-0.71	4.62	28.82	7.96E-08	1.25E-05

ENSMUSG00000027428.9	*Rbbp9*	-0.84	3.56	28.71	8.42E-08	1.32E-05
ENSMUSG00000046591.10	*Ticrr*	-0.65	5.43	28.70	8.46E-08	1.32E-05
ENSMUSG00000040204.6	*Pclaf*	-0.64	5.06	28.69	8.49E-08	1.32E-05
ENSMUSG00000024400.15	*Wdr33*	-0.37	7.66	28.67	8.58E-08	1.33E-05
ENSMUSG00000030846.15	*Tial1*	-0.45	6.36	28.58	8.97E-08	1.37E-05
ENSMUSG00000032193.9	*Ldlr*	-0.55	5.19	28.52	9.26E-08	1.41E-05
ENSMUSG00000002996.17	*Hbp1*	-0.57	5.54	28.49	9.41E-08	1.43E-05
ENSMUSG00000086370.8	*Ftx*	-0.55	5.53	28.46	9.58E-08	1.45E-05
ENSMUSG00000058881.12	*Zfp516*	-0.47	7.10	28.41	9.82E-08	1.48E-05
ENSMUSG00000022802.2	*Lmln*	-0.64	4.58	28.38	9.99E-08	1.50E-05
ENSMUSG00000075254.11	*Heg1*	-0.50	8.12	28.26	1.06E-07	1.59E-05
ENSMUSG00000087095.2	*Emx2os*	-0.76	5.40	28.08	1.17E-07	1.73E-05
ENSMUSG00000021782.14	*Dlg5*	-0.42	6.57	28.04	1.19E-07	1.75E-05
ENSMUSG00000047045.17	*Tmem164*	-0.47	6.94	28.03	1.19E-07	1.76E-05
ENSMUSG00000015189.12	*Casd1*	-0.54	5.38	27.94	1.25E-07	1.84E-05
ENSMUSG00000061838.7	*Suclg2*	-0.67	5.55	27.93	1.26E-07	1.84E-05
ENSMUSG00000063681.14	*Crb1*	-1.06	3.77	27.91	1.27E-07	1.85E-05
ENSMUSG00000045962.16	*Wnk1*	-0.38	8.03	27.79	1.35E-07	1.95E-05
ENSMUSG00000069806.5	*Cacng7*	-0.46	5.95	27.72	1.40E-07	2.02E-05
ENSMUSG00000019907.10	*Ppp1r12a*	-0.40	7.44	27.67	1.44E-07	2.07E-05
ENSMUSG00000019803.11	*Nr2e1*	-0.58	5.77	27.51	1.57E-07	2.24E-05
ENSMUSG00000029432.12	*Nipsnap2*	-0.62	4.62	27.49	1.58E-07	2.26E-05
ENSMUSG00000039753.16	*Fbxl5*	-0.44	6.22	27.45	1.61E-07	2.29E-05
ENSMUSG00000047712.8	*Ust*	-0.62	6.47	27.37	1.68E-07	2.39E-05
ENSMUSG00000057337.13	*Chst3*	-0.66	4.97	27.26	1.78E-07	2.51E-05
ENSMUSG00000037486.18	*Asxl2*	-0.42	6.84	27.26	1.78E-07	2.51E-05
ENSMUSG00000001435.15	*Col18a1*	-0.81	5.50	27.25	1.79E-07	2.51E-05
ENSMUSG00000057147.13	*Dph6*	-0.43	6.13	27.22	1.81E-07	2.55E-05
ENSMUSG00000027332.11	*Ivd*	-0.67	4.33	27.20	1.83E-07	2.56E-05
ENSMUSG00000019982.15	*Myb*	-0.85	4.05	27.17	1.86E-07	2.59E-05
ENSMUSG00000026869.12	*Psmd5*	-0.64	4.80	27.15	1.88E-07	2.61E-05
ENSMUSG00000001415.10	*Smg5*	-0.51	5.49	26.99	2.04E-07	2.81E-05
ENSMUSG00000048186.14	*Bend7*	-0.89	3.46	26.92	2.12E-07	2.92E-05
ENSMUSG00000022687.12	*Boc*	-0.46	8.06	26.81	2.25E-07	3.07E-05
ENSMUSG00000042535.8	*Gtpbp1*	-0.52	5.59	26.80	2.26E-07	3.07E-05
ENSMUSG00000031790.8	*Mmp15*	-0.71	4.58	26.80	2.26E-07	3.07E-05
ENSMUSG00000049800.13	*Sertad2*	-0.46	5.99	26.74	2.32E-07	3.14E-05
ENSMUSG00000062328.7	*Rpl17*	-0.63	5.65	26.68	2.40E-07	3.23E-05
ENSMUSG00000057841.5	*Rpl32*	-0.53	6.39	26.53	2.59E-07	3.47E-05
ENSMUSG00000052957.7	*Gas1*	-0.82	5.34	26.48	2.66E-07	3.56E-05
ENSMUSG00000085438.1	*1700020I14Rik*	-0.42	6.87	26.24	3.01E-07	4.00E-05
ENSMUSG00000031198.4	*Fundc2*	-1.12	2.60	25.83	3.73E-07	4.91E-05
ENSMUSG00000075334.2	*Rprm*	-0.68	4.65	25.77	3.84E-07	5.04E-05
ENSMUSG00000031700.11	*Gpt2*	-0.58	4.49	25.72	3.95E-07	5.16E-05
ENSMUSG00000003534.17	*Ddr1*	-0.50	6.36	25.65	4.08E-07	5.33E-05
ENSMUSG00000021556.11	*Golm1*	-0.47	6.28	25.58	4.25E-07	5.52E-05
ENSMUSG00000006403.13	*Adamts4*	-1.34	2.07	25.58	4.24E-07	5.52E-05
ENSMUSG00000028909.17	*Ptpru*	-0.60	6.26	25.45	4.53E-07	5.86E-05
ENSMUSG00000074682.4	*Zcchc3*	-1.02	3.62	25.40	4.66E-07	6.00E-05
ENSMUSG00000040729.7	*Cep126*	-0.73	3.92	25.37	4.72E-07	6.07E-05
ENSMUSG00000030203.17	*Dusp16*	-0.53	6.23	25.33	4.83E-07	6.18E-05
ENSMUSG00000034252.14	*Senp6*	-0.40	7.54	25.27	4.99E-07	6.34E-05
ENSMUSG00000038668.14	*Lpar1*	-0.53	6.85	25.19	5.19E-07	6.57E-05
ENSMUSG00000038602.7	*Slc35f1*	-0.46	9.60	25.15	5.30E-07	6.70E-05
ENSMUSG00000030465.19	*Psd3*	-0.45	7.84	25.07	5.52E-07	6.94E-05
ENSMUSG00000032352.16	*Lrrc1*	-0.49	6.97	25.08	5.51E-07	6.94E-05
ENSMUSG00000038034.15	*Igsf8*	-0.48	6.04	25.06	5.57E-07	6.98E-05
ENSMUSG00000026749.11	*Nek6*	-0.46	6.47	25.00	5.72E-07	7.16E-05
ENSMUSG00000039046.15	*Usp6nl*	-0.43	6.76	24.94	5.90E-07	7.37E-05
ENSMUSG00000036478.8	*Btg1*	-0.60	4.85	24.83	6.26E-07	7.75E-05
ENSMUSG00000024143.14	*Rhoq*	-0.59	4.95	24.82	6.31E-07	7.80E-05
ENSMUSG00000047497.9	*Adamts12*	-1.23	2.44	24.65	6.86E-07	8.40E-05
ENSMUSG00000031627.9	*Irf2*	-0.46	5.78	24.60	7.07E-07	8.62E-05
ENSMUSG00000030351.5	*Tspan11*	-0.70	5.31	24.60	7.07E-07	8.62E-05
ENSMUSG00000025262.8	*Fam120c*	-0.41	5.86	24.47	7.55E-07	9.17E-05
ENSMUSG00000020954.16	*Strn3*	-0.35	8.21	24.38	7.93E-07	9.62E-05
ENSMUSG00000075592.9	*Nynrin*	-0.50	5.12	24.35	8.04E-07	9.73E-05
ENSMUSG00000028013.16	*Ppa2*	-0.54	4.71	24.31	8.20E-07	9.88E-05
ENSMUSG00000035168.16	*Tanc1*	-0.45	7.46	24.28	8.35E-07	0.000100
ENSMUSG00000061130.12	*Ppm1b*	-0.42	6.63	24.24	8.52E-07	0.000102
ENSMUSG00000037111.9	*Setd7*	-0.38	6.11	24.20	8.67E-07	0.000103
ENSMUSG00000019792.8	*Trmt11*	-0.45	5.67	24.19	8.71E-07	0.000104

ENSMUSG00000068040.10	*Tm9sf4*	-0.47	5.31	24.12	9.06E-07	0.000108
ENSMUSG00000054752.16	*Fsd1l*	-0.38	7.63	23.95	9.89E-07	0.000116
ENSMUSG00000041879.13	*Ipo9*	-0.36	7.29	23.94	9.92E-07	0.000116
ENSMUSG00000063810.7	*Alms1*	-0.43	6.90	23.88	1.03E-06	0.000120
ENSMUSG00000027210.20	*Meis2*	-0.43	10.69	23.83	1.05E-06	0.000123
ENSMUSG00000049916.10	*2610318N02Rik*	-0.80	4.27	23.78	1.08E-06	0.000125
ENSMUSG00000062184.11	*Hs6st2*	-0.50	8.02	23.72	1.12E-06	0.000129
ENSMUSG00000032030.16	*Cul5*	-0.38	6.59	23.71	1.12E-06	0.000129
ENSMUSG00000033059.7	*Pygb*	-0.44	5.76	23.70	1.13E-06	0.000130
ENSMUSG00000021952.15	*Xpo4*	-0.45	5.82	23.65	1.16E-06	0.000133
ENSMUSG00000037860.15	*Aim2*	-0.54	6.54	23.55	1.22E-06	0.000139
ENSMUSG00000035623.14	*Rsf1*	-0.38	7.47	23.53	1.23E-06	0.000141
ENSMUSG00000050310.8	*Rictor*	-0.40	6.81	23.43	1.30E-06	0.000148
ENSMUSG00000060206.11	*Zfp462*	-0.42	9.89	23.38	1.33E-06	0.000151
ENSMUSG00000026842.16	*Abl1*	-0.37	7.04	23.31	1.38E-06	0.000157
ENSMUSG00000022186.14	*Oxct1*	-0.37	6.63	23.26	1.42E-06	0.000160
ENSMUSG00000026923.15	*Notch1*	-0.43	7.03	23.23	1.44E-06	0.000162
ENSMUSG00000021377.14	*Dek*	-0.40	7.27	23.22	1.45E-06	0.000162
ENSMUSG00000027004.3	*Frzb*	-2.54	0.89	23.18	1.47E-06	0.000165
ENSMUSG00000024985.18	*Tcf7l2*	-0.47	6.35	23.16	1.49E-06	0.000167
ENSMUSG00000051295.7	*9630028B13Rik*	-0.88	3.33	23.11	1.53E-06	0.000171
ENSMUSG00000031340.8	*Gabre*	-1.27	2.29	23.10	1.54E-06	0.000171
ENSMUSG00000024998.17	*Plce1*	-0.53	7.28	23.05	1.58E-06	0.000174
ENSMUSG00000060862.10	*Zbtb40*	-0.64	4.95	23.04	1.58E-06	0.000175
ENSMUSG00000069769.13	*Msi2*	-0.43	9.98	23.04	1.59E-06	0.000175
ENSMUSG00000037395.15	*Rcor3*	-0.50	5.02	23.03	1.60E-06	0.000176
ENSMUSG00000030657.11	*Xylt1*	-0.44	8.08	22.93	1.68E-06	0.000184
ENSMUSG00000026893.4	*Gca*	-0.69	3.94	22.85	1.75E-06	0.000191
ENSMUSG00000068855.3	*Hist2h2ac*	-0.56	6.37	22.80	1.80E-06	0.000195
ENSMUSG00000067194.6	*Eif1ax*	-0.40	5.87	22.74	1.85E-06	0.000200
ENSMUSG00000021224.15	*Numb*	-0.35	6.62	22.69	1.90E-06	0.000204
ENSMUSG00000039323.18	*Igfbp2*	-0.44	6.23	22.65	1.94E-06	0.000209
ENSMUSG00000054256.11	*Msi1*	-0.38	7.69	22.58	2.02E-06	0.000216
ENSMUSG00000048078.16	*Tenm4*	-0.42	11.19	22.56	2.03E-06	0.000217
ENSMUSG00000048154.16	*Kmt2d*	-0.46	7.47	22.54	2.06E-06	0.000219
ENSMUSG00000031922.12	*Cep57*	-0.44	5.35	22.49	2.11E-06	0.000223
ENSMUSG00000058571.10	*Gpc6*	-0.43	10.43	22.35	2.27E-06	0.000238
ENSMUSG00000021039.9	*Snw1*	-0.45	5.38	22.29	2.34E-06	0.000245
ENSMUSG00000049119.14	*Fam110b*	-0.41	6.93	22.24	2.40E-06	0.000251
ENSMUSG00000028221.3	*Tmem55a*	-0.38	5.98	22.22	2.43E-06	0.000253
ENSMUSG00000024713.15	*Pcsk5*	-0.66	5.19	22.18	2.48E-06	0.000258
ENSMUSG00000053483.13	*Usp21*	-0.69	4.18	22.18	2.48E-06	0.000258
ENSMUSG00000079487.11	*Med12*	-0.48	5.40	22.14	2.54E-06	0.000262
ENSMUSG00000060098.11	*Prmt7*	-0.62	4.48	22.13	2.55E-06	0.000263
ENSMUSG00000061607.14	*Mdc1*	-0.38	5.82	22.11	2.58E-06	0.000265
ENSMUSG00000025420.13	*Katnal2*	-0.75	4.44	22.11	2.58E-06	0.000265
ENSMUSG00000063450.14	*Syne2*	-0.39	9.84	22.09	2.61E-06	0.000267
ENSMUSG00000030213.12	*Atf7ip*	-0.35	8.15	22.00	2.73E-06	0.000279
ENSMUSG00000032737.13	*Inppl1*	-0.50	5.07	21.99	2.74E-06	0.000280
ENSMUSG00000055320.17	*Tead1*	-0.40	8.17	21.94	2.81E-06	0.000286
ENSMUSG00000096210.1	*H1f0*	-0.49	6.56	21.87	2.92E-06	0.000296
ENSMUSG00000032198.9	*Dock6*	-0.78	3.92	21.84	2.97E-06	0.000301
ENSMUSG00000074505.5	*Fat3*	-0.37	9.67	21.83	2.98E-06	0.000301
ENSMUSG00000027878.11	*Notch2*	-0.35	7.24	21.78	3.06E-06	0.000309
ENSMUSG00000049421.13	*Zfp260*	-0.52	5.41	21.77	3.07E-06	0.000309
ENSMUSG00000020528.14	*Prpsap2*	-0.54	4.68	21.75	3.11E-06	0.000313
ENSMUSG00000021990.15	*Spata13*	-0.53	7.99	21.64	3.29E-06	0.000328
ENSMUSG00000056899.10	*Immp2l*	-0.44	6.70	21.63	3.31E-06	0.000329
ENSMUSG00000028675.12	*Pnrc2*	-0.65	4.34	21.63	3.31E-06	0.000329
ENSMUSG00000013076.17	*Amotl1*	-0.40	6.44	21.60	3.36E-06	0.000333
ENSMUSG00000041846.15	*Ppp4r3a*	-0.38	6.96	21.60	3.37E-06	0.000333
ENSMUSG00000051166.10	*Eml5*	-0.40	7.88	21.54	3.47E-06	0.000342
ENSMUSG00000069135.11	*Fgfr1op*	-0.47	4.97	21.43	3.67E-06	0.000361
ENSMUSG00000022490.6	*Ppp1r1a*	-0.64	3.92	21.37	3.79E-06	0.000371
ENSMUSG00000039197.9	*Adk*	-0.43	7.27	21.34	3.85E-06	0.000376
ENSMUSG00000046982.10	*Tshz1*	-0.50	6.19	21.34	3.85E-06	0.000376
ENSMUSG00000038900.17	*Rpl12*	-0.36	6.87	21.29	3.94E-06	0.000384
ENSMUSG00000021431.14	*Snrnp48*	-0.49	4.73	21.20	4.14E-06	0.000402
ENSMUSG00000010461.15	*Eya4*	-0.87	5.89	21.18	4.17E-06	0.000405
ENSMUSG00000052056.14	*Zfp217*	-0.50	4.78	21.17	4.20E-06	0.000407
ENSMUSG00000024827.9	*Gldc*	-0.53	5.05	21.11	4.33E-06	0.000419
ENSMUSG00000034341.17	*Wbp2*	-0.83	3.56	21.08	4.40E-06	0.000425

ENSMUSG00000000567.5	*Sox9*	-0.44	5.86	21.00	4.60E-06	0.000442
ENSMUSG00000034926.3	*Dhcr24*	-0.47	5.49	20.96	4.68E-06	0.000449
ENSMUSG00000049804.9	*Armcx4*	-0.40	6.02	20.91	4.82E-06	0.000461
ENSMUSG00000055639.16	*Dach1*	-0.63	9.72	20.86	4.95E-06	0.000472
ENSMUSG00000040044.11	*Orc3*	-0.45	5.46	20.85	4.97E-06	0.000473
ENSMUSG00000028863.13	*Meaf6*	-0.46	5.30	20.84	5.00E-06	0.000475
ENSMUSG00000046364.14	*Rpl27a*	-0.51	5.01	20.81	5.07E-06	0.000480
ENSMUSG00000022887.8	*Masp1*	-0.39	7.36	20.80	5.11E-06	0.000481
ENSMUSG00000025255.18	*Zfhx4*	-0.42	7.48	20.79	5.12E-06	0.000481
ENSMUSG00000075232.5	*Amd1*	-0.53	4.78	20.79	5.12E-06	0.000481
ENSMUSG00000032727.13	*Mier3*	-0.45	5.31	20.78	5.14E-06	0.000482
ENSMUSG00000042129.8	*Rassf4*	-0.76	3.72	20.73	5.30E-06	0.000496
ENSMUSG00000024913.16	*Lrp5*	-0.49	5.58	20.70	5.38E-06	0.000502
ENSMUSG00000030029.14	*Lrig1*	-0.63	5.91	20.69	5.40E-06	0.000503
ENSMUSG00000040481.17	*Bptf*	-0.33	7.93	20.68	5.44E-06	0.000506
ENSMUSG00000029128.12	*Rab28*	-0.44	6.34	20.62	5.60E-06	0.000520
ENSMUSG00000028532.14	*Cachd1*	-0.54	8.54	20.61	5.63E-06	0.000522
ENSMUSG00000074088.6	*Snrnp40*	-0.49	5.29	20.61	5.64E-06	0.000522
ENSMUSG00000030541.16	*Idh2*	-0.42	5.88	20.59	5.70E-06	0.000526
ENSMUSG00000045294.11	*Insig1*	-0.55	4.43	20.58	5.71E-06	0.000527
ENSMUSG00000027508.15	*Pag1*	-0.40	6.96	20.55	5.81E-06	0.000533
ENSMUSG00000022899.8	*Slc15a2*	-0.72	4.89	20.55	5.82E-06	0.000533
ENSMUSG00000027684.16	*Mecom*	-0.81	4.94	20.55	5.82E-06	0.000533
ENSMUSG00000047496.6	*Rnf152*	-0.48	5.05	20.51	5.94E-06	0.000542
ENSMUSG00000071856.10	*Mcc*	-0.49	5.06	20.48	6.02E-06	0.000547
ENSMUSG00000027496.15	*Aurka*	-0.53	4.91	20.48	6.02E-06	0.000547
ENSMUSG00000021745.13	*Ptprg*	-0.36	9.27	20.39	6.30E-06	0.000570
ENSMUSG00000028152.10	*Tspan5*	-0.35	8.07	20.33	6.51E-06	0.000588
ENSMUSG00000051177.16	*Plcb1*	-0.46	9.68	20.26	6.74E-06	0.000607
ENSMUSG00000003038.15	*Hmgn2*	-0.51	5.41	20.27	6.74E-06	0.000607
ENSMUSG00000024853.9	*Sf3b2*	-0.43	7.41	20.26	6.77E-06	0.000608
ENSMUSG00000034135.15	*Sik3*	-0.33	7.00	20.23	6.86E-06	0.000614
ENSMUSG00000024370.16	*Cdc23*	-0.37	6.23	20.23	6.87E-06	0.000614
ENSMUSG00000097462.7	*9530026P05Rik*	-1.31	2.47	20.23	6.85E-06	0.000614
ENSMUSG00000026495.8	*Efcab2*	-0.98	2.96	20.21	6.94E-06	0.000619
ENSMUSG00000051950.10	*B3glct*	-0.50	5.83	20.19	7.00E-06	0.000624
ENSMUSG00000025241.16	*Fyco1*	-0.52	4.53	20.11	7.29E-06	0.000646
ENSMUSG00000019837.8	*Gtf3c6*	-0.58	4.37	20.08	7.44E-06	0.000655
ENSMUSG00000033952.14	*Aspm*	-0.39	7.45	20.05	7.56E-06	0.000664
ENSMUSG00000021910.15	*Nisch*	-0.36	8.15	20.04	7.60E-06	0.000666
ENSMUSG00000047539.9	*Fbxo28*	-0.44	5.61	20.00	7.75E-06	0.000677
ENSMUSG00000001138.13	*Cnnm3*	-0.61	4.25	19.94	8.01E-06	0.000698
ENSMUSG00000021493.15	*Pdlim7*	-0.67	3.93	19.93	8.04E-06	0.000699
ENSMUSG00000021972.14	*Hmbox1*	-0.33	7.18	19.91	8.11E-06	0.000703
ENSMUSG00000097589.9	*Dleu2*	-0.36	8.38	19.91	8.12E-06	0.000703
ENSMUSG00000074129.13	*Rpl13a*	-0.87	2.81	19.91	8.11E-06	0.000703
ENSMUSG00000043969.4	*Emx2*	-0.65	4.94	19.77	8.76E-06	0.000753
ENSMUSG00000027933.11	*Ints3*	-0.40	5.50	19.57	9.69E-06	0.000827
ENSMUSG00000052146.15	*Rps10*	-0.64	4.77	19.57	9.70E-06	0.000827
ENSMUSG00000004233.14	*Wars2*	-0.52	4.60	19.53	9.91E-06	0.000843
ENSMUSG00000097917.2	*Gm26839*	-0.88	3.26	19.47	1.02E-05	0.000868
ENSMUSG00000031119.4	*Gpc4*	-0.44	7.77	19.42	1.05E-05	0.000889
ENSMUSG00000031521.5	*Aga*	-1.88	1.00	19.40	1.06E-05	0.000895
ENSMUSG00000022816.11	*Fstl1*	-0.54	5.48	19.33	1.10E-05	0.000922
ENSMUSG00000000628.10	*Hk2*	-0.50	4.91	19.26	1.14E-05	0.000949
ENSMUSG00000042772.15	*Smg7*	-0.35	7.11	19.25	1.15E-05	0.000953
ENSMUSG00000030067.17	*Foxp1*	-0.38	8.41	19.22	1.16E-05	0.000964
ENSMUSG00000060938.14	*Rpl26*	-0.38	5.51	19.21	1.17E-05	0.000969
ENSMUSG00000031520.6	*Vegfc*	-0.69	5.47	19.20	1.18E-05	0.000972
ENSMUSG00000046295.13	*Ankle1*	-1.04	2.64	19.20	1.18E-05	0.000972
ENSMUSG00000041235.12	*Chd7*	-0.42	8.82	19.16	1.20E-05	0.000988
ENSMUSG00000041605.16	*5730559C18Rik*	-0.75	3.99	19.15	1.21E-05	0.000993

P-value calculated with likelihood ratio tests. Adjusted P-value for multiple testing calculated using the Benjamini-Hochberg method (FDR).

Supplementary Table 3. Significantly upregulated genes by UMI RNA-seq (dKO-E vs. ctrl, FDR < 0.001)

Ensembl Mouse Gene ID	Gene symbol	logFC	logCPM	LR	PValue	FDR	Up in cKO-E
ENSMUSG00000034154.15	*Ino80*	0.62	6.83	73.53	9.92E-18	1.84E-14	
ENSMUSG00000031392.18	*Irak1*	0.80	4.40	51.85	6.00E-13	5.03E-10	
ENSMUSG00000036461.15	*Elf1*	0.62	5.18	44.77	2.21E-11	1.40E-08	
ENSMUSG00000042784.9	*Muc1*	0.88	4.22	42.53	6.97E-11	3.93E-08	
ENSMUSG00000027887.11	*Sypl2*	0.92	3.69	40.52	1.95E-10	9.64E-08	
ENSMUSG00000055897.13	*Ppp4r1l-ps*	0.52	5.34	40.14	2.36E-10	1.14E-07	
ENSMUSG00000028035.13	*Dnajb4*	0.81	5.44	40.08	2.44E-10	1.17E-07	
ENSMUSG00000035270.15	*Impg2*	0.91	3.69	39.14	3.95E-10	1.78E-07	
ENSMUSG00000042851.17	*Zc3h6*	0.90	5.25	39.12	3.98E-10	1.78E-07	
ENSMUSG00000044244.18	*Il20rb*	0.93	3.85	37.89	7.49E-10	3.14E-07	
ENSMUSG00000024330.16	*Col11a2*	1.22	2.95	37.32	1.00E-09	4.10E-07	
ENSMUSG00000080727.2	*C920021L13Rik*	0.80	3.61	34.70	3.85E-09	1.37E-06	
ENSMUSG00000069835.10	*Sat2*	1.65	1.79	30.71	2.99E-08	8.73E-06	
ENSMUSG00000028689.14	*Ccdc163*	0.90	3.29	30.34	3.63E-08	1.05E-05	
ENSMUSG00000035545.13	*Leng8*	0.53	6.62	30.26	3.78E-08	1.08E-05	
ENSMUSG00000056763.16	*Cspp1*	0.36	7.62	29.97	4.40E-08	1.23E-05	
ENSMUSG00000039191.12	*Rbpj*	0.38	7.82	29.83	4.71E-08	1.30E-05	
ENSMUSG00000059674.6	*Cdh24*	0.54	4.58	29.39	5.93E-08	1.61E-05	
ENSMUSG00000020389.19	*Cdkl3*	0.63	5.04	26.91	2.13E-07	5.01E-05	
ENSMUSG00000042567.19	*Nek10*	1.71	1.60	26.48	2.66E-07	6.17E-05	
ENSMUSG00000051427.14	*Ccdc157*	0.72	3.50	26.02	3.38E-07	7.70E-05	
ENSMUSG00000022568.16	*Scrib*	0.43	5.95	25.46	4.51E-07	0.000100	
ENSMUSG00000052139.18	*Babam2*	0.33	6.62	25.28	4.95E-07	0.000107	
ENSMUSG00000058997.7	*Vwa8*	0.37	6.35	25.27	4.99E-07	0.000107	
ENSMUSG00000020021.4	*Fgd6*	0.76	4.57	25.08	5.50E-07	0.000116	
ENSMUSG00000022557.10	*Bop1*	0.55	4.59	25.08	5.51E-07	0.000116	
ENSMUSG00000041264.16	*Uspl1*	0.38	5.88	24.98	5.79E-07	0.000121	
ENSMUSG00000036270.16	*Edc4*	0.48	5.62	23.47	1.27E-06	0.000237	
ENSMUSG00000028794.13	*A3galt2*	0.78	3.68	23.45	1.28E-06	0.000239	
ENSMUSG00000021958.4	*Pinx1*	0.36	5.47	22.60	2.00E-06	0.000346	
ENSMUSG00000026771.14	*Spopl*	0.33	6.47	22.36	2.26E-06	0.000387	
ENSMUSG00000046139.7	*Patl1*	0.41	5.38	22.06	2.64E-06	0.000434	
ENSMUSG00000020456.17	*Ogdh*	0.32	6.36	22.04	2.67E-06	0.000436	
ENSMUSG00000105961.1	*NA*	1.01	2.46	21.47	3.59E-06	0.000553	
ENSMUSG00000055538.7	*Zcchc24*	0.75	3.46	21.37	3.78E-06	0.000580	
ENSMUSG00000033009.15	*Ogfod1*	0.44	4.61	21.22	4.09E-06	0.000618	
ENSMUSG00000073236.4	*2500004C02Rik*	0.59	3.50	21.19	4.15E-06	0.000623	
ENSMUSG00000042510.7	*AA986860*	1.55	0.99	20.92	4.79E-06	0.000710	
ENSMUSG00000034303.7	*Ccdc15*	0.42	5.32	20.62	5.60E-06	0.000809	
ENSMUSG00000109408.1	*LOC102639982*	0.51	4.42	20.58	5.73E-06	0.000818	
ENSMUSG00000039137.18	*Whrn*	0.55	4.34	20.53	5.89E-06	0.000837	
ENSMUSG00000007029.16	*Vars*	0.43	5.89	20.36	6.41E-06	0.000897	
ENSMUSG00000035325.16	*Sec31a*	0.30	6.47	20.15	7.17E-06	0.000975	
ENSMUSG00000039509.8	*Nup133*	0.44	5.59	20.15	7.16E-06	0.000975	

P-value calculated with likelihood ratio tests. Adjusted P-value for multiple testing calculated using the Benjamini-Hochberg method (FDR).

Supplementary Table 4. Significantly downregulated genes by UMI RNA-seq (dKO-E vs. ctrl, FDR < 0.001)

Ensembl Mouse Gene ID	Gene symbol	logFC	logCPM	LR	PValue	FDR	Down in cKO-E
ENSMUSG00000028456.18	*Unc13b*	-1.13	6.91	225.47	5.79E-51	3.01E-46	
ENSMUSG00000040943.12	*Tet2*	-1.13	6.74	207.77	4.21E-47	1.09E-42	
ENSMUSG00000037386.15	*Rims2*	-1.01	7.99	187.98	8.77E-43	1.52E-38	
ENSMUSG00000034462.9	*Pkd2*	-1.01	5.93	180.27	4.23E-41	5.49E-37	
ENSMUSG00000022672.8	*Prkdc*	-1.21	6.05	177.75	1.50E-40	1.56E-36	
ENSMUSG00000030061.16	*Uba3*	-1.25	5.54	158.33	2.62E-36	2.27E-32	
ENSMUSG00000036023.5	*Parp2*	-1.76	3.62	153.60	2.83E-35	2.10E-31	
ENSMUSG00000032238.17	*Rora*	-0.96	7.75	143.59	4.37E-33	2.84E-29	
ENSMUSG00000036371.6	*Serbp1*	-0.98	7.25	139.95	2.73E-32	1.57E-28	
ENSMUSG00000032186.15	*Tmod2*	-0.91	6.12	134.97	3.35E-31	1.74E-27	
ENSMUSG00000073557.11	*Ppp1r12b*	-0.88	6.74	131.80	1.65E-30	7.81E-27	
ENSMUSG00000035798.14	*Zdhhc17*	-0.84	6.15	123.06	1.35E-28	5.85E-25	
ENSMUSG00000018501.17	*Ncor1*	-0.77	8.31	118.53	1.33E-27	5.29E-24	
ENSMUSG00000001127.12	*Araf*	-0.84	6.11	114.57	9.76E-27	3.38E-23	
ENSMUSG00000021027.16	*Ralgapa1*	-0.88	7.28	114.68	9.24E-27	3.38E-23	
ENSMUSG00000036377.18	*C530008M17Rik*	-0.68	8.70	104.13	1.89E-24	5.91E-21	
ENSMUSG00000025154.14	*Arhgap19*	-1.30	4.42	104.09	1.94E-24	5.91E-21	
ENSMUSG00000028080.16	*Lrba*	-0.76	6.00	103.11	3.17E-24	9.14E-21	
ENSMUSG00000039967.14	*Zfp292*	-0.68	8.26	99.18	2.31E-23	6.30E-20	
ENSMUSG00000021577.14	*Sdha*	-0.85	5.42	91.68	1.02E-21	2.64E-18	
ENSMUSG00000003360.14	*Ddx23*	-0.99	4.72	88.91	4.13E-21	1.02E-17	
ENSMUSG00000061838.7	*Suclg2*	-1.05	5.40	87.59	8.04E-21	1.90E-17	
ENSMUSG00000058729.13	*Lin9*	-0.89	5.34	87.41	8.84E-21	1.99E-17	
ENSMUSG00000035704.17	*Alg8*	-1.08	4.04	83.98	4.99E-20	1.08E-16	
ENSMUSG00000031333.7	*Abcb7*	-0.67	6.08	83.80	5.47E-20	1.14E-16	
ENSMUSG00000029104.15	*Htt*	-0.59	6.91	75.40	3.84E-18	7.67E-15	
ENSMUSG00000031290.14	*Lrch2*	-0.78	6.43	73.89	8.24E-18	1.58E-14	
ENSMUSG00000063888.6	*Rpl7l1*	-0.88	4.72	72.88	1.38E-17	2.47E-14	
ENSMUSG00000047454.12	*Gphn*	-0.67	7.46	72.68	1.53E-17	2.64E-14	
ENSMUSG00000026869.12	*Psmd5*	-0.98	4.66	72.25	1.89E-17	3.17E-14	
ENSMUSG00000035227.7	*Spcs2*	-0.82	4.77	71.70	2.50E-17	4.06E-14	
ENSMUSG00000071064.13	*Zfp827*	-0.71	7.45	71.61	2.63E-17	4.13E-14	
ENSMUSG00000028514.15	*Usp24*	-0.62	7.02	70.65	4.26E-17	6.50E-14	
ENSMUSG00000021669.15	*Col4a3bp*	-0.70	5.80	69.57	7.38E-17	1.09E-13	
ENSMUSG00000023868.15	*Pde10a*	-0.87	7.31	67.68	1.93E-16	2.78E-13	
ENSMUSG00000014426.8	*Map3k4*	-0.58	6.10	67.15	2.51E-16	3.52E-13	
ENSMUSG00000063446.4	*Plppr1*	-0.64	8.49	67.00	2.71E-16	3.71E-13	
ENSMUSG00000071757.10	*Zhx2*	-0.78	6.70	65.27	6.52E-16	8.68E-13	
ENSMUSG00000041921.16	*Metap1d*	-0.74	4.82	65.18	6.83E-16	8.86E-13	
ENSMUSG00000049421.13	*Zfp260*	-0.73	5.32	64.88	7.97E-16	1.01E-12	
ENSMUSG00000035967.15	*Ints6l*	-0.73	5.22	64.28	1.08E-15	1.34E-12	
ENSMUSG00000022636.13	*Alcam*	-0.69	8.67	64.10	1.18E-15	1.43E-12	
ENSMUSG00000038371.15	*Sbf2*	-0.53	8.38	62.77	2.33E-15	2.74E-12	
ENSMUSG00000035234.18	*Abraxas1*	-1.05	3.43	62.08	3.31E-15	3.81E-12	
ENSMUSG00000034402.3	*Kcnh5*	-0.85	6.00	60.89	6.05E-15	6.82E-12	
ENSMUSG00000039470.15	*Zdhhc2*	-0.96	4.42	59.94	9.80E-15	1.08E-11	
ENSMUSG00000040407.17	*Akap9*	-0.71	8.51	58.46	2.08E-14	2.24E-11	
ENSMUSG00000098097.7	*NA*	-0.74	5.06	58.43	2.11E-14	2.24E-11	
ENSMUSG00000039765.15	*Cc2d2a*	-0.94	4.51	58.35	2.20E-14	2.28E-11	
ENSMUSG00000059208.14	*Hnrnpm*	-0.51	7.95	58.31	2.24E-14	2.28E-11	
ENSMUSG00000097451.10	*NA*	-0.76	6.01	58.11	2.48E-14	2.47E-11	
ENSMUSG00000057914.15	*Cacnb2*	-0.70	7.54	55.52	9.26E-14	9.08E-11	
ENSMUSG00000084799.7	*Ino80dos*	-0.79	4.37	55.18	1.10E-13	1.05E-10	
ENSMUSG00000070866.4	*Zfp804a*	-0.81	5.50	55.16	1.11E-13	1.05E-10	
ENSMUSG00000025103.8	*Btbd1*	-0.59	5.88	55.10	1.15E-13	1.06E-10	
ENSMUSG00000021375.9	*Kif13a*	-0.65	6.00	54.41	1.63E-13	1.48E-10	
ENSMUSG00000037957.14	*Wdr20*	-0.53	6.21	54.03	1.98E-13	1.77E-10	
ENSMUSG00000028437.14	*Ubap1*	-0.58	5.84	53.85	2.17E-13	1.91E-10	
ENSMUSG00000020564.17	*Atxn7l1*	-0.52	7.49	53.07	3.22E-13	2.78E-10	
ENSMUSG00000032220.10	*Myo1e*	-0.71	4.57	52.12	5.22E-13	4.44E-10	
ENSMUSG00000063681.14	*Crb1*	-1.06	3.70	51.73	6.37E-13	5.25E-10	
ENSMUSG00000014164.14	*Klhl3*	-0.92	3.67	50.72	1.07E-12	8.65E-10	
ENSMUSG00000034285.15	*Nipsnap1*	-0.71	4.76	49.95	1.58E-12	1.26E-09	
ENSMUSG00000005882.18	*Uqcc1*	-0.61	5.41	49.89	1.62E-12	1.28E-09	
ENSMUSG00000064105.12	*Cnnm2*	-1.19	3.89	49.48	2.00E-12	1.55E-09	
ENSMUSG00000032498.9	*Mlh1*	-0.70	4.73	49.30	2.20E-12	1.68E-09	
ENSMUSG00000033854.10	*Kcnk10*	-0.63	7.78	49.20	2.31E-12	1.74E-09	
ENSMUSG00000020463.15	*Ppp4r3b*	-0.53	6.61	49.15	2.37E-12	1.76E-09	

ENSMUSG00000025235.8	*Bbs4*	-1.15	3.50	48.96	2.61E-12	1.91E-09
ENSMUSG00000061186.15	*Sfmbt2*	-1.64	2.56	48.81	2.82E-12	2.03E-09
ENSMUSG00000020647.10	*Ncoa1*	-0.53	7.86	48.41	3.46E-12	2.46E-09
ENSMUSG00000030982.18	*9030624J02Rik*	-0.54	5.68	47.19	6.45E-12	4.53E-09
ENSMUSG00000027204.13	*Fbn1*	-0.76	5.27	46.86	7.62E-12	5.27E-09
ENSMUSG00000019951.10	*Uhrf1bp1l*	-0.50	6.23	46.82	7.80E-12	5.33E-09
ENSMUSG00000038538.17	*Ubn2*	-0.56	7.55	46.74	8.10E-12	5.46E-09
ENSMUSG00000031684.11	*Slc10a7*	-0.53	6.73	46.18	1.08E-11	7.16E-09
ENSMUSG00000035934.16	*Pknox2*	-0.56	6.49	45.17	1.80E-11	1.18E-08
ENSMUSG00000057716.6	*Tmem178b*	-0.59	8.56	44.95	2.02E-11	1.31E-08
ENSMUSG00000021938.11	*Pspc1*	-0.46	7.33	44.89	2.09E-11	1.34E-08
ENSMUSG00000033740.17	*St18*	-0.54	6.17	44.59	2.42E-11	1.52E-08
ENSMUSG00000056900.13	*Usp13*	-0.66	4.61	44.57	2.46E-11	1.52E-08
ENSMUSG00000038070.15	*Cntln*	-0.55	6.43	44.52	2.52E-11	1.54E-08
ENSMUSG00000040044.11	*Orc3*	-0.71	5.36	44.34	2.76E-11	1.66E-08
ENSMUSG00000019894.14	*Slc6a15*	-0.85	4.27	43.63	3.96E-11	2.36E-08
ENSMUSG00000025810.9	*Nrp1*	-0.61	7.40	43.39	4.48E-11	2.64E-08
ENSMUSG00000021039.9	*Snw1*	-0.61	5.31	43.17	5.02E-11	2.93E-08
ENSMUSG00000034295.9	*Fhod3*	-0.42	7.96	42.61	6.68E-11	3.85E-08
ENSMUSG00000047539.9	*Fbxo28*	-0.54	5.56	42.56	6.86E-11	3.91E-08
ENSMUSG00000052613.16	*Pcdh15*	-0.69	6.19	42.34	7.68E-11	4.29E-08
ENSMUSG00000038248.8	*Sobp*	-0.65	7.11	42.12	8.58E-11	4.74E-08
ENSMUSG00000015189.12	*Casd1*	-0.51	5.37	41.72	1.05E-10	5.71E-08
ENSMUSG00000029782.19	*Tmem209*	-0.70	4.50	41.71	1.06E-10	5.71E-08
ENSMUSG00000032263.14	*Bckdhb*	-0.61	5.55	41.67	1.08E-10	5.80E-08
ENSMUSG00000029432.12	*Nipsnap2*	-0.65	4.59	41.52	1.17E-10	6.19E-08
ENSMUSG00000004233.14	*Wars2*	-0.71	4.52	41.36	1.27E-10	6.64E-08
ENSMUSG00000091722.1	*Siah3*	-0.53	7.36	41.14	1.42E-10	7.37E-08
ENSMUSG00000006678.6	*Pola1*	-0.55	7.53	40.94	1.57E-10	8.00E-08
ENSMUSG00000027650.12	*Tti1*	-0.73	4.49	40.95	1.56E-10	8.00E-08
ENSMUSG00000079157.4	*Fam155a*	-0.80	7.44	40.73	1.74E-10	8.79E-08
ENSMUSG00000021614.16	*Vcan*	-0.55	8.18	40.58	1.89E-10	9.43E-08
ENSMUSG00000019907.10	*Ppp1r12a*	-0.49	7.41	40.24	2.25E-10	1.10E-07
ENSMUSG00000097311.7	*NA*	-0.81	5.84	40.03	2.50E-10	1.19E-07
ENSMUSG00000085438.1	*1700020I14Rik*	-0.49	6.85	39.71	2.95E-10	1.39E-07
ENSMUSG00000056014.15	*A430033K04Rik*	-0.67	4.47	39.65	3.03E-10	1.42E-07
ENSMUSG00000027428.9	*Rbbp9*	-0.97	3.46	39.51	3.27E-10	1.52E-07
ENSMUSG00000032040.15	*Dcps*	-0.73	3.90	39.42	3.42E-10	1.57E-07
ENSMUSG00000029179.14	*Zcchc4*	-0.67	4.72	39.28	3.68E-10	1.67E-07
ENSMUSG00000034573.14	*Ptpn13*	-0.59	5.54	39.09	4.04E-10	1.79E-07
ENSMUSG00000063297.7	*Luzp2*	-0.79	5.23	38.49	5.51E-10	2.42E-07
ENSMUSG00000036672.5	*Cenpt*	-0.83	3.75	38.33	5.98E-10	2.61E-07
ENSMUSG00000021745.13	*Ptprg*	-0.39	9.27	38.22	6.32E-10	2.73E-07
ENSMUSG00000026275.13	*Ppp1r7*	-0.64	5.16	38.13	6.63E-10	2.83E-07
ENSMUSG00000031309.15	*Rps6ka3*	-0.49	6.73	38.12	6.65E-10	2.83E-07
ENSMUSG00000037852.8	*Cpe*	-0.44	7.02	37.95	7.27E-10	3.07E-07
ENSMUSG00000071753.11	*C230004F18Rik*	-0.70	5.22	37.81	7.79E-10	3.24E-07
ENSMUSG00000030231.11	*Plekha5*	-0.42	7.98	37.78	7.93E-10	3.27E-07
ENSMUSG00000052812.5	*Atad2b*	-0.46	7.39	36.95	1.21E-09	4.91E-07
ENSMUSG00000033732.10	*Sf3b3*	-0.46	6.83	36.89	1.25E-09	5.03E-07
ENSMUSG00000026058.11	*Khdrbs2*	-0.62	7.90	36.87	1.26E-09	5.04E-07
ENSMUSG00000062328.7	*Rpl17*	-0.65	5.62	36.68	1.39E-09	5.52E-07
ENSMUSG00000060862.10	*Zbtb40*	-0.65	4.92	36.60	1.45E-09	5.70E-07
ENSMUSG00000021981.9	*Cab39l*	-0.68	4.43	36.52	1.51E-09	5.84E-07
ENSMUSG00000054423.13	*Cadps*	-0.55	8.63	36.53	1.51E-09	5.84E-07
ENSMUSG00000050312.11	*Nsun3*	-0.75	4.04	36.46	1.56E-09	6.00E-07
ENSMUSG00000095041.7	*NA*	-1.94	3.40	36.10	1.87E-09	7.14E-07
ENSMUSG00000053025.13	*Sv2b*	-0.60	4.93	36.05	1.92E-09	7.28E-07
ENSMUSG00000010392.8	*Gosr1*	-0.51	5.02	35.85	2.13E-09	8.02E-07
ENSMUSG00000060579.12	*Fhit*	-0.53	5.83	35.80	2.19E-09	8.17E-07
ENSMUSG00000055296.14	*Tmem245*	-0.46	6.60	35.46	2.60E-09	9.65E-07
ENSMUSG00000032050.17	*Rdx*	-0.44	7.41	35.43	2.65E-09	9.74E-07
ENSMUSG00000018433.14	*Nol11*	-0.49	5.27	35.33	2.78E-09	1.02E-06
ENSMUSG00000056476.13	*Med12l*	-0.50	7.01	35.31	2.81E-09	1.02E-06
ENSMUSG00000041912.12	*Tdrkh*	-0.76	4.49	34.90	3.47E-09	1.25E-06
ENSMUSG00000002870.8	*Mcm2*	-0.73	6.14	34.74	3.78E-09	1.35E-06
ENSMUSG00000042156.15	*Dzip1*	-0.49	5.93	34.50	4.26E-09	1.50E-06
ENSMUSG00000017631.18	*Abr*	-0.60	6.27	34.34	4.63E-09	1.62E-06
ENSMUSG00000058325.6	*Dock1*	-0.49	7.38	34.33	4.66E-09	1.62E-06
ENSMUSG00000049550.17	*Clip1*	-0.38	7.33	34.00	5.52E-09	1.91E-06
ENSMUSG00000039456.9	*Morc3*	-0.45	5.64	33.92	5.75E-09	1.98E-06
ENSMUSG00000021846.8	*Peli2*	-0.44	6.97	33.87	5.89E-09	2.01E-06

ENSMUSG00000020952.10	*Scfd1*	-0.53	5.40	33.66	6.56E-09	2.23E-06
ENSMUSG00000034912.17	*Mdga2*	-0.64	6.94	33.64	6.62E-09	2.23E-06
ENSMUSG00000025785.5	*Exosc7*	-0.73	4.53	33.56	6.92E-09	2.32E-06
ENSMUSG00000022311.15	*Csmd3*	-0.73	6.62	33.44	7.35E-09	2.45E-06
ENSMUSG00000060923.5	*Acyp2*	-0.82	3.75	32.97	9.38E-09	3.10E-06
ENSMUSG00000038855.10	*Itpkb*	-0.82	4.23	32.80	1.02E-08	3.34E-06
ENSMUSG00000049940.7	*Pgrmc2*	-0.58	4.51	32.81	1.02E-08	3.34E-06
ENSMUSG00000039671.18	*Zmynd8*	-0.47	7.29	32.73	1.06E-08	3.44E-06
ENSMUSG00000018427.7	*Ypel2*	-0.66	4.83	32.54	1.17E-08	3.77E-06
ENSMUSG00000000976.13	*Heatr6*	-0.51	5.46	32.43	1.24E-08	3.96E-06
ENSMUSG00000009647.13	*Mcu*	-0.44	6.10	32.40	1.25E-08	3.99E-06
ENSMUSG00000041879.13	*Ipo9*	-0.40	7.28	32.06	1.50E-08	4.74E-06
ENSMUSG00000013663.7	*Pten*	-0.42	6.87	32.02	1.53E-08	4.80E-06
ENSMUSG00000075028.11	*Prdm11*	-0.47	5.62	31.99	1.55E-08	4.84E-06
ENSMUSG00000017692.8	*Rhbdl3*	-0.48	6.94	31.93	1.60E-08	4.97E-06
ENSMUSG00000039753.16	*Fbxl5*	-0.42	6.24	31.86	1.66E-08	5.13E-06
ENSMUSG00000048661.14	*Lemd3*	-0.42	6.31	31.80	1.71E-08	5.24E-06
ENSMUSG00000025332.14	*Kdm5c*	-0.67	6.06	31.76	1.74E-08	5.32E-06
ENSMUSG00000032394.6	*Igdcc3*	-0.59	5.63	31.61	1.89E-08	5.73E-06
ENSMUSG00000007908.14	*Hmgcll1*	-0.56	4.44	31.55	1.94E-08	5.86E-06
ENSMUSG00000025609.15	*Mkln1*	-0.36	7.15	31.46	2.04E-08	6.12E-06
ENSMUSG00000028760.16	*Eif4g3*	-0.39	8.59	31.36	2.14E-08	6.39E-06
ENSMUSG00000022306.8	*Zfpm2*	-0.43	8.50	31.26	2.26E-08	6.70E-06
ENSMUSG00000039968.9	*Rsbn1l*	-0.43	5.93	31.02	2.55E-08	7.53E-06
ENSMUSG00000036242.14	*3632451O06Rik*	-0.73	3.83	30.98	2.61E-08	7.65E-06
ENSMUSG00000019792.8	*Trmt11*	-0.45	5.66	30.36	3.59E-08	1.04E-05
ENSMUSG00000085816.1	*NA*	-0.41	6.75	30.26	3.78E-08	1.08E-05
ENSMUSG00000031198.4	*Fundc2*	-1.04	2.53	30.24	3.82E-08	1.08E-05
ENSMUSG00000074129.13	*Rpl13a*	-1.10	2.66	30.13	4.03E-08	1.14E-05
ENSMUSG00000005886.14	*Ncoa2*	-0.38	8.15	30.09	4.12E-08	1.16E-05
ENSMUSG00000041220.10	*Elovl6*	-0.47	7.32	29.84	4.70E-08	1.30E-05
ENSMUSG00000034252.14	*Senp6*	-0.40	7.54	29.62	5.27E-08	1.45E-05
ENSMUSG00000060938.14	*Rpl26*	-0.57	5.44	29.58	5.36E-08	1.47E-05
ENSMUSG00000005583.16	*Mef2c*	-0.64	6.22	29.34	6.08E-08	1.65E-05
ENSMUSG00000026748.13	*Plxdc2*	-0.69	4.39	29.24	6.41E-08	1.72E-05
ENSMUSG00000074505.5	*Fat3*	-0.37	9.68	29.16	6.68E-08	1.78E-05
ENSMUSG00000003452.15	*Bicd1*	-0.47	7.03	29.15	6.68E-08	1.78E-05
ENSMUSG00000039089.15	*L3mbtl3*	-0.44	6.04	29.01	7.20E-08	1.91E-05
ENSMUSG00000027238.17	*Frmd5*	-0.43	8.68	28.99	7.29E-08	1.92E-05
ENSMUSG00000045671.17	*Spred2*	-0.48	6.48	28.97	7.35E-08	1.93E-05
ENSMUSG00000041624.10	*Gucy1a2*	-0.41	6.84	28.72	8.35E-08	2.18E-05
ENSMUSG00000101609.1	*Kcnq1ot1*	-0.44	7.51	28.70	8.47E-08	2.20E-05
ENSMUSG00000086193.1	*NA*	-0.75	3.73	28.59	8.95E-08	2.31E-05
ENSMUSG00000030846.15	*Tial1*	-0.39	6.38	28.29	1.04E-07	2.68E-05
ENSMUSG00000058571.10	*Gpc6*	-0.47	10.42	28.26	1.06E-07	2.71E-05
ENSMUSG00000021072.12	*Tmx1*	-0.54	4.84	28.02	1.20E-07	3.06E-05
ENSMUSG00000052446.17	*Zfp961*	-0.54	5.03	27.98	1.23E-07	3.11E-05
ENSMUSG00000019889.10	*Ptprk*	-0.38	8.01	27.81	1.34E-07	3.37E-05
ENSMUSG00000026220.6	*Slc16a14*	-0.66	3.94	27.80	1.34E-07	3.37E-05
ENSMUSG00000032397.7	*Tipin*	-0.49	5.29	27.58	1.51E-07	3.77E-05
ENSMUSG00000040488.17	*Ltbp4*	-0.73	3.91	27.54	1.54E-07	3.81E-05
ENSMUSG00000041891.15	*Lman1*	-0.58	4.33	27.54	1.54E-07	3.81E-05
ENSMUSG00000026495.8	*Efcab2*	-1.05	2.86	27.51	1.56E-07	3.82E-05
ENSMUSG00000020019.4	*Ntn4*	-0.48	5.92	27.51	1.56E-07	3.82E-05
ENSMUSG00000027332.11	*Ivd*	-0.62	4.32	27.38	1.67E-07	4.08E-05
ENSMUSG00000052105.16	*Mtcl1*	-0.38	6.45	27.32	1.72E-07	4.18E-05
ENSMUSG00000033502.14	*Cdc14a*	-0.45	7.02	27.31	1.73E-07	4.18E-05
ENSMUSG00000020841.5	*Cpd*	-0.45	5.45	27.22	1.81E-07	4.36E-05
ENSMUSG00000034858.16	*Fam214a*	-0.51	5.93	27.21	1.82E-07	4.36E-05
ENSMUSG00000025790.14	*Slco3a1*	-0.46	6.80	27.18	1.86E-07	4.42E-05
ENSMUSG00000047747.10	*Rnf150*	-0.53	6.59	27.06	1.97E-07	4.67E-05
ENSMUSG00000075318.12	*Scn2a*	-0.83	3.73	27.02	2.01E-07	4.75E-05
ENSMUSG00000068373.14	*D430041D05Rik*	-0.38	7.08	26.78	2.28E-07	5.33E-05
ENSMUSG00000026163.17	*Sphkap*	-1.03	3.76	26.50	2.63E-07	6.13E-05
ENSMUSG00000019837.8	*Gtf3c6*	-0.65	4.32	26.38	2.80E-07	6.46E-05
ENSMUSG00000030465.19	*Psd3*	-0.41	7.86	26.37	2.82E-07	6.47E-05
ENSMUSG00000022483.16	*Col2a1*	-0.76	4.85	26.27	2.97E-07	6.78E-05
ENSMUSG00000002459.17	*Rgs20*	-0.43	7.37	25.81	3.77E-07	8.54E-05
ENSMUSG00000029863.13	*Casp2*	-0.46	5.50	25.70	3.98E-07	8.98E-05
ENSMUSG00000029705.17	*Cux1*	-0.36	8.85	25.60	4.21E-07	9.45E-05
ENSMUSG00000016520.7	*Lnx2*	-0.57	4.28	25.51	4.41E-07	9.86E-05
ENSMUSG00000038602.7	*Slc35f1*	-0.34	9.66	25.44	4.56E-07	0.000101

ENSMUSG00000034032.15	*Rpap1*	-0.56	4.25	25.43	4.58E-07	0.000101
ENSMUSG00000039345.15	*Mettl22*	-0.90	3.09	25.45	4.55E-07	0.000101
ENSMUSG00000028519.16	*Dab1*	-0.36	9.61	25.37	4.74E-07	0.000104
ENSMUSG00000055024.12	*Ep300*	-0.34	7.20	25.35	4.78E-07	0.000104
ENSMUSG00000045962.16	*Wnk1*	-0.35	8.05	25.31	4.89E-07	0.000106
ENSMUSG00000074968.11	*Ano3*	-1.99	1.14	25.15	5.30E-07	0.000114
ENSMUSG00000021314.12	*Amph*	-0.54	5.24	25.15	5.32E-07	0.000114
ENSMUSG00000056296.16	*Synpr*	-0.89	3.41	25.10	5.44E-07	0.000116
ENSMUSG00000059187.12	*Fam19a1*	-2.51	2.91	25.06	5.55E-07	0.000117
ENSMUSG00000023033.14	*Scn8a*	-0.56	6.42	25.00	5.74E-07	0.000120
ENSMUSG00000031985.9	*Gnpat*	-0.45	5.05	24.92	5.97E-07	0.000124
ENSMUSG00000040481.17	*Bptf*	-0.33	7.94	24.82	6.29E-07	0.000130
ENSMUSG00000042444.10	*Mindy2*	-0.64	4.22	24.75	6.52E-07	0.000134
ENSMUSG00000022186.14	*Oxct1*	-0.34	6.65	24.74	6.57E-07	0.000135
ENSMUSG00000049119.14	*Fam110b*	-0.37	6.94	24.66	6.85E-07	0.000140
ENSMUSG00000030257.16	*Srgap3*	-0.37	8.88	24.64	6.91E-07	0.000141
ENSMUSG00000057841.5	*Rpl32*	-0.58	6.37	24.57	7.17E-07	0.000145
ENSMUSG00000040594.19	*Ranbp17*	-0.46	6.28	24.53	7.32E-07	0.000148
ENSMUSG00000028863.13	*Meaf6*	-0.52	5.27	24.39	7.86E-07	0.000158
ENSMUSG00000050310.8	*Rictor*	-0.36	6.83	24.33	8.11E-07	0.000163
ENSMUSG00000024853.9	*Sf3b2*	-0.40	7.42	24.15	8.91E-07	0.000177
ENSMUSG00000097723.1	*NA*	-2.25	1.92	24.16	8.88E-07	0.000177
ENSMUSG00000026565.18	*Pou2f1*	-0.38	8.06	24.12	9.07E-07	0.000180
ENSMUSG00000069793.12	*Slfn9*	-1.54	3.20	24.04	9.45E-07	0.000187
ENSMUSG00000062627.9	*Mysm1*	-0.38	5.73	23.94	9.94E-07	0.000195
ENSMUSG00000037072.15	*Selenof*	-0.35	5.84	23.90	1.01E-06	0.000198
ENSMUSG00000032342.13	*Mto1*	-0.48	4.32	23.88	1.03E-06	0.000201
ENSMUSG00000037286.15	*Stag1*	-0.33	8.46	23.87	1.03E-06	0.000201
ENSMUSG00000042599.8	*Kdm7a*	-0.43	6.27	23.84	1.05E-06	0.000203
ENSMUSG00000035199.6	*Arl6ip5*	-0.61	3.95	23.79	1.07E-06	0.000207
ENSMUSG00000025969.15	*Nrp2*	-0.55	5.54	23.67	1.14E-06	0.000219
ENSMUSG00000069135.11	*Fgfr1op*	-0.45	4.97	23.63	1.17E-06	0.000223
ENSMUSG00000042225.3	*Ammecr1*	-0.40	5.47	23.62	1.17E-06	0.000223
ENSMUSG00000032228.16	*Tcf12*	-0.32	9.15	23.63	1.17E-06	0.000223
ENSMUSG00000040118.15	*Cacna2d1*	-0.45	9.85	23.62	1.17E-06	0.000223
ENSMUSG00000023088.16	*Abcc1*	-0.44	5.13	23.54	1.22E-06	0.000231
ENSMUSG00000068040.10	*Tm9sf4*	-0.39	5.33	23.49	1.25E-06	0.000236
ENSMUSG00000031284.16	*Pak3*	-0.38	8.22	23.45	1.28E-06	0.000239
ENSMUSG00000021431.14	*Snrnp48*	-0.45	4.72	23.41	1.31E-06	0.000243
ENSMUSG00000033948.3	*Zswim5*	-0.41	6.84	23.30	1.38E-06	0.000255
ENSMUSG00000042401.6	*Crtac1*	-0.70	4.60	23.31	1.38E-06	0.000255
ENSMUSG00000043940.15	*Wdfy3*	-0.32	8.62	23.26	1.41E-06	0.000259
ENSMUSG00000092837.1	*Rpph1*	-0.68	8.05	23.26	1.41E-06	0.000259
ENSMUSG00000031530.6	*Dusp4*	-0.53	4.94	23.17	1.48E-06	0.000270
ENSMUSG00000024268.15	*Celf4*	-0.61	8.28	23.13	1.51E-06	0.000274
ENSMUSG00000079056.12	*Kcnip3*	-0.59	5.13	23.05	1.58E-06	0.000285
ENSMUSG00000046668.8	*Cxxc5*	-0.50	4.97	23.05	1.58E-06	0.000285
ENSMUSG00000059602.14	*Syn3*	-0.37	6.70	22.98	1.64E-06	0.000294
ENSMUSG00000084967.1	*NA*	-0.91	2.76	22.96	1.65E-06	0.000296
ENSMUSG00000063810.7	*Alms1*	-0.40	6.92	22.95	1.67E-06	0.000296
ENSMUSG00000021420.12	*Fars2*	-0.32	7.53	22.95	1.67E-06	0.000296
ENSMUSG00000044288.6	*Cnr1*	-0.36	7.08	22.83	1.77E-06	0.000314
ENSMUSG00000087620.7	*5330434G04Rik*	-0.42	6.20	22.80	1.80E-06	0.000317
ENSMUSG00000006403.13	*Adamts4*	-1.03	2.04	22.77	1.83E-06	0.000321
ENSMUSG00000019494.14	*Cops6*	-0.50	4.59	22.72	1.88E-06	0.000329
ENSMUSG00000024064.13	*Galnt14*	-0.70	5.45	22.67	1.92E-06	0.000336
ENSMUSG00000039286.12	*Fndc3b*	-0.40	6.24	22.67	1.93E-06	0.000336
ENSMUSG00000067028.11	*Cntnap5b*	-0.63	3.53	22.62	1.98E-06	0.000343
ENSMUSG00000101011.6	*NA*	-1.98	0.65	22.53	2.07E-06	0.000357
ENSMUSG00000045867.10	*Cradd*	-0.37	5.75	22.43	2.18E-06	0.000375
ENSMUSG00000029128.12	*Rab28*	-0.42	6.34	22.35	2.27E-06	0.000388
ENSMUSG00000102252.5	*Snrpn*	-0.57	5.57	22.31	2.32E-06	0.000395
ENSMUSG00000040667.11	*Nup88*	-0.43	5.87	22.23	2.41E-06	0.000409
ENSMUSG00000025217.14	*Btrc*	-0.36	7.02	22.22	2.44E-06	0.000412
ENSMUSG00000025812.17	*Pard3*	-0.41	9.08	22.18	2.48E-06	0.000418
ENSMUSG00000029086.15	*Prom1*	-0.40	6.74	22.15	2.52E-06	0.000423
ENSMUSG00000027253.15	*Lrp4*	-0.45	6.51	22.14	2.54E-06	0.000425
ENSMUSG00000035623.14	*Rsf1*	-0.32	7.51	22.07	2.62E-06	0.000434
ENSMUSG00000022139.16	*Mbnl2*	-0.37	5.78	22.08	2.62E-06	0.000434
ENSMUSG00000025551.13	*Fgf14*	-0.59	8.15	22.07	2.63E-06	0.000434
ENSMUSG00000061731.9	*Ext1*	-0.34	8.89	22.07	2.63E-06	0.000434
ENSMUSG00000056073.16	*Grik2*	-0.35	10.25	22.06	2.64E-06	0.000434

ENSMUSG00000059146.12	*Ntrk3*	-0.33	8.77	22.04	2.66E-06	0.000436
ENSMUSG00000051177.16	*Plcb1*	-0.41	9.70	22.01	2.71E-06	0.000438
ENSMUSG00000063760.9	*Rnf217*	-0.32	6.45	22.01	2.72E-06	0.000438
ENSMUSG00000020640.10	*Itsn2*	-0.39	5.57	22.00	2.73E-06	0.000438
ENSMUSG00000027272.5	*Ubr1*	-0.34	6.82	22.02	2.70E-06	0.000438
ENSMUSG00000031320.9	*Rps4x*	-0.75	4.72	22.01	2.71E-06	0.000438
ENSMUSG00000031878.18	*Nae1*	-0.39	5.36	22.00	2.73E-06	0.000438
ENSMUSG00000045589.7	*Frrs1l*	-0.63	4.64	21.97	2.78E-06	0.000443
ENSMUSG00000002688.8	*Prkd1*	-0.43	7.76	21.95	2.79E-06	0.000443
ENSMUSG00000034593.16	*Myo5a*	-0.30	8.10	21.95	2.79E-06	0.000443
ENSMUSG00000024500.18	*Ppp2r2b*	-0.42	9.73	21.92	2.84E-06	0.000449
ENSMUSG00000033904.16	*Ccp110*	-0.40	6.33	21.85	2.95E-06	0.000465
ENSMUSG00000024981.5	*Acsl5*	-0.88	2.47	21.81	3.01E-06	0.000474
ENSMUSG00000028207.18	*Asph*	-0.39	5.77	21.80	3.03E-06	0.000475
ENSMUSG00000004633.17	*Chn2*	-0.38	7.95	21.66	3.26E-06	0.000510
ENSMUSG00000031201.17	*Brcc3*	-0.44	5.30	21.63	3.30E-06	0.000514
ENSMUSG00000006273.14	*Atp6v1b2*	-0.44	5.44	21.63	3.31E-06	0.000514
ENSMUSG00000032009.8	*Sesn3*	-0.39	5.99	21.48	3.57E-06	0.000553
ENSMUSG00000021196.14	*Pfkp*	-0.69	4.09	21.47	3.59E-06	0.000553
ENSMUSG00000048756.11	*Foxo3*	-0.38	5.85	21.30	3.93E-06	0.000596
ENSMUSG00000056899.10	*Immp2l*	-0.36	6.73	21.30	3.93E-06	0.000596
ENSMUSG00000015222.17	*Map2*	-0.39	10.01	21.30	3.94E-06	0.000596
ENSMUSG00000022332.7	*Khdrbs3*	-0.37	7.31	21.31	3.91E-06	0.000596
ENSMUSG00000028370.7	*Pappa*	-0.96	3.43	21.31	3.91E-06	0.000596
ENSMUSG00000021973.8	*Micu2*	-0.52	5.66	21.21	4.13E-06	0.000621
ENSMUSG00000020590.16	*Snx13*	-0.38	6.06	21.08	4.40E-06	0.000658
ENSMUSG00000032560.14	*Dnajc13*	-0.30	6.92	21.08	4.41E-06	0.000658
ENSMUSG00000040596.15	*Pogk*	-0.33	6.57	20.98	4.64E-06	0.000690
ENSMUSG00000071644.10	*Eef1g*	-0.40	5.83	20.91	4.80E-06	0.000710
ENSMUSG00000052726.15	*Kcnt2*	-0.36	6.25	20.87	4.90E-06	0.000723
ENSMUSG00000075334.2	*Rprm*	-0.54	4.68	20.85	4.96E-06	0.000730
ENSMUSG00000021782.14	*Dlg5*	-0.35	6.60	20.80	5.09E-06	0.000747
ENSMUSG00000053819.16	*Camk2d*	-0.44	5.55	20.80	5.11E-06	0.000747
ENSMUSG00000059482.15	*2610301B20Rik*	-0.53	4.15	20.70	5.37E-06	0.000783
ENSMUSG00000041836.10	*Ptpre*	-0.85	3.05	20.69	5.39E-06	0.000783
ENSMUSG00000046844.6	*Vat1l*	-0.51	4.22	20.66	5.47E-06	0.000793
ENSMUSG00000050812.18	*AI314180*	-0.34	7.11	20.61	5.64E-06	0.000814
ENSMUSG00000001415.10	*Smg5*	-0.44	5.51	20.59	5.68E-06	0.000817
ENSMUSG00000038349.10	*Plcl1*	-0.39	8.40	20.59	5.70E-06	0.000817
ENSMUSG00000024109.18	*Nrxn1*	-0.50	9.10	20.57	5.74E-06	0.000818
ENSMUSG00000100552.1	*NA*	-1.92	0.89	20.50	5.97E-06	0.000846
ENSMUSG00000034341.17	*Wbp2*	-0.63	3.58	20.47	6.07E-06	0.000859
ENSMUSG00000021221.15	*Dpf3*	-0.81	4.77	20.40	6.27E-06	0.000885
ENSMUSG00000039396.11	*Neil3*	-0.34	6.30	20.37	6.37E-06	0.000896
ENSMUSG00000017009.3	*Sdc4*	-1.20	1.78	20.37	6.38E-06	0.000896
ENSMUSG00000025189.8	*Cnnm1*	-0.82	3.14	20.35	6.44E-06	0.000899
ENSMUSG00000033149.16	*Phldb2*	-0.42	5.65	20.35	6.46E-06	0.000899
ENSMUSG00000042581.14	*Thsd7b*	-0.42	6.28	20.33	6.51E-06	0.000904
ENSMUSG00000074024.5	*4632427E13Rik*	-0.40	5.22	20.33	6.53E-06	0.000904
ENSMUSG00000028085.12	*Gatb*	-0.60	4.29	20.25	6.78E-06	0.000936
ENSMUSG00000020086.6	*H2afy2*	-0.38	6.69	20.23	6.87E-06	0.000946
ENSMUSG00000025241.16	*Fyco1*	-0.44	4.54	20.22	6.90E-06	0.000948
ENSMUSG00000026469.14	*Xpr1*	-0.52	7.64	20.14	7.19E-06	0.000975
ENSMUSG00000035354.9	*Uvrag*	-0.34	6.13	20.15	7.15E-06	0.000975
ENSMUSG00000051065.8	*Mb21d2*	-0.52	4.63	20.14	7.19E-06	0.000975
ENSMUSG00000061578.8	*Ksr2*	-0.54	5.34	20.13	7.22E-06	0.000976

P-value calculated with likelihood ratio tests. Adjusted P-value for multiple testing calculated using the Benjamini-Hochberg method (FDR).

Supplementary Table 5. Significantly downregulated genes in cKO-E not reversed by *Trp53* co-deletion

Ensembl Mouse Gene ID	Gene symbol	logFC	logCPM	LR	PValue	FDR	YY1 peak
ENSMUSG00000040481.17	*Bptf*	-0.33	7.94	24.82	6.29E-07	1.30E-04	■
ENSMUSG00000041879.13	*Ipo9*	-0.40	7.28	32.06	1.50E-08	4.74E-06	■
ENSMUSG00000021745.13	*Ptprg*	-0.39	9.27	38.22	6.32E-10	2.73E-07	
ENSMUSG00000074505.5	*Fat3*	-0.37	9.68	29.16	6.68E-08	1.78E-05	
ENSMUSG00000022186.14	*Oxct1*	-0.34	6.65	24.74	6.57E-07	1.35E-04	
ENSMUSG00000060938.14	*Rpl26*	-0.57	5.44	29.58	5.36E-08	1.47E-05	■
ENSMUSG00000035623.14	*Rsf1*	-0.32	7.51	22.07	2.62E-06	4.34E-04	
ENSMUSG00000045962.16	*Wnk1*	-0.35	8.05	25.31	4.89E-07	1.06E-04	■
ENSMUSG00000037286.15	*Stag1*	-0.33	8.46	23.87	1.03E-06	2.01E-04	■
ENSMUSG00000050310.8	*Rictor*	-0.36	6.83	24.33	8.11E-07	1.63E-04	■
ENSMUSG00000034252.14	*Senp6*	-0.40	7.54	29.62	5.27E-08	1.45E-05	■
ENSMUSG00000019907.10	*Ppp1r12a*	-0.49	7.41	40.24	2.25E-10	1.10E-07	■
ENSMUSG00000049119.14	*Fam110b*	-0.37	6.94	24.66	6.85E-07	1.40E-04	
ENSMUSG00000085438.1	*1700020I14Rik*	-0.49	6.85	39.71	2.95E-10	1.39E-07	■
ENSMUSG00000021782.14	*Dlg5*	-0.35	6.60	20.80	5.09E-06	7.47E-04	
ENSMUSG00000055024.12	*Ep300*	-0.34	7.20	25.35	4.78E-07	1.04E-04	■
ENSMUSG00000063810.7	*Alms1*	-0.40	6.92	22.95	1.67E-06	2.96E-04	■
ENSMUSG00000024853.9	*Sf3b2*	-0.40	7.42	24.15	8.91E-07	1.77E-04	■
ENSMUSG00000058571.10	*Gpc6*	-0.47	10.42	28.26	1.06E-07	2.71E-05	
ENSMUSG00000056899.10	*Immp2l*	-0.36	6.73	21.30	3.93E-06	5.96E-04	
ENSMUSG00000025609.15	*Mkln1*	-0.36	7.15	31.46	2.04E-08	6.12E-06	■
ENSMUSG00000029128.12	*Rab28*	-0.42	6.34	22.35	2.27E-06	3.88E-04	■
ENSMUSG00000047539.9	*Fbxo28*	-0.54	5.56	42.56	6.86E-11	3.91E-08	■
ENSMUSG00000039753.16	*Fbxl5*	-0.42	6.24	31.86	1.66E-08	5.13E-06	
ENSMUSG00000030846.15	*Tial1*	-0.39	6.38	28.29	1.04E-07	2.68E-05	■
ENSMUSG00000030465.19	*Psd3*	-0.41	7.86	26.37	2.82E-07	6.47E-05	
ENSMUSG00000019792.8	*Trmt11*	-0.45	5.66	30.36	3.59E-08	1.04E-05	■
ENSMUSG00000040044.11	*Orc3*	-0.71	5.36	44.34	2.76E-11	1.66E-08	■
ENSMUSG00000021039.9	*Snw1*	-0.61	5.31	43.17	5.02E-11	2.93E-08	■
ENSMUSG00000028863.13	*Meaf6*	-0.52	5.27	24.39	7.86E-07	1.58E-04	■
ENSMUSG00000051177.16	*Plcb1*	-0.41	9.70	22.01	2.71E-06	4.38E-04	
ENSMUSG00000038602.7	*Slc35f1*	-0.34	9.66	25.44	4.56E-07	1.01E-04	
ENSMUSG00000068040.10	*Tm9sf4*	-0.39	5.33	23.49	1.25E-06	2.36E-04	■
ENSMUSG00000069135.11	*Fgfr1op*	-0.45	4.97	23.63	1.17E-06	2.23E-04	■
ENSMUSG00000037957.14	*Wdr20*	-0.53	6.21	54.03	1.98E-13	1.77E-10	■
ENSMUSG00000033732.10	*Sf3b3*	-0.46	6.83	36.89	1.25E-09	5.03E-07	■
ENSMUSG00000021431.14	*Snrnp48*	-0.45	4.72	23.41	1.31E-06	2.43E-04	■
ENSMUSG00000059208.14	*Hnrnpm*	-0.51	7.95	58.31	2.24E-14	2.28E-11	■
ENSMUSG00000055296.14	*Tmem245*	-0.46	6.60	35.46	2.60E-09	9.65E-07	■
ENSMUSG00000048661.14	*Lemd3*	-0.42	6.31	31.80	1.71E-08	5.24E-06	■
ENSMUSG00000028514.15	*Usp24*	-0.62	7.02	70.65	4.26E-17	6.50E-14	■
ENSMUSG00000001415.10	*Smg5*	-0.44	5.51	20.59	5.68E-06	8.17E-04	■
ENSMUSG00000027253.15	*Lrp4*	-0.45	6.51	22.14	2.54E-06	4.25E-04	
ENSMUSG00000005882.18	*Uqcc1*	-0.61	5.41	49.89	1.62E-12	1.28E-09	■
ENSMUSG00000004233.14	*Wars2*	-0.71	4.52	41.36	1.27E-10	6.64E-08	■
ENSMUSG00000025241.16	*Fyco1*	-0.44	4.54	20.22	6.90E-06	9.48E-04	■
ENSMUSG00000049421.13	*Zfp260*	-0.73	5.32	64.88	7.97E-16	1.01E-12	■
ENSMUSG00000025103.8	*Btbd1*	-0.59	5.88	55.10	1.15E-13	1.06E-10	■
ENSMUSG00000057841.5	*Rpl32*	-0.58	6.37	24.57	7.17E-07	1.45E-04	
ENSMUSG00000052812.5	*Atad2b*	-0.46	7.39	36.95	1.21E-09	4.91E-07	■
ENSMUSG00000047454.12	*Gphn*	-0.67	7.46	72.68	1.53E-17	2.64E-14	■
ENSMUSG00000036377.18	*C530008M17Rik*	-0.68	8.70	104.13	1.89E-24	5.91E-21	
ENSMUSG00000032050.17	*Rdx*	-0.44	7.41	35.43	2.65E-09	9.74E-07	
ENSMUSG00000015189.12	*Casd1*	-0.51	5.37	41.72	1.05E-10	5.71E-08	■
ENSMUSG00000031684.11	*Slc10a7*	-0.53	6.73	46.18	1.08E-11	7.16E-09	■
ENSMUSG00000021938.11	*Pspc1*	-0.46	7.33	44.89	2.09E-11	1.34E-08	■
ENSMUSG00000019889.10	*Ptprk*	-0.38	8.01	27.81	1.34E-07	3.37E-05	
ENSMUSG00000039968.9	*Rsbn1l*	-0.43	5.93	31.02	2.55E-08	7.53E-06	■
ENSMUSG00000019951.10	*Uhrf1bp1l*	-0.50	6.23	46.82	7.80E-12	5.33E-09	■
ENSMUSG00000034573.14	*Ptpn13*	-0.59	5.54	39.09	4.04E-10	1.79E-07	
ENSMUSG00000020463.15	*Ppp4r3b*	-0.53	6.61	49.15	2.37E-12	1.76E-09	■
ENSMUSG00000019837.8	*Gtf3c6*	-0.65	4.32	26.38	2.80E-07	6.46E-05	■
ENSMUSG00000031333.7	*Abcb7*	-0.67	6.08	83.80	5.47E-20	1.14E-16	■
ENSMUSG00000029104.15	*Htt*	-0.59	6.91	75.40	3.84E-18	7.67E-15	■
ENSMUSG00000028080.16	*Lrba*	-0.76	6.00	103.11	3.17E-24	9.14E-21	■

ENSMUSG00000039089.15	*L3mbtl3*	-0.44	6.04	29.01	7.20E-08	1.91E-05
ENSMUSG00000021375.9	*Kif13a*	-0.65	6.00	54.41	1.63E-13	1.48E-10
ENSMUSG00000031290.14	*Lrch2*	-0.78	6.43	73.89	8.24E-18	1.58E-14
ENSMUSG00000033502.14	*Cdc14a*	-0.45	7.02	27.31	1.73E-07	4.18E-05
ENSMUSG00000058325.6	*Dock1*	-0.49	7.38	34.33	4.66E-09	1.62E-06
ENSMUSG00000029432.12	*Nipsnap2*	-0.65	4.59	41.52	1.17E-10	6.19E-08
ENSMUSG00000035798.14	*Zdhhc17*	-0.84	6.15	123.06	1.35E-28	5.85E-25
ENSMUSG00000014426.8	*Map3k4*	-0.58	6.10	67.15	2.51E-16	3.52E-13
ENSMUSG00000062328.7	*Rpl17*	-0.65	5.62	36.68	1.39E-09	5.52E-07
ENSMUSG00000029179.14	*Zcchc4*	-0.67	4.72	39.28	3.68E-10	1.67E-07
ENSMUSG00000060862.10	*Zbtb40*	-0.65	4.92	36.60	1.45E-09	5.70E-07
ENSMUSG00000041220.10	*Elovl6*	-0.47	7.32	29.84	4.70E-08	1.30E-05
ENSMUSG00000001127.12	*Araf*	-0.84	6.11	114.57	9.76E-27	3.38E-23
ENSMUSG00000038070.15	*Cntln*	-0.55	6.43	44.52	2.52E-11	1.54E-08
ENSMUSG00000035934.16	*Pknox2*	-0.56	6.49	45.17	1.80E-11	1.18E-08
ENSMUSG00000006678.6	*Pola1*	-0.55	7.53	40.94	1.57E-10	8.00E-08
ENSMUSG00000027332.11	*Ivd*	-0.62	4.32	27.38	1.67E-07	4.08E-05
ENSMUSG00000021981.9	*Cab39l*	-0.68	4.43	36.52	1.51E-09	5.84E-07
ENSMUSG00000026275.13	*Ppp1r7*	-0.64	5.16	38.13	6.63E-10	2.83E-07
ENSMUSG00000021577.14	*Sdha*	-0.85	5.42	91.68	1.02E-21	2.64E-18
ENSMUSG00000018501.17	*Ncor1*	-0.77	8.31	118.53	1.33E-27	5.29E-24
ENSMUSG00000075334.2	*Rprm*	-0.54	4.68	20.85	4.96E-06	7.30E-04
ENSMUSG00000020564.17	*Atxn7l1*	-0.52	7.49	53.07	3.22E-13	2.78E-10
ENSMUSG00000028437.14	*Ubap1*	-0.58	5.84	53.85	2.17E-13	1.91E-10
ENSMUSG00000021027.16	*Ralgapa1*	-0.88	7.28	114.68	9.24E-27	3.38E-23
ENSMUSG00000034285.15	*Nipsnap1*	-0.71	4.76	49.95	1.58E-12	1.26E-09
ENSMUSG00000021669.15	*Col4a3bp*	-0.70	5.80	69.57	7.38E-17	1.09E-13
ENSMUSG00000022636.13	*Alcam*	-0.69	8.67	64.10	1.18E-15	1.43E-12
ENSMUSG00000039967.14	*Zfp292*	-0.68	8.26	99.18	2.31E-23	6.30E-20
ENSMUSG00000071064.13	*Zfp827*	-0.71	7.45	71.61	2.63E-17	4.13E-14
ENSMUSG00000034402.3	*Kcnh5*	-0.85	6.00	60.89	6.05E-15	6.82E-12
ENSMUSG00000035227.7	*Spcs2*	-0.82	4.77	71.70	2.50E-17	4.06E-14
ENSMUSG00000025810.9	*Nrp1*	-0.61	7.40	43.39	4.48E-11	2.64E-08
ENSMUSG00000063446.4	*Plppr1*	-0.64	8.49	67.00	2.71E-16	3.71E-13
ENSMUSG00000041921.16	*Metap1d*	-0.74	4.82	65.18	6.83E-16	8.86E-13
ENSMUSG00000029782.19	*Tmem209*	-0.70	4.50	41.71	1.06E-10	5.71E-08
ENSMUSG00000032498.9	*Mlh1*	-0.70	4.73	49.30	2.20E-12	1.68E-09
ENSMUSG00000032263.14	*Bckdhb*	-0.61	5.55	41.67	1.08E-10	5.80E-08
ENSMUSG00000063888.6	*Rpl7l1*	-0.88	4.72	72.88	1.38E-17	2.47E-14
ENSMUSG00000079056.12	*Kcnip3*	-0.59	5.13	23.05	1.58E-06	2.85E-04
ENSMUSG00000027650.12	*Tti1*	-0.73	4.49	40.95	1.56E-10	8.00E-08
ENSMUSG00000035967.15	*Ints6l*	-0.73	5.22	64.28	1.08E-15	1.34E-12
ENSMUSG00000036371.6	*Serbp1*	-0.98	7.25	139.95	2.73E-32	1.57E-28
ENSMUSG00000002870.8	*Mcm2*	-0.73	6.14	34.74	3.78E-09	1.35E-06
ENSMUSG00000034341.17	*Wbp2*	-0.63	3.58	20.47	6.07E-06	8.59E-04
ENSMUSG00000027428.9	*Rbbp9*	-0.97	3.46	39.51	3.27E-10	1.52E-07
ENSMUSG00000003360.14	*Ddx23*	-0.99	4.72	88.91	4.13E-21	1.02E-17
ENSMUSG00000056014.15	*A430033K04Rik*	-0.67	4.47	39.65	3.03E-10	1.42E-07
ENSMUSG00000052613.16	*Pcdh15*	-0.69	6.19	42.34	7.68E-11	4.29E-08
ENSMUSG00000074129.13	*Rpl13a*	-1.10	2.66	30.13	4.03E-08	1.14E-05
ENSMUSG00000064105.12	*Cnnm2*	-1.19	3.89	49.48	2.00E-12	1.55E-09
ENSMUSG00000039470.15	*Zdhhc2*	-0.96	4.42	59.94	9.80E-15	1.08E-11
ENSMUSG00000032040.15	*Dcps*	-0.73	3.90	39.42	3.42E-10	1.57E-07
ENSMUSG00000026495.8	*Efcab2*	-1.05	2.86	27.51	1.56E-07	3.82E-05
ENSMUSG00000040943.12	*Tet2*	-1.13	6.74	207.77	4.21E-47	1.09E-42
ENSMUSG00000032220.10	*Myo1e*	-0.71	4.57	52.12	5.22E-13	4.44E-10
ENSMUSG00000035704.17	*Alg8*	-1.08	4.04	83.98	4.99E-20	1.08E-16
ENSMUSG00000032238.17	*Rora*	-0.96	7.75	143.59	4.37E-33	2.84E-29
ENSMUSG00000027204.13	*Fbn1*	-0.76	5.27	46.86	7.62E-12	5.27E-09
ENSMUSG00000039765.15	*Cc2d2a*	-0.94	4.51	58.35	2.20E-14	2.28E-11
ENSMUSG00000022672.8	*Prkdc*	-1.21	6.05	177.75	1.50E-40	1.56E-36
ENSMUSG00000035234.18	*Abraxas1*	-1.05	3.43	62.08	3.31E-15	3.81E-12
ENSMUSG00000034462.9	*Pkd2*	-1.01	5.93	180.27	4.23E-41	5.49E-37
ENSMUSG00000030061.16	*Uba3*	-1.25	5.54	158.33	2.62E-36	2.27E-32
ENSMUSG00000063681.14	*Crb1*	-1.06	3.70	51.73	6.37E-13	5.25E-10
ENSMUSG00000058729.13	*Lin9*	-0.89	5.34	87.41	8.84E-21	1.99E-17
ENSMUSG00000031198.4	*Fundc2*	-1.04	2.53	30.24	3.82E-08	1.08E-05
ENSMUSG00000025154.14	*Arhgap19*	-1.30	4.42	104.09	1.94E-24	5.91E-21

ENSMUSG00000006403.13	*Adamts4*	-1.03	2.04	22.77	1.83E-06	3.21E-04

P-value calculated with likelihood ratio tests. Adjusted P-value for multiple testing calculated using the Benjamini-Hochberg method (FDR).

Supplementary Table 6. Mouse strains

**Mouse**	**ID Number**
B6.129(C3)-*Ino80*$^{tm1.1Jland}$/J<br>*Ino80*^{fl}	JAX# 027920
B6.129P2-*Gt(ROSA)26Sor*$^{tm1(DTA)Lky}$/J<br>*ROSA*^{DTA}	JAX# 009669
B6N.129S6-*Gt(ROSA)26Sor*$^{tm1(CAG\text{-}tdTomato*,\text{-}EGFP*)Ees}$/J<br>*ROSA*$^{nT\text{-}nG}$	JAX# 023537
B6.129P2-*Trp53*^{tm1Brn}/J<br>*p53*^{fl}	JAX# 008462
B6.129S2-*Emx1*$^{tm1(cre)Krj}$/J<br>*Emx1*$^{IRES\text{-}Cre}$	JAX# 005628
B6.129P2(Cg)-*Foxg1*$^{tm1(cre)Skm}$/J<br>*Foxg1*^{Cre}	JAX# 006084
Neurod6$^{tm1(cre)Kan}$<br>*Neurod6*^{Cre}	MGI# 2668659
FVB-*Tg(GFAP-cre)25Mes/J*<br>*Tg(hGFAP-Cre)*	JAX# 004600
STOCK *Brca2*^{tm1Brn}*/Nci*	NCI# 01XB9

Supplementary Table 7. Genotyping primers

Gene Target	Oligo Name	Sequence (5' - 3')	PCR product
Cre	Cre-F	TCGATGCAACGAGTGATGAG	500 bp
	Cre-R	TTCGGCTATACGTAACAGGG	
Ino80^{fl}	Ino80-F	GCACTTCCTGGTTTTGCTGT	WT = 290 bp<br>floxed = 350 bp
	Ino80-R	CACTGACTGGCGTGTTCAGA	
Ino80^{fl}	Ino80-P1	CTGGATGTGAAGGGAGAAGG	WT = 416 bp<br>floxed = 482 bp<br>deleted allele = 352 bp
	Ino80-P2	CATCTCTCCAGCCAGCACACT	
	Ino80-P3	TGCCACTCTACCTGCATCTG	
DTA	DTA-F	CGACCTGCAGGTCCTCG	650 bp
	DTA-R	CTCGAGTTTGTCCAATTATGTCAC	
Trp53^{fl}	p53-F	GGTTAAACCCAGCTTGACCA	WT = 270 bp<br>floxed = 390 bp
	p53-R	GGAGGCAGAGACAGTTGGAG	
Brca2^{fl}	Brca2-F	GGCTGTCTTAGAACTTAGGCTG	WT = 298 bp<br>floxed = 376 bp
	Brca2-R	CTCCACACATACATCATGTGTC	

Supplementary Table 8. Primary antibodies

Primary Antibody	Company and product number	Dilution	Notes
Rabbit anti-INO80	Qiu et al., 2016, kind gift of J. Landry	WB, 1:1000	
Rabbit anti-INO80	Proteintech 18810-1-AP	WB, 1:500	
Rabbit anti-INO80	Abcam ab105451	WB, 1:1000	
Rabbit anti-GAPDH	Santa Cruz Biotechnology sc-25778	WB, 1:1500	
Chicken anti-MAP2	Novus Biologicals NB300-213	IF, 1:2000	
Rabbit anti-GFP	Thermo Fisher A-11122	IF, 1:250	
Chicken anti-GFP	Abcam ab13970	IF, 1:2000	
Rat anti-BCL11B	Abcam ab18465	IF, 1:500	
Mouse anti-TLE4	Santa Cruz Biotechnology sc-365406	IF, 1:250	
Rat anti-L1-CAM	EMD Millipore MAB5272	IF, 1:500	
Rat anti-EOMES	Thermo Fisher 14-4875-80	IF, 1:500	
Rabbit anti-SOX2	Millipore/Chemicon AB5603	IF, 1:2000	
Goat anti-SOX2	Santa Cruz Biotechnology sc-17320	IF, 1:250	
Rabbit anti-CC3	Cell Signaling 9661S	IF, 1:500	
Rabbit anti-CC3-Alexa555	Cell Signaling 9604S	IF, 1:100	Alexa555-labeled
Rabbit anti-pHH3-Alexa647	Cell Signaling 3458S	IF, 1:400	Alexa647-labeled
Rabbit anti-TRP53	Leica P53-CM5P-L	IF, 1:500	
Rat anti-F4/80	Abcam ab6640	IF, 1:500	
Rabbit anti-pKAP1	Bethyl Laboratories A300-767A	IF, 1:200	
Rat anti-HA	Sigma-Aldrich 11867423001	IF, 1:250	
Hamster anti-Myc	Absolute Antibody Ab00100-22.0	IF, 1:1000	
Rabbit anti-γH2AX	Cell Signaling 9718S	IF, 1:250	
Chicken anti-RBFOX3	EMD Millipore ABN91	IF, 1:2000	
Rabbit anti-NEUROG2	Cell Signaling 13144S	IF, 1:250	antigen retrieval
Rabbit anti-SATB2	Abcam ab92446	IF, 1:500	
Rabbit anti-LHX2	EMD Millipore ABE1402	IF, 1:2000	
Rabbit anti-TP53BP1	Novus Biologicals NB100-304SS	IF, 1:1000	
Mouse anti-γH2AX	Millipore Sigma 05-636	IF, 1:500	

Supplementary Table 9. Secondary antibodies

Secondary Antibody	Company	Dilution
AlexaFluor 488 AffiniPure Donkey anti-Rabbit IgG (H+L)	Jackson ImmunoResearch Labs	1:250
AlexaFluor 488 AffiniPure Donkey anti-Rat IgG (H+L)	Jackson ImmunoResearch Labs	1:250
AlexaFluor 488 AffiniPure Donkey anti-Goat IgG (H+L)	Jackson ImmunoResearch Labs	1:250
AlexaFluor 594 AffiniPure Donkey anti-Mouse IgG (H+L)	Jackson ImmunoResearch Labs	1:250
Cy3 AffiniPure Donkey anti-Rabbit IgG (H+L)	Jackson ImmunoResearch Labs	1:250
Cy3 AffiniPure Donkey anti-Rat IgG (H+L)	Jackson ImmunoResearch Labs	1:250
AlexaFluor 647 AffiniPure Donkey anti-Goat IgG (H+L)	Jackson ImmunoResearch Labs	1:250
AlexaFluor 647 Affinipure Donkey anti-Rabbit IgG (H+L)	Jackson ImmunoResearch Labs	1:250
AlexaFluor 647 Affinipure Donkey anti-Rat IgG (H+L)	Jackson ImmunoResearch Labs	1:250
AlexaFluor 647 Affinipure Donkey anti-Chicken IgG (H+L)	Jackson ImmunoResearch Labs	1:250

Supplementary Table 10. Primers and probes for ddPCR

Gene Target	Primer_1 / Primer_2	Probe (5' - 3')
Eda2r	GCATCTACCTTCACTAAGCTCA<br>GTTCTACCGAAAGACACGCAT	/56-FAM/TGCATCCCA /Zen/TGTACAAAGCAGACTCC/3IABkFQ/
Pvt1	GCCACTGCCAATGTCTGT<br>CACTGAAAACAAGGACCGAAAC	/56-FAM/TCCAGGTAG /Zen/CCCGAGAGATGACA/3IABkFQ/
Ano3	CGAAGAAAACTGTCCCTCCAT<br>GTCTTCATGTGTCCTCTATGTGA	/56-FAM/AGCTGTCGT /Zen/TGAGTCTCTGCAGTG/3IABkFQ/
Ino80	CTCTCATACTCTGCACTTCTGTC<br>GCTACCTCCTCACTTCGTTG	/56-FAM/CTGCGGTGA /ZEN/CTCGTGGACTGG/3IABkFQ/
Srp72	CTCTCCTCATCATAGTCGTCCT<br>CTGAAGGAGCTTTATGGACAAGT	/5HEX/CCAAGCACT /Zen/CATCGTAGCGTTCCA/3IABkFQ/
