# Supplementary material for: Symmetric neural progenitor divisions require chromatin-mediated homologous recombination DNA repair by Ino80
Source: Nat Commun. 2020 Jul 31;11:3839. doi: 10.1038/s41467-020-17551-4 (PMC7395731; doi:10.1038/s41467-020-17551-4)
Supplement: Supplementary file 2 — Reporting Summary [file 41467_2020_17551_MOESM2_ESM.pdf]

nature research

Corresponding author(s): Kenneth Y. Kwan

Last updated by author(s): Jun 26, 2020

Reporting Summary

Nature Research wishes to improve the reproducibility of the work that we publish. This form provides structure for consistency and transparency in reporting. For further information on Nature Research policies, seeAuthors & Referees and theEditorial Policy Checklist .

Statistics

For all statistical analyses, confirm that the following items are present in the figure legend, table legend, main text, or Methods section.

n/a	Confirmed	
☐	☒	The exact sample size (*n*) for each experimental group/condition, given as a discrete number and unit of measurement
☐	☒	A statement on whether measurements were taken from distinct samples or whether the same sample was measured repeatedly
☐	☒	The statistical test(s) used AND whether they are one- or two-sided<br>*Only common tests should be described solely by name; describe more complex techniques in the Methods section.*
☐	☒	A description of all covariates tested
☐	☒	A description of any assumptions or corrections, such as tests of normality and adjustment for multiple comparisons
☐	☒	A full description of the statistical parameters including central tendency (e.g. means) or other basic estimates (e.g. regression coefficient) AND variation (e.g. standard deviation) or associated estimates of uncertainty (e.g. confidence intervals)
☐	☒	For null hypothesis testing, the test statistic (e.g. *F*, *t*, *r*) with confidence intervals, effect sizes, degrees of freedom and *P* value noted<br>*Give P values as exact values whenever suitable.*
☒	☐	For Bayesian analysis, information on the choice of priors and Markov chain Monte Carlo settings
☒	☐	For hierarchical and complex designs, identification of the appropriate level for tests and full reporting of outcomes
☐	☒	Estimates of effect sizes (e.g. Cohen's *d*, Pearson's *r*), indicating how they were calculated

Our web collection on statistics for biologists contains articles on many of the points above.

Software and code

Policy information about availability of computer code

Data collection	No software was used for data collection.
Data analysis	RNA-seq data were subject to quality control check using FastQC (v0.11.5) (https://www.bioinformatics.babraham.ac.uk/projects/download.html#fastqc). Adapters were trimmed using cutadapt (version 1.13) (http://cutadapt.readthedocs.io/en/stable/guide.html). Processed reads were aligned to the GENCODE GRCm38/mm10 reference genome (https://www.gencodegenes.org/mouse_releases/current.html) with STAR (v2.5.2a) and deduplicated according to UMI using UMI-tools (v0.5.3). Read counts were obtained with htseq-count (v0.6.1p1) with intersection-nonempty mode. Differential expression was determined with edgeR (v3.20.9). The p-value was calculated with likelihood ratio tests and the adjusted p-value for multiple testing was calculated using the Benjamini-Hochberg procedure, which controls false discovery rate. For ATAC-seq data analysis, after trimming adapter using cutadapt, reads were aligned to the mouse reference genome (GRCh38/mm10) using BWA (v0.7.15) with default settings. Low quality, mitochondrial, and duplicate reads were removed using a combination of SAMTools (v1.9) and Picard's MarckDuplicates program (v2.4.1). ATAC-seq peaks were called using Macs2(v2.1.2) with the parameters: --nomodel --extsize 200 --shift -100, and blacklisted regions were excluded. Other software: Q-Capture Pro (7), GraphPad Prism (7,8), Adobe Photoshop (CC 2018), ImageJ (1.52p).

For manuscripts utilizing custom algorithms or software that are central to the research but not yet described in published literature, software must be made available to editors/reviewers. We strongly encourage code deposition in a community repository (e.g. GitHub). See the Nature Research guidelines for submitting code & software for further information.

Data

Policy information about availability of data

All manuscripts must include a data availability statement. This statement should provide the following information, where applicable:

- Accession codes, unique identifiers, or web links for publicly available datasets
- A list of figures that have associated raw data
- A description of any restrictions on data availability

DATA AVAILABILITY
The RNA-seq and ATAC-seq data that support the findings of this study have been deposited to NCBI GEO with the accession number GSE153062 [https://www.ncbi.nlm.nih.gov/geo/query/acc.cgi?acc=GSE153062]. Data from publicly available sources: GRCm38/mm10 reference genome [https://www.ncbi.nlm.nih.gov/assembly/GCF_000001635.26], ENCODE [https://www.encodeproject.org/ ENCSR160IIN, ENCSR647QBV, ENCSR970EWM, ENCSR185LWM, ENCSR752RGN, ENCSR080EVZ, ENCSR362AIZ], and EnhancerAtlas 2.0 [http://www.enhanceratlas.org/ E14.5 brain].

Field-specific reporting

Please select the one below that is the best fit for your research. If you are not sure, read the appropriate sections before making your selection.

☒ Life sciences ☐ Behavioural & social sciences ☐ Ecological, evolutionary & environmental sciences

For a reference copy of the document with all sections, see nature.com/documents/nr-reporting-summary-flat.pdf

Life sciences study design

All studies must disclose on these points even when the disclosure is negative.

Sample size	No statistical methods were used to predetermine sample size. Sample size was at least three animals per group for each analysis and consistent with recent literature in the field, including Tedeschi, et al., Nature Communications, 2020, Popovitchenko, et al., Nature Communications, 2020, and Shi, et al., Nature Communications, 2019.
Data exclusions	For RNA-seq and ATAC-seq data, low quality, mitochondrial, and duplicate reads were removed using pre-established, default settings. No data were excluded from any other analysis.
Replication	The reproducibility of our conclusions was verified by replication, and at least 3 independent replicates were used for each experiment.
Randomization	Randomization was not relevant to this study. Experimental groups were based on genotype.
Blinding	Blinding was not used for this study. The mutant phenotypes were robust and obvious at both macroscopic and microscopic levels of analysis. True blinding was not possible.

Reporting for specific materials, systems and methods

We require information from authors about some types of materials, experimental systems and methods used in many studies. Here, indicate whether each material, system or method listed is relevant to your study. If you are not sure if a list item applies to your research, read the appropriate section before selecting a response.

Materials & experimental systems

n/a	Involved in the study	
☐	☒	Antibodies
☒	☐	Eukaryotic cell lines
☒	☐	Palaeontology
☐	☒	Animals and other organisms
☒	☐	Human research participants
☒	☐	Clinical data

Methods

n/a	Involved in the study	
☒	☐	ChIP-seq
☒	☐	Flow cytometry
☒	☐	MRI-based neuroimaging

Antibodies

Antibodies used

Primary Antibodies:
Chicken anti-MAP2, Novus Biologicals NB300-213, 1:2000
Rabbit anti-GFP, Thermo Fisher A-11122, 1:250
Chicken anti-GFP, Abcam ab13970, 1:2000
Rat anti-BCL11B, Abcam ab18465, 1:500
Mouse anti-TLE4, Santa Cruz Biotechnology sc-365406, 1:250

Rat anti-L1-CAM, EMD Millipore MAB5272, 1:500
Rat anti-EOMES, Thermo Fisher 14-4875-80, 1:500
Rabbit anti-SOX2, Millipore/Chemicon AB5603, 1:2000
Goat anti-SOX2, Santa Cruz Biotechnology sc-17320, 1:250
Rabbit anti-CC3, Cell Signaling 9661S, 1:500
Rabbit anti-CC3-Alexa555, Cell Signaling 9604S, 1:100 - Alexa555-labeled
Rabbit anti-pHH3-Alexa647, Cell Signaling 3458S, 1:400 - Alexa647-labeled
Rabbit anti-TRP53, Leica P53-CM5P-L, 1:500
Rat anti-F4/80, Abcam ab6640, 1:500
Rabbit anti-pKAP1, Bethyl Laboratories A300-767A, 1:200
Rat anti-HA, Sigma-Aldrich 11867423001, 1:250
Hamster anti-Myc, Absolute Antibody Ab00100-22.0, 1:1000
Rabbit anti-γH2AX, Cell Signaling 9718S, 1:250
Chicken anti-RBFOX3, EMD Millipore ABN91, 1:2000
Rabbit anti-NEUROG2, Cell Signaling 13144S, 1:250
Rabbit anti-SATB2, Abcam ab92446, 1:500
Rabbit anti-LHX2, EMD Millipore ABE1402, 1:2000
Rabbit anti-TP53BP1, Novus Biologicals NB100-304SS, 1:1000
Mouse anti-γH2AX, Millipore Sigma 05-636, 1:500
Rabbit anti-INO80 Qiu et al., 2016, kind gift of J. Landry, 1:1000
Rabbit anti-INO80 Proteintech 18810-1-AP, 1:500
Rabbit anti-INO80 Abcam ab105451, 1:1000
Rabbit anti-GAPDH Santa Cruz Biotechnology sc-25778, 1:1500

Secondary Antibodies:
AlexaFluor 488 AffiniPure Donkey anti-Rabbit IgG (H+L) Jackson ImmunoResearch Labs cat.# 711545152 -141606
AlexaFluor 488 AffiniPure Donkey anti-Rat IgG (H+L) Jackson ImmunoResearch Labs cat.# 712545150 - 128005
AlexaFluor 488 AffiniPure Donkey anti-Goat IgG (H+L) Jackson ImmunoResearch Labs cat.# 705545147 - 128271
AlexaFluor 594 AffiniPure Donkey anti-Mouse IgG (H+L) Jackson ImmunoResearch Labs cat.# 715585150
Cy3 AffiniPure Donkey anti-Rabbit IgG (H+L) Jackson ImmunoResearch Labs cat.# 711165152
Cy3 AffiniPure Donkey anti-Rat IgG (H+L) Jackson ImmunoResearch Labs cat. No. 712165153 - 139289
AlexaFluor 647 AffiniPure Donkey anti-Goat IgG (H+L) Jackson ImmunoResearch Labs cat. No. 705-605-003
AlexaFluor 647 Affinipure Donkey anti-Rabbit IgG (H+L) Jackson ImmunoResearch Labs cat. No. 711-605-152
AlexaFluor 647 Affinipure Donkey anti-Rat IgG (H+L) Jackson ImmunoResearch Labs cat. No. 712-605-153
AlexaFluor 647 Affinipure Donkey anti-Chicken IgG (H+L) Jackson ImmunoResearch Labs cat. No. 703-605-155

Validation

All antibodies that were used have been validated in published studies or by the manufacturer.

Rabbit anti-INO80 Qiu et al., 2016, kind gift of J. Landry Publication: Qiu, et al., BMC Biology (2016). INO80 detected in wild-type mouse ESCs but not Ino80 KO ESCs by Western analysis.
Rabbit anti-INO80 Proteintech 18810-1-AP Maunfacturer: INO80 detected in mouse brain tissue subjected to SDS PAGE followed by western blot.
Rabbit anti-INO80 Abcam ab105451 Publication: Rhee, et al., Nature Communications (2018). INO80 detected in MEFs prepared from E13.5 mouse embryos by western analysis.
Rabbit anti-GAPDH Santa Cruz Biotechnology sc-25778 Publication: Saini, et al., Nature Communications (2020). GAPDH from mouse peritoneal macrophages detected by Western blot.
Chicken anti-MAP2 Novus Biologicals NB300-213 Manufacturer: MAP2 detected by immunofluorescence in mouse primary neuronal cultures.
Rabbit anti-GFP Thermo Fisher A-11122 Publication: Peris et al., Nature Communications (2018). GFP detected by immunofluorescence in cells cultured from E18.5 mouse hippocampi (E18.5).
Chicken anti-GFP Abcam ab13970 Maufacturer: ab13970 staining of GFP detected in mouse olfactory bulb tissue sections by immunohistochemistry.
Rat anti-BCL11B Abcam ab18465 Manufacturer: ab18465 staining of CTIP2 detected in mouse brain tissue sections by immunohistochemistry.
Mouse anti-TLE4 Santa Cruz Biotechnology sc-365406 Publication: Popovitchenko, et al., Nature Communications (2020). TLE4 detected in E17 and P0 mouse brain sections by immunofluorescence.
Rat anti-L1-CAM EMD Millipore MAB5272 Publication: Srivatsa, et al, Nature Communications (2014). L1-CAM detected by immunofluorescence in E18.5 mouse brain section.
Rat anti-EOMES Thermo Fisher 14-4875-80 Publication: Lin, et al., Nature (2016). EOMES detected by immunofluorescence microscopy in E13.5 mouse embryo.
Rabbit anti-SOX2 Millipore/Chemicon AB5603 Publication: Han, et al., Nature Communications (2014). SOX2 detected by immunofluorescence analysis in mouse lung tissue sections.
Goat anti-SOX2 Santa Cruz Biotechnology sc-17320 Publication: Burns, et al., Nature Communications (2015). SOX2 detected by immunofluorescence in P1 mouse utricle.
Rabbit anti-CC3 Cell Signaling 9661S Publication: Tsuchiya, et al., Nature Communications (2019). Cleaved caspase-3 detected by immunofluorescence in mouse primary cortical neurons.

Rabbit anti-CC3-Alexa555 Cell Signaling 9604S Publication: Kammertoens, et al., Nature (2017). Cleaved caspase 3 detected by immunofluorescence in mouse endothelial cells.
Rabbit anti-pHH3-Alexa647 Cell Signaling 3458S Publication: Zhu, et al., Journal of Biological Chemistry (2017). pHH3 detect by immunofluorescence in sections of mouse embryonic tongue.
Rabbit anti-TRP53 Leica P53-CM5P-L Publication: Vermeij, et al., Nature (2016). p53 detected by immunostaining in transverse brain sections of adult mouse cortex.
Rat anti-F4/80 Abcam ab6640 Manufacturer: ab6640 staining of F4/80 detected in mouse spleen tissue sections by immunohistochemistry.
Rabbit anti-pKAP1 Bethyl Laboratories A300-767A Publication: Aguado, et al. Nature Communications (2019). pKAP1 detected by immunohistochemical staining in mouse skin cells.
Rat anti-HA Sigma-Aldrich 11867423001 Publication: Viswanathan, et al., Nature Methods (2015). HA detected by immunofluorescence in brain sections of adult mouse after intracraniel AAV injection.
Hamster anti-Myc Absolute Antibody Ab00100-22.0 Publication: Viswanathan, et al., Nature Methods (2015). c-myc detected by immunofluorescence in brain sections of adult mouse after intracraniel AAV injection.
Rabbit anti-γH2AX Cell Signaling 9718S Publication: Weber, et al., Nature Communications (2019). γH2AX detected by immunohistochemistry in liver tissue of male mice.
Chicken anti-RBFOX3 EMD Millipore ABN91 Manufacturer: NeuN detected by immunostaining in formalin fixed paraffin embedded mouse brain but not negative control.
Rabbit anti-NEUROG2 Cell Signaling 13144S Manufacturer: Neurogenin 2 detected by confocal immunofluorescent analysis of mouse E14.5 cortex.
Rabbit anti-SATB2 Abcam ab92446 Manufacturer: ab92446 detected SATB2 in E18 mouse brain tissue sections by immunohistochemistry.
Rabbit anti-LHX2 EMD Millipore ABE1402 Publication: Folgueras, et al., Cell Stem Cell (2013). LHX2 detected by fluorescent immunohistochemistry in mouse backskin cryosections from wild-type, but not Lhx2 knockout, mice.
Rabbit anti-TP53BP1 Novus Biologicals NB100-304SS Manufacturer: 53BP1 detected in irradiated mouse cochlear spiral ganglion cells in frozen section of fixed material.
Mouse anti-γH2AX Millipore Sigma 05-636 Publication: Sato, et al., Nature Communications (2015). γH2AX foci detected by immunofluorescence in kidney sections of 10 to 20-week-old male mice.

Animals and other organisms

Policy information about studies involving animals; ARRIVE guidelines recommended for reporting animal research

Laboratory animals

Animals of both sexes were used. The ages of the animals used were embryonic day (E) 11.5, E12.5, E13.5, E15.5, E16.5, E17.5, post-day (P) 0, P2, and P7. All ages for each experiment are clearly indicated in figures, legends, and the body of the text. All experiments were carried out in accordance with a protocol approved by the University of Michigan Institutional Animal Care & Use Committee. Mice were maintained on a standard 12 hour day:night cycle, with ad libitum access to food and water, at ambient temperature of 71F and humidity of 37-50%. Mice were maintained on the background as indicated for each strain.
The following transgenic or mutant lines were used at the stated ages and as indicated in figures, legends, and the body of the text:
B6.129(C3)-Ino80tm1.1Jland/J Ino80fl - JAX# 027920
E11.5, E12.5, E13.5, E15.5, E17.5, P0
B6.129P2-Gt(ROSA)26Sortm1(DTA)Lky/J ROSADTA - JAX# 009669
E13.5
B6N.129S6-Gt(ROSA)26Sortm1(CAG-tdTomato*,-EGFP*)Ees/J ROSAnT-nG - JAX# 023537
E11.5, E12.5, E13.5, E15.5, E17.5, P0, P2, P7
B6.129P2-Trp53tm1Brn/J p53fl - JAX# 008462
E13.5, E15.5, P0
B6.129S2-Emx1tm1(cre)Krj/J Emx1IRES-Cre - JAX# 005628
E11.5, E12.5, E13.5, E15.5, E17.5, P0, P2, P7
B6.129P2(Cg)-Foxg1tm1(cre)Skm/J Foxg1Cre - JAX# 006084
E11.5, P0
Neurod6tm1(cre)Kan Neurod6Cre - MGI# 2668659
P0
FVB-Tg(GFAP-cre)25Mes/J Tg(hGFAP-Cre) - JAX# 004600
E15.5, P0
STOCK Brca2tm1Brn/Nci - NCI# 01XB9
E11.5, E12.5, E13.5, E15.5, E17.5, P0

Wild animals

Wild animals were not used in this study.

Field-collected samples

Field-collected samples were not used in this study.

Ethics oversight

All experiments were carried out in compliance with ethical regulations for animal research. Our study protocol was reviewed and approved by the University of Michigan Institutional Animal Care & Use Committee.

Note that full information on the approval of the study protocol must also be provided in the manuscript.
